# Sequence balance minimisation: minimising with unequal treatment allocations

**DOI:** 10.1186/s13063-017-1942-3

**Published:** 2017-05-03

**Authors:** Vichithranie W. Madurasinghe

**Affiliations:** Pragmatic Clinical Trials Unit (PCTU), Centre for Primary Care and Public Health, Blizard Institute, Yvonne Carter Building, 58 Turner Street, London, E1 2AB UK

**Keywords:** Randomisation, Minimisation, Unequal allocation, Randomisation test, Simulation study

## Abstract

**Background:**

Minimisation ensures excellent balance between groups for several prognostic factors, even in small samples. However, its use with unequal allocation ratios has been problematic. This paper describes a new minimisation scheme named *sequence balance minimisation* for unequal treatment allocations.

**Methods:**

Treatment- and factor-balancing properties were assessed in simulation studies for two- and three-arm trials with 1:2 and 1:2:3 allocation ratios. Sample sizes were set 30, 60 and 120. The number of prognostic factors on which to achieve balance was ranged from zero (treatment totals only) to ten with two levels occurring in equal probabilities. Random elements were set at 0.95, 0.9, 0.85, 0.80, 0.7, 0.6 and 0.5. Characteristics of the randomisation distributions and the impact of changing the block size while maintaining the allocation ratio were also examined.

**Results:**

Sequence balance minimisation has good treatment- and factor-balancing capabilities, and the randomisation distribution was centred at zero for all scenarios. The mean and median number of allocations achieved were the same as the number expected in most scenarios, and including additional factors (up to ten) in the minimisation scheme had little impact on treatment balance. Treatment balance tended to depart from the target as the random element was lowered. The variability in allocations achieved increased slightly as the number of factors increased, as the random element was decreased and as the sample size increased. The mean and median factor imbalance remained tightly around zero even when the chosen factor was not included in the minimisation scheme, though the variability was greater. The variability in factor imbalance increased slightly as the random element decreased, as well as when the number of prognostic factors and sample size increased. Increasing block size while maintaining the allocation ratio improved treatment balance notably with little impact on factor imbalance.

**Conclusions:**

Sequence balance minimisation has good treatment- and factor-balancing properties and is particularly useful for small trials seeking to achieve balance across several prognostic factors.

**Electronic supplementary material:**

The online version of this article (doi:10.1186/s13063-017-1942-3) contains supplementary material, which is available to authorized users.

## Background

This work was motivated by the Hepfree trial (https://ukctg.nihr.ac.uk/trials/trial-details/trial-details?trialNumber=ISRCTN54828633), a cluster randomised trial to assess the impact of screening and treating immigrants from ‘at-risk’ ethnic minority communities for chronic viral hepatitis. This was a relatively small trial with 56 clusters, 5 arms, an unequal allocation ratio and a further aim of achieving balance on two important prognostic factors. Minimisation was the preferred allocation method because it is widely recognised to produce good balance between groups for several prognostic factors, even in small samples [1–3]. However, there were concerns regarding what is the most appropriate minimisation procedure for an unequal allocation ratio.

Minimisation is based on a different principle from randomisation [1, 2]. It is a valid alternative to randomisation and has the advantage, especially in trials with a small number of units, that there will be only minor differences between groups in those variables used in the allocation process [1–3]. Such balance is especially desirable where there are strong prognostic factors and modest treatment effects, such as oncology [1] and cluster randomised trials [2].

With minimisation, the treatment assigned to the next participant enrolled in the trial depends (wholly or partly) on the characteristics of those participants already enrolled [1]. The first participant is allocated to receive a treatment at random, and with each subsequent assignment, minimisation works towards finding the treatment that has the minimum imbalance across pre-determined prognostic factors [1, 3, 4]. This is done by calculating an imbalance score for each treatment. The imbalance score is based on the covariate information of the next enrolled participant, all previous allocations and the hypothetical allocation of the next participant to each treatment. Treatment allocation that results in the smallest imbalance score is selected as the preferred allocation; allocations to the other treatments are considered non-preferred. At this point, there are two options. The chosen treatment could simply be taken as the one with the lower score or a random element could be introduced [1]. It is customary to introduce a random element to make the allocations more unpredictable [5]; the preferred treatment is assigned with a higher allocation probability, and non-preferred treatments are given low probabilities [5, 6]. In practise, one has to select that *p* (i.e., allocation probability) which strikes a balance between minimising treatment imbalance and avoiding predictability of treatment assignment [5]. Generally, the probabilities are set at some value higher than 0.5 for the preferred option, and the remaining probability is equally divided among the non-preferred. When there are *K* treatments and *k* = 1 is the preferred treatment, Pocock and Simon [5] suggested using *p1* = *p* and *pk* = (1 − *p*)/(*K* − 1) for *k* = 2,…, *K*, where *p* is some constant which must be greater than 1/*K* for the treatment assignment to be in favour of preferred treatment. If *p* were equal to 1/*K*, then each treatment would have an equal probability of being selected. If *p* were equal to 1, then the preferred treatment would be automatically assigned; the chance of treatment imbalance is minimised, but this may make the procedure too predictable [5]. For any individual trial, there is no obvious decision rule for optimizing one’s choice of *p* value [5]. This entire process is repeated for each new unit entering the trial.

Simulation studies show that under similar scenarios, minimisation provides better-balanced treatment groups than simple or permuted block randomisation and that it can incorporate more prognostic factors than stratified randomisation methods such as permuted blocks within strata [5, 7]. Although this technique has been evaluated and applied in studies with equal allocation to two or more treatment arms, expanding it to unequal allocation is challenging [8].

Han et al. [6] showed that the minimisation process needs to be modified for trials involving unbalanced or unequal treatment allocations. Authors introduced two modifications. In the first modification, called *naive minimisation*, the unequal allocation ratio is accounted for in calculation of the imbalance score. Once the preferred treatment is determined, probability assignment would proceed as for the minimisation process for equal treatment allocations. Han et al. [6] showed, however, that this simple modification can lead to deviations from the target allocation ratio, especially when the allocation probability given to the preferred treatment is relatively low. To avoid this problem, they proposed to account for unequal allocation ratios in determining allocation probabilities. In their second modification, called *biased coin minimisation* (BCM), they varied the allocation probabilities assigned for each treatment when it is the preferred treatment, depending on its allocation ratio.

Kuznetsova and Tymofyeyev [8] showed that although BCM leads to an allocation ratio at the end of the study that is close to the targeted one, it does not preserve the allocation ratio at every allocation step. Those authors argued that if the allocation ratio is not preserved at every step, the probability of allocation to a particular treatment tends to fluctuate in periodic cycles, which provides an opportunity for selection bias throughout enrolment [8]. Though it is questionable whether in practise such fluctuations in probability would lead to selection or evaluation bias in double-blind trials, theoretically, when a sequence of covariates of all randomised patients is known, one can calculate the probability to assign a particular treatment at the next allocation and use this knowledge to introduce selection bias in a double-blind trial [8]. Kuznetsova and Tymofyeyev showed that such fluctuations are higher when the probability to allocate to the preferred treatment is higher. Their method of achieving unequal allocations while preserving the allocation ratio at every allocation, worked by executing an equal allocation to *S* ‘fake’ treatment arms, where *S* = *Q1* + *Q2* + … + *QK. S* is called the *block size*, and *Q1*:*Q2*: …:*QK* is the desired allocation ratio to *K* ≥ 2 treatment groups. In general, as long as the block size *S* remained low, this method showed good balancing properties. However, the authors suggested using other allocation techniques in smaller studies, when the allocation ratio leads to a large block size.

### Minimisation with unequal allocation ratios and randomisation test

The randomisation test is based on the idea that if the given treatment has no effect on the outcome, then the assignment of that treatment is just a kind of arbitrary labelling. It is a simulation-based method that usually begins with choosing a test statistic reflecting the question of interest and calculating it for the original data. Next, the observed test statistic is contrasted with a null distribution, which is generated by randomly allocating the data, calculating the test statistic a greater number of times [9] and computing a *p* value as the proportion of allocations whose test statistic was at least as extreme as that of the original assignment.

Proschan et al. [10] found serious problems with the randomisation test for some examples of unequal allocation minimisation. They pointed out that minimisation achieves better balance than more conventional randomisation schemes by restricting the set of likely randomisation sequences, and when used with unequal allocation, this can lead to some interesting issues. To elaborate this problem, they considered a special case where strict minimisation (i.e., next patient was assigned to the treatment that minimised imbalance deterministically) was assumed and balance was sought on a single covariate. They showed that in a two-arm trial (T vs C) with an allocation ratio of 2:1, within a block of three allocations, there are only two treatment assignment possibilities (i.e., T, C, T with a probability of 2/3 or C, T, T with a probability of 1/3). The third patient was always allocated to T. In contrast, block randomisation assigns all three assignment possibilities—(C, T, T), (T, C, T) and (T, T, C)—with equal probability. Further, unlike in permuted block randomisation, when the control observation (0) and treatment observations (1 and 1) within a block were fixed, the randomisation distribution depended on the treatment assignment actually observed because it depended on the order of the observations; 101 and 011 yield different randomisation distributions. This led to a striking difference between the randomisation distributions under strict minimisation and permuted block randomisation. Under strict minimisation, the randomisation distribution does not necessarily have a mean of 0 (i.e., the mean of the *t* test statistics was not centred at 0). It is important to realize that in minimisation, a randomisation distribution can have a non-zero mean only with unequal allocation [10].

This paper suggests that the problems with randomisation tests in unequal allocation minimisation can be overcome by removing the restrictions imposed on a set of likely randomisation sequences. For example, in the case of a two-arm trial (T vs C) with an allocation ratio of 2:1, this issue could be resolved by achieving all treatment assignment possibilities: (C, T, T), (T, C, T) and (T, T, C). Furthermore, this paper aims to show that such a scheme, named *sequence balance minimisation*, can achieve the desired treatment and factor balance, especially in small trials.

## Methods

### Sequence balance minimisation procedure

There are two essential elements to the minimisation process: (1) measuring the total imbalance of treatment numbers and (2) choosing probabilities for assigning treatments. As with traditional minimisation, sequence balance minimisation starts with calculating the respective imbalance scores. At this point, however, unlike in traditional minimisation, where one treatment is selected and assigned with a higher probability chosen *a priori* (and other treatments are allocated with pre-determined low probabilities), sequence balance minimisation moves on to calculate the respective assigning probabilities for each treatment on the basis of its imbalance score. In essence, sequence balance minimisation combines the two elements rather than considering them as two distinct parts of the allocation process. This paper illustrates the sequence balance minimisation procedure using a numerical example in Table 1. Another important difference to note is that only those allocations (i.e., allocations so far and the number of allocations remaining) within the current allocation block are considered in calculating imbalance scores. An allocation block is defined as the number of allocations equal to the sum of desired allocation ratios.

**Table 1:** Hypothetical distribution of baseline characteristics after 30 patients are enrolled

Characteristic	Treatment 1 (*n* = 10)	Treatment 2 (*n* = 20)
Gender		
Men	7	11
Women	3	9
Ethnic group		
White	6	10
Other	4	10

For simplicity, the new procedure is described using a two-arm trial with a 1:2 allocation ratio, seeking to achieve balance on two factors, gender and ethnic group, each with two levels. The two treatments are denoted by T1 and T2, and the respective allocation ratios are denoted by *r1* and *r2*. The allocation block *S* is defined as the sum of allocation ratios (i.e., *S* = *r1* + *r2*). In this scenario, *S* = 3. Note that it is possible to have an identical allocation ration which would sum to a different allocation block size; for example, an allocation ratio of 2:4 comes out with a different *S* value. The effect of varying the allocation block size in this manner is explored in the next section.

Table 1 shows the numbers with specific baseline characteristics in each treatment group after 30 participants had entered this trial. Suppose the next enrolled participant is a white woman. First, the imbalance scores are calculated for each factor level relevant for the next enrolled participant separately. To do this, each factor is taken in turn, and the imbalance associated with each treatment is calculated as the difference between expected and observed numbers of participants allocated to that treatment divided by the number of allocations remaining in the current allocation block. In this example, with regard to factor 1 (gender), 12 women have already been allocated in 4 allocation blocks (i.e., 12/3). Therefore, the next allocation is the first in a new block with all three allocations remaining (Table 2, column 2). On factor 2 (ethnic group), 16 white participants have entered the trial. So far, five allocation blocks have been completed, and the first allocation in the sixth allocation block is already made to T2 (Table 2, column 7), with only two allocations remaining (i.e., 16/3). Therefore, T1 imbalance for factors 1 and 2 are |1-0|/3 (Table 2, columns 4 and 5) and |1-0|/2, respectively (Table 2, columns 9 and 10). For T2, the imbalance is |2-0|/3 (Table 2, columns 4 and 5) and |2-1|/2 (Table 2, columns 9 and 10) on factors 1 and 2, respectively. This measure is defined as a non-negative value, and the imbalance score for treatments with a greater-than-expected number of allocations are set to zero. Then these individual scores are divided by the sum of all individual treatment imbalances for that factor to derive the adjusted imbalance scores. In this example, adjusted factor 1 imbalance scores are 1/3 and 2/3 (Table 2, column 5), and adjusted factor 2 imbalance scores are 1/2 and 1/2 (Table 2, column 10), respectively. If there is only one prognostic factor on which to achieve balance, these adjusted imbalance scores (denoted by a_i_) provide the respective assignment probabilities for each treatment.

**Table 2:** Illustrative example of sequence balance minimisation

Next allocation	Factor 1: gender (women)					Factor 2: ethnic group (white)					Allocation probabilities
	Number allocated	Number required	Difference (required − allocated)	Imbalance score^a^	Weight	Number allocated	Number required	Difference (required − allocated)	Imbalance score^a^	Weight	
T1	0	1	1 − 0	1/3	0.4	0	1	1 − 0	1/2	0.6	0.42
T2	0	2	2 − 0	2/3	0.57	1	2	2 − 1	1/2	0.43	0.58

^a^If minimising on one factor, imbalance scores provide the respective assigning probabilities

When there is more than one prognostic factor (as in this example), the adjusted factor imbalance score for each treatment is combined across all *n* prognostic factors to derive the appropriate treatment assignment probabilities. In combining, the individual factor imbalance scores are weighted so that factors with lesser numbers of allocations remaining in their current block are given the priority by giving a greater weight. The idea is that when there are fewer allocations remaining, there are fewer opportunities to correct for any imbalances that may occur by chance. The weights are defined as follows:

$$ \frac{{\mathrm{X}}_{\mathrm{i}}}{\sum_{\mathrm{i}=1}^{\mathrm{n}}{\mathrm{X}}_{\mathrm{i}}} $$

$$ \mathrm{where}\ {\mathrm{x}}_{\mathrm{i}}\left\{\begin{array}{l}{a}_{\mathrm{i}}\ /\ \mathrm{treatment}\ \mathrm{allocation}\ \mathrm{ratio},\ \mathrm{for}\ 0 > {a}_{\mathrm{i}} > 1\hfill \\ {}\mathrm{s}\mathrm{um}\ \mathrm{of}\ \mathrm{allocation}\ \mathrm{ratio}\mathrm{s}\ /\ \mathrm{treatment}\ \mathrm{allocation}\ \mathrm{ratio},\ \mathrm{for}\ {a}_{\mathrm{i}} = 0\ \mathrm{or}\ {a}_{\mathrm{i}} = 1\hfill \end{array}\right. $$

The total imbalance for each treatment is calculated by summing the weighted imbalance scores across all prognostic factors. Here, if some prognostic factors are considered more important than others, those could be given a greater weight. Allocation probabilities are calculated by dividing of the each treatment total imbalance scores by overall treatment imbalance score across all treatments. The assignment probabilities for T1 and T2 are 0.42 and 0.58, respectively (Table 2). A random element can be used to minimise the predictability when calculated assignment probability is 1.

### Simulation study

**Fig. 1:**
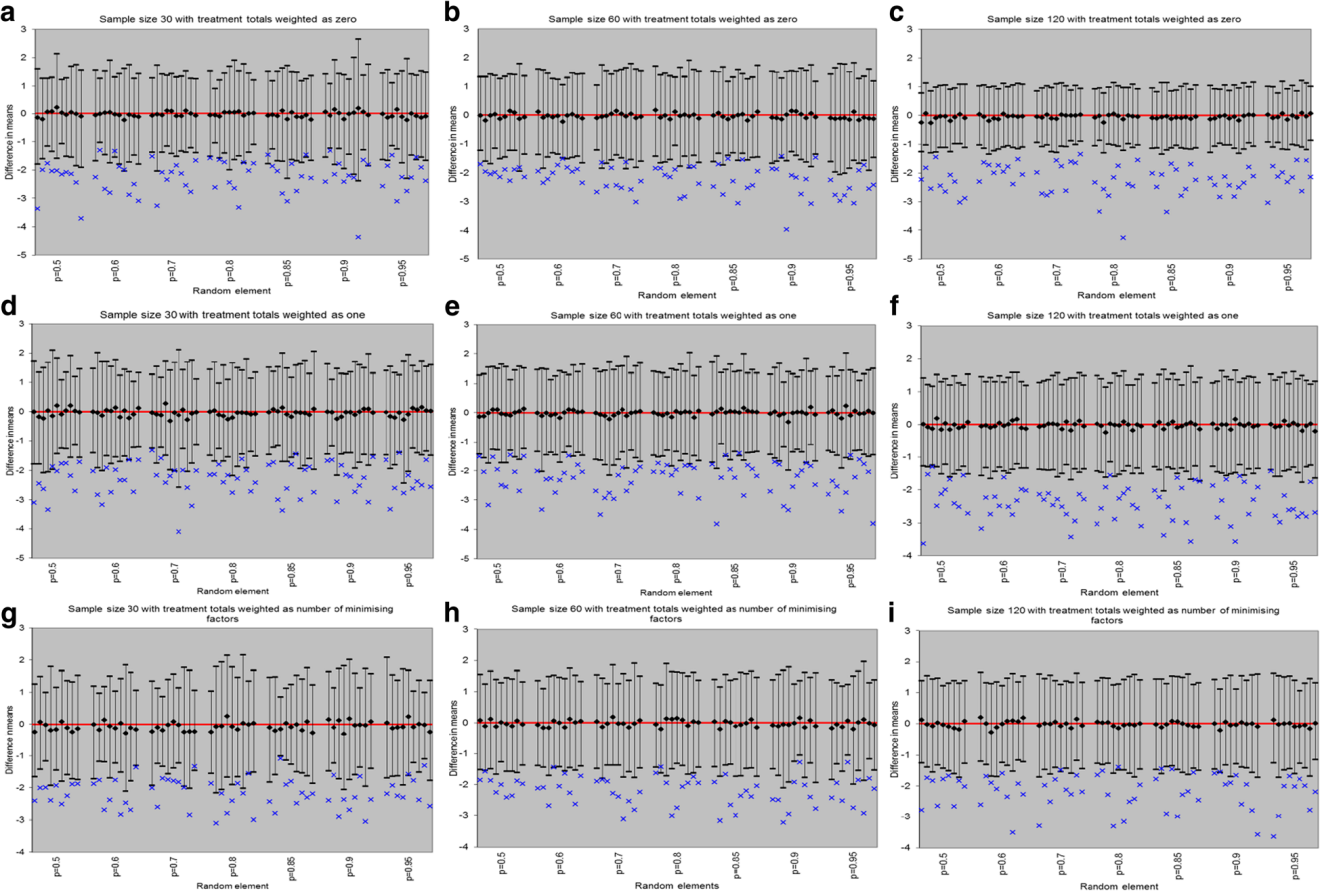
Randomisation distribution with 1:2 allocation ratio and sample sizes 30, 60 and 120

The treatment- and factor-balancing properties of sequence balance minimisation were explored for trials with 1:2 and 1:2:3 allocation ratios, seeking to achieve balance on one to ten prognostic factors, with each factor assumed to have two levels occurring in equal probability. The impact of incorporating treatment totals as an additional prognostic factor in the minimisation scheme was assessed under three weighting schemes: (1) weight of 0 (i.e., treatment totals are not included), (2) weight of 1 (i.e., treatment totals are given the same weight as other prognostic factors) and (3) weighted as the total number of prognostic factors on which to achieve balance (i.e., treatment balance is considered more important by giving it a greater weight). Sample sizes were set at 30, 60 and 120. A random element was used when the assigning probability was calculated as 1. These were set at 0.95, 0.9, 0.85, 0.80, 0.7, 0.6 and 0.5 for the treatment with lowest allocation ratio, and the Han et al. [6] formula was used to calculate the allocation probabilities for non-preferred treatments. For each scenario, 1000 trials were simulated. For each trial, overall treatment and factor imbalance were calculated as the difference between expected and achieved allocations. Characteristics of the randomisation distribution were explored for a continuous outcome (Fig. 1).

As a proof of concept, treatment balance was simulated for the best-case scenario, where treatments were assigned with probability of 1 when the assigning probability was calculated as 1 and only treatment totals were considered in the minimisation scheme, which is the worst-case scenario for factor imbalance when a chosen factor is ignored in the minimisation scheme.

## Results

### Balancing properties and randomisation distribution of sequence balance minimisation

The mean and median numbers of treatment allocations achieved under the best-case scenario were the same as the number expected with the following mean (SE) values: 10 (0), 20 (0) and 40 (0), and median (p1–p99): 10 (10–10), 20 (20–20), and 40 (40–40), for sample sizes 30, 60 and 120, respectively. The factor imbalance: values (mean ([se]): were as follows: 5 (0.04), 10 (0.05) and 20 (0.09), and median (p1– p99): 5 (2–8), 10 (6.5–13.5) and 20 (14–26.5), for sample sizes 30, 60 and 120, respectively.

The overall treatment imbalance in the treatment arm with lower allocation ratio is summarised in Tables 3, 4 and 5. The results show that sequence balance minimisation provide good treatment balance. The overall treatment balance however tends to depart from the target as the random element assumed for the preferred treatment was decreased. This was particularly so, when there was only one minimisation factor. Including additional prognostic factors (up to 10) in the minimisation scheme had little impact on overall treatment balance; the mean and median number of allocations achieved was as same as the expected number. However the variability in allocations achieved increased slightly as the number of prognostic factors increased, and also when the probabilities assumed for random element lowered. This was also the case when the sample size increased. The variability in allocations achieved was reduced as the weight given to treatment totals increased (Table 5).

**Table 3:** Treatment balancing properties of 1:2 sequence balance minimisation with 1 to 10 prognostic factors with 2levels, treatment totals weighted as zero, sample size 30, 60 and 120, random element = 0.95 to 0.5

Random element		Number of factors									
		1	2	3	4	5	6	7	8	9	10
Sample size (*n* = 30), the expected number of participants allocated to the treatment with smallest-allocation ratio (*n* = 10)											
*p* = 0.5	Mean (SE)	10.7 (0.07)	10.5 (0.04)	10.2 (0.03)	10.1 (0.03)	10.1 (0.03)	10.1 (0.03)	10.1 (0.04)	10.1 (0.03)	10.1 (0.03)	10.1 (0.04)
	Median (p1–p99)	11 (6–16)	10 (7.5–14)	10 (8–13)	10 (7–13)	10 (8–13)	10 (8–13)	10 (7–13)	10 (8–13)	10 (8–13)	10 (8–13)
*p* = 0.6	Mean (SE)	10.6 (0.06)	10.4 (0.04)	10.2 (0.03)	10.1 (0.03)	10.1 (0.03)	10.1 (0.03)	10.1 (0.03)	10.1 (0.04)	10.1 (0.04)	10.1 (0.04)
	Median (p1–p99)	11 (6–15)	10 (8–13)	10 (8–13)	10 (8–12.5)	10 (8–12)	10 (8–13)	10 (8–13)	10 (7–13)	10 (7–13)	10 (8–13)
*p* = 0.7	Mean (SE)	10.5 (0.05)	10.3 (0.03)	10.2 (0.03)	10.1 (0.03)	10.1 (0.03)	10.1 (0.03)	10.1 (0.03)	10.1 (0.03)	10.1 (0.04)	10.1 (0.03)
	Median (p1–p99)	10 (7–14)	10 (8–13)	10 (8–12.5)	10 (8–13)	10 (7.5–12)	10 (8–13)	10 (8–13)	10 (8–13)	10 (7.5–13)	10 (7.5–13)
p = 0.8	Mean (SE)	10.3 (0.04)	10.2 (0.03)	10.1 (0.03)	10.1 (0.03)	10.0 (0.03)	10.1 (0.03)	10.1 (0.03)	10.0 (0.03)	10.1 (0.04)	10.1 (0.03)
	Median (p1–p99)	10 (7–14)	10 (8–13)	10 (8–13)	10 (8–13)	10 (8–12)	10 (8–13)	10 (8–12.5)	10 (7.5–12.5)	10 (8–13)	10 (8–13)
*p* = 0.85	Mean (SE)	10.3 (0.03)	10.1 (0.03)	10.1 (0.03)	10.1 (0.03)	10.0 (0.03)	10.1 (0.03)	10.1 (0.03)	10.0 (0.03)	10.1 (0.04)	10.1 (0.03)
	Median (p1–p99)	10 (8–13)	10 (8–12)	10 (8–12)	10 (8–12)	10 (8–12)	10 (8–13)	10 (8–13)	10 (8–13)	10 (7.5–13)	10 (7–13)
*p* = 0.9	Mean (SE)	10.1 (0.03)	10.1 (0.03)	10.0 (0.03)	10.0 (0.03)	10.1 (0.03)	10.1 (0.03)	10.0 (0.03)	10.1 (0.03)	10.1 (0.03)	10.0 (0.03)
	Median (p1–p99)	10 (8–13)	10 (8–12)	10 (8–12)	10 (8–12)	10 (8–12)	10 (8–13)	10 (8–13)	10 (8–13)	10 (7.5–12)	10 (8–13)
*p* = 0.95	Mean (SE)	10.1 (0.02)	10.1 (0.03)	10.0 (0.03)	10.0 (0.03)	10.0 (0.03)	10.0 (0.03)	10.0 (0.03)	10.1 (0.03)	10.1 (0.03)	10.1 (0.04)
	Median (p1–p99)	10 (9–12)	10 (8–12)	10 (8–12)	10 (8–12)	10 (8–12)	10 (7.5–12)	10 (8–12.5)	10 (8–12)	10 (7.5–13)	10 (8–13)
Sample size (*n* = 60), the expected number of participants allocated to the treatment with smallest-allocation ratio (*n* = 20)											
*p* = 0.5	Mean (SE)	21.3 (0.09)	21.0 (0.07)	20.8 (0.06)	20.6 (0.05)	20.3 (0.05)	20.1 (0.05)	20.2 (0.05)	20.1 (0.05)	20.0 (0.05)	20.1 (0.05)
	Median (p1–p99)	21 (14–29)	21 (16.5–26)	21 (17–25.5)	21 (17–25)	20 (17–24)	20 (17–24)	20 (17–24)	20 (17–23)	20 (17–23.5)	20 (17–24)
*p* = 0.6	Mean (SE)	21.1 (0.08)	20.9 (0.06)	20.7 (0.06)	20.4 (0.05)	20.3 (0.05)	20.2 (0.04)	20.1 (0.05)	20.0 (0.05)	20.1 (0.05)	20.2 (0.05)
	Median (p1–p99)	21 (15–27)	21 (17–26)	21 (17–25)	20 (17–24)	20 (17–24)	20 (17–24)	20 (17–24)	20 (17–23)	20 (17–24)	20 (17–24)
*p* = 0.7	Mean (SE)	20.8 (0.07)	20.7 (0.05)	20.5 (0.05)	20.3 (0.05)	20.3 (0.05)	20.1 (0.05)	20.1 (0.05)	20.1 (0.05)	20.1 (0.05)	20.2 (0.05)
	Median (p1–p99)	21 (16–26)	21 (17–24.5)	20 (17–25)	20 (17–24)	20 (17–24)	20 (17–24)	20 (17–24)	20 (17–24)	20 (16.5–24)	20 (16.5–24)
*p* = 0.8	Mean (SE)	20.5 (0.06)	20.4 (0.05)	20.5 (0.05)	20.2 (0.04)	20.1 (0.05)	20.1 (0.05)	20.1 (0.05)	20.0 (0.05)	20.2 (0.05)	20.1 (0.05)
	Median (p1–p99)	21 (17–25)	20 (17–24)	20 (17–24)	20 (17–24)	20 (17–23)	20 (17–23.5)	20 (17–24)	20 (17–23)	20 (17–24)	20 (16–24)
*p* = 0.85	Mean (SE)	20.5 (0.05)	20.3 (0.04)	20.2 (0.05)	20.3 (0.04)	20.2 (0.04)	20.1 (0.04)	20.2 (0.05)	20.0 (0.05)	20.1 (0.05)	20.2 (0.05)
	Median (p1–p99)	20 (17–24)	20 (17–23.5)	20 (17–24)	20 (17–24)	20 (17–23)	20 (17–23.5)	20 (17–23.5)	20 (17–23)	20 (17–24)	20 (17–24)
*p* = 0.9	Mean (SE)	20.2 (0.04)	20.2 (0.04)	20.3 (0.05)	20.2 (0.04)	20.2 (0.04)	20.0 (0.05)	20.1 (0.05)	20.2 (0.05)	20.1 (0.05)	20.1 (0.05)
	Median (p1–p99)	20 (17.5–23)	20 (17–23)	20 (17–24)	20 (17–23)	20 (17–23)	20 (17–24)	20 (17–24)	20 (17–24)	20 (17–24)	20 (17–24)
*p* = 0.95	Mean (SE)	20.1 (0.03)	20.1 (0.03)	20.2 (0.04)	20.1 (0.04)	20.1 (0.04)	20.0 (0.04)	20.1 (0.05)	20.1 (0.05)	20.0 (0.04)	20.3 (0.05)
	Median (p1–p99)	20 (18–23)	20 (18–23)	20 (17–23)	20 (17–23)	20 (17–23)	20 (17–23)	20 (16.5–23)	20 (17–23)	20 (17–24)	20 (17–24)
Sample size (*n* = 120), the expected number of participants allocated to the treatment with smallest-allocation ratio (*n* = 40)											
*p* = 0.5	Mean (SE)	42.2 (0.13)	41.7 (0.10)	41.1 (0.08)	40.7 (0.07)	40.4 (0.07)	40.3 (0.07)	40.4 (0.07)	40.3 (0.07)	40.3 (0.07)	40.3 (0.07)
	Median (p1–p99)	42 (33–52)	42 (35–49)	41 (35–47)	41 (35–46)	40 (36–46)	40 (35–45)	40 (35–46)	40 (36–46)	40 (35–45)	40 (35–45.5)
*p* = 0.6	Mean (SE)	41.8 (0.12)	41.6 (0.09)	40.8 (0.07)	40.5 (0.07)	40.4 (0.07)	40.5 (0.07)	40.3 (0.07)	40.3 (0.07)	40.3 (0.07)	40.4 (0.07)
	Median (p1–p99)	42 (33–50)	41 (35–48)	41 (35–47)	40 (36–45)	40 (36–45)	40 (36–46)	40 (35–45)	40 (35–46)	40 (35–45)	40 (35–46)
*p* = 0.7	Mean (SE)	41.4 (0.10)	41.2 (0.08)	40.7 (0.07)	40.5 (0.07)	40.3 (0.07)	40.4 (0.07)	40.4 (0.07)	40.3 (0.07)	40.2 (0.07)	40.1 (0.07)
	Median (p1–p99)	41 (34–49)	41 (35–47)	41 (36–46)	41 (35–46)	40 (35.5–45)	40 (35–46)	40 (35–45)	40 (35–46)	40 (36–45)	40 (36–46)
*p* = 0.8	Mean (SE)	40.8 (0.08)	40.8 (0.07)	40.6 (0.07)	40.4 (0.07)	40.3 (0.07)	40.2 (0.07)	40.3 (0.07)	40.3 (0.07)	40.4 (0.07)	40.3 (0.07)
	Median (p1–p99)	41 (35.5–46)	41 (35–46)	41 (35–46)	40 (35–45)	40 (35–45)	40 (35–45)	40 (35–46)	40 (35–46)	40 (35–45)	40 (35–45)
*p* = 0.85	Mean (SE)	40.7 (0.07)	40.5 (0.07)	40.5 (0.07)	40.3 (0.07)	40.2 (0.07)	40.3 (0.07)	40.2 (0.07)	40.3 (0.07)	40.3 (0.07)	40.2 (0.07)
	Median (p1–p99)	41 (35–46)	40 (36–45)	40 (36–45)	40 (35.5–45)	40 (35.5–45)	40 (35–45)	40 (35–45.5)	40 (35–45)	40 (35–45)	40 (35–45)
*p* = 0.9	Mean (SE)	40.5 (0.06)	40.5 (0.06)	40.3 (0.06)	40.4 (0.07)	40.2 (0.07)	40.3 (0.07)	40.3 (0.07)	40.2 (0.07)	40.4 (0.07)	40.2 (0.07)
	Median (p1–p99)	40 (36–45)	41 (36–45.5)	40 (36–45)	40 (35–45)	40 (35–45)	40 (35–45)	40 (35–45)	40 (35–45)	40 (35–45)	40 (35–45)
*p* = 0.95	Mean (SE)	40.3 (0.04)	40.3 (0.06)	40.2 (0.06)	40.2 (0.06)	40.2 (0.07)	40.4 (0.06)	40.2 (0.07)	40.2 (0.07)	40.3 (0.07)	40.3 (0.07)
	Median (p1–p99)	40 (38–44)	40 (36–44)	40 (36–44)	40 (36–44.5)	40 (35.5–45)	40 (36–45)	40 (35–46)	40 (35–46)	40 (35–45)	40 (35–45)

Summary statistics from 1000 simulations. Treatment balance under best case scenario: mean (se): 10 (0), 20 (0), 40 (0) and median (p1 – p99): 10 (10 - 10), 20 (20 – 20), 40 (40 – 40) for sample size 30, 60 and 120 respectively

**Table 4:** Treatment balancing properties of 1:2 sequence balance minimisation with 0 (treatment totals only) to 10 prognostic factors with 2 levels, treatment totals weighted as one, sample size 30, 60 and 120, *p* = 0.95 to 0.5

Random element		Treatment totals only	Number of factors									
			1	2	3	4	5	6	7	8	9	10
Sample size (*n* = 30), the expected number of participants allocated to the treatment with smallest-allocation ratio (*n* = 10)												
*p* = 0.5	Mean (SE)	10.9 (0.07)	10.8 (0.06)	10.5 (0.03)	10.2 (0.03)	10.1 (0.03)	10.1 (0.03)	10.1 (0.03)	10.0 (0.04)	10.1 (0.03)	10.0 (0.03)	10.1 (0.03)
	Median (p1–p99)	11 (6–16)	11 (7–15)	10 (8–13)	10 (8–13)	10 (8–13)	10 (8–12)	10 (8–13)	10 (7–13)	10 (8–13)	10 (8–13)	10 (8–13)
*p* = 0.6	Mean (SE)	10.7 (0.06)	10.7 (0.05)	10.4 (0.03)	10.2 (0.03)	10.2 (0.03)	10.1 (0.03)	10.1 (0.03)	10.1 (0.03)	10.1 (0.03)	10.1 (0.03)	10.1 (0.03)
	Median (p1–p99)	11 (7–15)	11 (7–15)	10 (8–13)	10 (8–13)	10 (8–13)	10 (8–13)	10 (8–13)	10 (7–12)	10 (8–12.5)	10 (8–13)	10 (8–13)
*p* = 0.7	Mean (SE)	10.5 (0.05)	10.5 (0.04)	10.3 (0.03)	10.2 (0.03)	10.1 (0.03)	10.1 (0.03)	10.1 (0.03)	10.1 (0.03)	10.1 (0.03)	10.1 (0.03)	10.1 (0.03)
	Median (p1–p99)	10 (6–14)	10 (7–14)	10 (8–13)	10 (8–12.5)	10 (8–12.5)	10 (8–12)	10 (8–12)	10 (8–13)	10 (8–13)	10 (8–13)	10 (8–13)
*p* = 0.8	Mean (SE)	10.3 (0.04)	10.4 (0.03)	10.2 (0.03)	10.1 (0.03)	10.1 (0.03)	10.1 (0.03)	10.1 (0.03)	10.1 (0.03)	10.1 (0.03)	10.2 (0.03)	10.1 (0.04)
	Median (p1–p99)	10 (7–14)	10 (8–13)	10 (8–13)	10 (8–12)	10 (8–13)	10 (8–12)	10 (8–12.5)	10 (8–12)	10 (8–13)	10 (8–13)	10 (7–13)
*p* = 0.85	Mean (SE)	10.3 (0.03)	10.3 (0.03)	10.2 (0.03)	10.1 (0.03)	10.1 (0.03)	10.1 (0.03)	10.0 (0.03)	10.0 (0.03)	10.1 (0.03)	10.1 (0.03)	10.0 (0.03)
	Median (p1–p99)	10 (8–13)	10 (8–13)	10 (8–12)	10 (8–12)	10 (8–12)	10 (8–12)	10 (8–12.5)	10 (8–12)	10 (8–13)	10 (7.5–12.5)	10 (7–13)
*p* = 0.9	Mean (SE)	10.2 (0.03)	10.2 (0.03)	10.1 (0.03)	10.1 (0.03)	10.1 (0.03)	10.0 (0.03)	10.0 (0.03)	10.0 (0.03)	10.1 (0.03)	10.0 (0.04)	10.1 (0.03)
	Median (p1–p99)	10 (8–12)	10 (8–13)	10 (8–12)	10 (8–12.5)	10 (8–12)	10 (8–12)	10 (8–12)	10 (8–12)	10 (7.5–13)	10 (7–13)	10 (8–12)
*p* = 0.95	Mean (SE)	10.1 (0.02)	10.1 (0.02)	10.0 (0.03)	10.1 (0.03)	10.1 (0.03)	10.0 (0.03)	10.1 (0.03)	10.1 (0.03)	10.1 (0.03)	10.1 (0.03)	10.1 (0.03)
	Median (p1–p99)	10 (9–12)	10 (8–12)	10 (8–12)	10 (8–12)	10 (8–12)	10 (8–12)	10 (8–12)	10 (8–12)	10 (8–13)	10 (8–13)	10 (8–13)
Sample size (*n* = 60), the expected number of participants allocated to the treatment with smallest-allocation ratio (*n* = 20)												
*p* = 0.5	Mean (SE)	21.7 (0.09)	21.4 (0.07)	20.9 (0.06)	20.6 (0.06)	20.4 (0.05)	20.3 (0.05)	20 (0.05)	20.1 (0.05)	20.1 (0.05)	20.1 (0.05)	20.1 (0.05)
	Median (p1–p99)	22 (15–29)	21 (16–27)	21 (17–25)	21 (17–26)	20 (17–24)	20 (17–24)	20 (17–23)	20 (17–23.5)	20 (17–23.5)	20 (17–24)	20 (16.5–24)
*p* = 0.6	Mean (SE)	21.3 (0.09)	21.1 (0.07)	20.9 (0.06)	20.5 (0.05)	20.4 (0.04)	20.3 (0.05)	20.1 (0.04)	20.1 (0.05)	20.1 (0.05)	20.2 (0.05)	20.1 (0.05)
	Median (p1–p99)	21 (16–28)	21 (16–27)	21 (17–26)	20 (17–24)	20 (17–24)	20 (17–23.5)	20 (17–24)	20 (17–24)	20 (17–23)	20 (17–24)	20 (17–24)
*p* = 0.7	Mean (SE)	20.9 (0.07)	21.0 (0.06)	20.7 (0.05)	20.4 (0.05)	20.4 (0.04)	20.2 (0.04)	20.1 (0.04)	20.1 (0.05)	20.0 (0.05)	20.1 (0.05)	20.2 (0.05)
	Median (p1–p99)	21 (16–26)	21 (17–26)	21 (17–25)	20 (17–24)	20 (17–24)	20 (17–23)	20 (17–24)	20 (17–23)	20 (17–23)	20 (17–24)	20 (17–24)
*p* = 0.8	Mean (SE)	20.8 (0.06)	20.6 (0.05)	20.4 (0.04)	20.3 (0.05)	20.2 (0.04)	20.2 (0.04)	20.1 (0.04)	20.0 (0.04)	20.0 (0.05)	20.1 (0.05)	20.1 (0.05)
	Median (p1–p99)	21 (17–25)	21 (17–24)	20 (17–24)	20 (17–24)	20 (17–23)	20 (17–24)	20 (17–23)	20 (17–23)	20 (17–24)	20 (17–24)	20 (17–24)
*p* = 0.85	Mean (SE)	20.5 (0.05)	20.5 (0.04)	20.3 (0.04)	20.2 (0.04)	20.2 (0.04)	20.1 (0.04)	20.1 (0.05)	20.1 (0.04)	20.1 (0.05)	20.1 (0.05)	20.2 (0.05)
	Median (p1–p99)	21 (17–24)	20 (18–24)	20 (17–23)	20 (17–24)	20 (17–23)	20 (17–23)	20 (17–23)	20 (17–23)	20 (17–24)	20 (17–24)	20 (17–24)
*p* = 0.9	Mean (SE)	20.4 (0.04)	20.3 (0.04)	20.1 (0.04)	20.2 (0.04)	20.2 (0.04)	20.1 (0.04)	20.1 (0.04)	20.1 (0.05)	20.2 (0.05)	20.1 (0.05)	20.1 (0.05)
	Median (p1–p99)	20 (17–24)	20 (17–23.5)	20 (17–23)	20 (17–23.5)	20 (17–23)	20 (17–23)	20 (17–23)	20 (17–24)	20 (17–24)	20 (17–24)	20 (16–24)
*p* = 0.95	Mean (SE)	20.2 (0.03)	20.2 (0.03)	20.1 (0.04)	20.1 (0.04)	20.1 (0.04)	20.2 (0.04)	20.1 (0.04)	20.1 (0.04)	20.2 (0.05)	20.1 (0.05)	20.1 (0.05)
	Median (p1–p99)	20 (18–23)	20 (18–23)	20 (17–23)	20 (17–23)	20 (17–23)	20 (17–23.5)	20 (16.5–23)	20 (17–23)	20 (17–24)	20 (16.5–24)	20 (17–24)
Sample size (*n* = 120), the expected number of participants allocated to the treatment with smallest-allocation ratio (*n* = 40)												
*p* = 0.5	Mean (SE)	43.4 (0.14)	42.7 (0.09)	41.2 (0.08)	40.8 (0.07)	40.5 (0.07)	40.2 (0.07)	40.3 (0.07)	40.1 (0.07)	40.3 (0.07)	40.2 (0.07)	40.3 (0.07)
	Median (p1–p99)	43 (33–54)	43 (36–49.5)	41 (36–47)	41 (36–46)	40 (36–45)	40 (35–46)	40 (35–45)	40 (35–45.5)	40 (35–45)	40 (35–46)	40 (36–45)
*p* = 0.6	Mean (SE)	42.5 (0.12)	42.1 (0.09)	41.1 (0.07)	40.7 (0.07)	40.3 (0.07)	40.2 (0.07)	40.3 (0.07)	40.2 (0.07)	40.2 (0.07)	40.3 (0.07)	40.4 (0.07)
	Median (p1–p99)	42 (34–51.5)	42 (36–49)	41 (36–46)	41 (36–46)	40 (36–45)	40 (35–45)	40 (35–45)	40 (35–45)	40 (35–45)	40 (35–46)	40 (36–45)
*p* = 0.7	Mean (SE)	42.0 (0.10)	41.6 (0.08)	40.8 (0.07)	40.6 (0.07)	40.2 (0.07)	40.3 (0.07)	40.2 (0.07)	40.3 (0.07)	40.2 (0.07)	40.2 (0.07)	40.2 (0.07)
	Median (p1–p99)	42 (35–49)	42 (36–47.5)	41 (36–46)	41 (36–45)	40 (35–45)	40 (36–45)	40 (35–45)	40 (35.5–45)	40 (35–45)	40 (35–45)	40 (36–45)
*p* = 0.8	Mean (SE)	41.3 (0.08)	41.1 (0.07)	40.6 (0.06)	40.4 (0.06)	40.3 (0.06)	40.1 (0.07)	40.2 (0.06)	40.2 (0.07)	40.2 (0.07)	40.2 (0.07)	40.3 (0.07)
	Median (p1–p99)	41 (36–47)	41 (36–46)	41 (36–45)	40 (36–45)	40 (35.5–45)	40 (35–45)	40 (36–45)	40 (35–45)	40 (35–45)	40 (35.5–45)	40 (36–45)
*p* = 0.85	Mean (SE)	41.0 (0.07)	40.8 (0.06)	40.5 (0.06)	40.4 (0.06)	40.3 (0.07)	40.3 (0.07)	40.2 (0.07)	40.4 (0.07)	40.2 (0.07)	40.3 (0.07)	40.2 (0.07)
	Median (p1–p99)	41 (36–46)	41 (37–45)	40 (36–45)	40 (36–45)	40 (36–45)	40 (35–45)	40 (35–45)	40 (35–46)	40 (35–45)	40 (35–45)	40 (35–45)
*p* = 0.9	Mean (SE)	40.7 (0.06)	40.5 (0.06)	40.2 (0.06)	40.2 (0.06)	40.2 (0.06)	40.1 (0.06)	40.2 (0.07)	40.2 (0.07)	40.2 (0.07)	40.2 (0.07)	40.2 (0.07)
	Median (p1–p99)	41 (36–45)	40 (36–45)	40 (35–45)	40 (36–44)	40 (35–45)	40 (36–45)	40 (36–45.5)	40 (36–45)	40 (35–46)	40 (35–45.5)	40 (35–45.5)
*p* = 0.95	Mean (SE)	40.4 (0.04)	40.3 (0.05)	40.1 (0.06)	40.2 (0.06)	40.1 (0.06)	40.2 (0.07)	40.3 (0.06)	40.1 (0.07)	40.3 (0.07)	40.2 (0.07)	40.3 (0.07)
	Median (p1–p99)	40 (37.5–43.5)	40 (37–44)	40 (36–44)	40 (36–44)	40 (36–45)	40 (35–46)	40 (35–45)	40 (35–45)	40 (35–45)	40 (35–45)	40 (36–45)

Summary statistics from 1000 simulations. Treatment balance under best case scenario: mean (se): 10 (0), 20 (0), 40 (0) and median (p1 – p99): 10 (10 - 10), 20 (20 – 20), 40 (40 – 40) for sample size 30, 60 and 120 respectively

**Table 5:** Treatment balancing properties of 1:2 sequence balance minimisation with 0 (treatment totals only) to 10 prognostic factors with 2 levels, treatment totals weighted as total number of other minimisation factors, sample size 30, 60 and 120, *p* = 0.95 to 0.5

Random element		Number of factors									
		1	2	3	4	5	6	7	8	9	10
Sample size (*n* = 30), the expected number of participants allocated to the treatment with smallest-allocation ratio (*n* = 10)											
*p* = 0.5	Mean (SE)	10.8 (0.06)	10.5 (0.03)	10.2 (0.03)	10.1 (0.03)	10.1 (0.03)	10.1 (0.03)	10.1 (0.03)	10.1 (0.03)	10.1 (0.03)	10.1 (0.03)
	Median (p1–p99)	11 (7–15)	10 (8–13)	10 (8–13)	10 (8–13)	10 (8–12)	10 (8–12)	10 (8–12)	10 (8–12)	10 (8–12)	10 (8–12)
*p* = 0.6	Mean (SE)	10.7 (0.06)	10.5 (0.03)	10.2 (0.03)	10.1 (0.03)	10.1 (0.03)	10.2 (0.03)	10.1 (0.03)	10.1 (0.03)	10.1 (0.03)	10.1 (0.03)
	Median (p1–p99)	11 (7–15)	10 (8–13)	10 (8–13)	10 (8–13)	10 (8–12)	10 (8–12)	10 (8–12.5)	10 (8–13)	10 (8–13)	10 (8–12)
*p* = 0.7	Mean (SE)	10.5 (0.05)	10.3 (0.03)	10.2 (0.03)	10.1 (0.03)	10.1 (0.03)	10.1 (0.03)	10.1 (0.03)	10.1 (0.03)	10.1 (0.03)	10.0 (0.03)
	Median (p1–p99)	10 (7–14)	10 (8–13)	10 (8–12)	10 (8–12)	10 (8–12)	10 (8–12.5)	10 (8–12)	10 (8–12)	10 (8–12)	10 (8–12)
*p* = 0.8	Mean (se)	10.4 (0.03)	10.2 (0.03)	10.1 (0.03)	10.1 (0.03)	10.0 (0.03)	10.1 (0.03)	10.1 (0.03)	10.1 (0.03)	10.1 (0.03)	10.1 (0.03)
	Median (p1–p99)	10 (8–13)	10 (8–12)	10 (8–13)	10 (8–12)	10 (8–12)	10 (8–12)	10 (8–12)	10 (8–12)	10 (8–12)	10 (8–12)
*p* = 0.85	Mean (SE)	10.3 (0.03)	10.2 (0.03)	10.1 (0.03)	10.0 (0.03)	10.0 (0.03)	10.1 (0.03)	10.1 (0.03)	10.1 (0.03)	10.1 (0.03)	10.1 (0.03)
	Median (p1–p99)	10 (8–13)	10 (8–13)	10 (8–12)	10 (8–12)	10 (8–12)	10 (8–12)	10 (8–12)	10 (8–12)	10 (8–12)	10 (8–12)
*p* = 0.9	Mean (SE)	10.2 (0.03)	10.1 (0.03)	10.1 (0.03)	10.0 (0.03)	10.1 (0.03)	10.0 (0.03)	10.1 (0.03)	10.1 (0.03)	10.1 (0.03)	10.1 (0.03)
	Median (p1–p99)	10 (8–13)	10 (8–12)	10 (8–12)	10 (8–12)	10 (8–12)	10 (8–12)	10 (8–12)	10 (8–12)	10 (8–12)	10 (8–12)
*p* = 0.95	Mean (SE)	10.1 (0.02)	10.1 (0.03)	10.1 (0.03)	10.0 (0.03)	10.0 (0.03)	10.1 (0.03)	10.1 (0.03)	10.1 (0.03)	10.1 (0.03)	10.1 (0.03)
	Median (p1–p99)	10 (8–12)	10 (8–12)	10 (8–12)	10 (8–12)	10 (8–12)	10 (8–12)	10 (8–12)	10 (8–12)	10 (8–12)	10 (8–12)
Sample size (*n* = 60), the expected number of participants allocated to the treatment with smallest-allocation ratio (*n* = 20)											
*p* = 0.5	Mean (SE)	21.4 (0.07)	21.0 (0.06)	20.7 (0.05)	20.4 (0.04)	20.2 (0.04)	20.1 (0.04)	20.0 (0.04)	19.9 (0.04)	20.0 (0.04)	20.0 (0.04)
	Median (p1–p99)	21 (16–27)	21 (17–25.5)	21 (17–25)	20 (18–23)	20 (17.5–23)	20 (17–23)	20 (17–23)	20 (17–23)	20 (17–23)	20 (17–23)
*p* = 0.6	Mean (SE)	21.1 (0.07)	20.8 (0.05)	20.5 (0.05)	20.3 (0.04)	20.2 (0.04)	20.1 (0.04)	20.1 (0.04)	20.1 (0.04)	20.1 (0.04)	20.1 (0.04)
	Median (p1–p99)	21 (16–27)	21 (17–25)	20 (17–24)	20 (17.5–23)	20 (17–23)	20 (17–23)	20 (17–23)	20 (17–23)	20 (17–23)	20 (18–23)
*p* = 0.7	Mean (SE)	21.0 (0.06)	20.6 (0.05)	20.4 (0.05)	20.3 (0.04)	20.1 (0.04)	20.1 (0.04)	20.0 (0.04)	20.1 (0.04)	20.1 (0.04)	20.0 (0.04)
	Median (p1–p99)	21 (17–26)	21 (17–24)	20 (17–24)	20 (17–23.5)	20 (17–23)	20 (17–23)	20 (17–23)	20 (17–23)	20 (17–23)	20 (17–23)
*p* = 0.8	Mean (SE)	20.6 (0.05)	20.4 (0.04)	20.2 (0.04)	20.2 (0.04)	20.1 (0.04)	20.0 (0.04)	20.1 (0.04)	20.1 (0.04)	20.0 (0.04)	20.1 (0.04)
	Median (p1–p99)	21 (17–24)	20 (17.5–24)	20 (17–23)	20 (17–23)	20 (17–23)	20 (17–23)	20 (17–23)	20 (17–23)	20 (17–23)	20 (17–23)
*p* = 0.85	Mean (SE)	20.5 (0.04)	20.4 (0.04)	20.2 (0.04)	20.2 (0.03)	20.1 (0.04)	20.0 (0.04)	20.0 (0.04)	20.1 (0.04)	20.1 (0.04)	20.1 (0.04)
	Median (p1–p99)	20 (18–24)	20 (17.5–23)	20 (17–23)	20 (18–23)	20 (17–23)	20 (17–23)	20 (17–23)	20 (17.5–23)	20 (17–23)	20 (17–23)
*p* = 0.9	Mean (SE)	20.3 (0.04)	20.3 (0.04)	20.2 (0.04)	20.1 (0.04)	20.0 (0.04)	20.0 (0.04)	20.0 (0.04)	20.1 (0.04)	20.1 (0.04)	20.1 (0.04)
	Median (p1–p99)	20 (17–23.5)	20 (18–23)	20 (17–23)	20 (17–23)	20 (17–23)	20 (17–23)	20 (17.5–23)	20 (17–23)	20 (17–23)	20 (17–23)
*p* = 0.95	Mean (SE)	20.2 (0.03)	20.1 (0.03)	20.2 (0.04)	20.1 (0.04)	20.0 (0.04)	20.0 (0.04)	20.0 (0.04)	20.0 (0.04)	20.1 (0.04)	20.0 (0.04)
	Median (p1–p99)	20 (18–23)	20 (18–23)	20 (17–23)	20 (17–23)	20 (17–23)	20 (17–23)	20 (17–23)	20 (17–23)	20 (17–23)	20 (17–23)
Sample size (*n* = 120), the expected number of participants allocated to the treatment with smallest-allocation ratio (*n* = 40)											
*p* = 0.5	Mean (SE)	42.7 (0.09)	41.5 (0.07)	40.8 (0.06)	40.4 (0.06)	40.2 (0.06)	40.1 (0.06)	40.2 (0.06)	40.1 (0.06)	40.2 (0.06)	40.2 (0.06)
	Median (p1–p99)	43 (36–49.5)	41 (36–47)	41 (36.5–45)	40 (36–45)	40 (36–45)	40 (36–44)	40 (36–45)	40 (36–44)	40 (36–45)	40 (36–44)
*p* = 0.6	Mean (SE)	42.1 (0.09)	41.1 (0.07)	40.6 (0.06)	40.3 (0.06)	40.0 (0.06)	40.1 (0.06)	40.1 (0.06)	40.1 (0.06)	40.2 (0.06)	40.0 (0.06)
	Median (p1–p99)	42 (36–49)	41 (36–46)	41 (37–45)	40 (36–45)	40 (36–44)	40 (36–44)	40 (36–44)	40 (36–44)	40 (36–45)	40 (36–45)
*p* = 0.7	Mean (SE)	41.6 (0.08)	40.8 (0.06)	40.4 (0.06)	40.2 (0.06)	40.1 (0.06)	40.1 (0.06)	40.1 (0.06)	40.0 (0.06)	40.1 (0.06)	40.1 (0.06)
	Median (p1–p99)	42 (36–47.5)	41 (36–45.5)	40 (36–45)	40 (36–44)	40 (36–44)	40 (36–44)	40 (36–45)	40 (36–45)	40 (36–44)	40 (36–45)
*p* = 0.8	Mean (SE)	41.1 (0.07)	40.5 (0.06)	40.3 (0.06)	40.2 (0.06)	40.1 (0.06)	40.2 (0.06)	40.1 (0.06)	40.0 (0.06)	40.2 (0.06)	40.1 (0.06)
	Median (p1–p99)	41 (36–46)	40 (36–45)	40 (36–44)	40 (36–45)	40 (35.5–44)	40 (36–45)	40 (36–44)	40 (36–45)	40 (36–44)	40 (36–44)
*p* = 0.85	Mean (SE)	40.8 (0.06)	40.4 (0.06)	40.2 (0.05)	40.3 (0.06)	40.1 (0.06)	40.1 (0.06)	40.1 (0.06)	40.2 (0.06)	40.2 (0.06)	40.2 (0.06)
	Median (p1–p99)	41 (37–45)	40 (36–45)	40 (36–44)	40 (36–44)	40 (36–44)	40 (36–44)	40 (36–44)	40 (36–45)	40 (36–44)	40 (36–44.5)
*p* = 0.9	Mean (SE)	40.5 (0.06)	40.3 (0.06)	40.1 (0.05)	40.2 (0.06)	40.1 (0.06)	40.1 (0.06)	40.2 (0.06)	40.0 (0.06)	40.2 (0.06)	40.1 (0.06)
	Median (p1–p99)	40 (36–45)	40 (36–44.5)	40 (36–44)	40 (36–44.5)	40 (36–44)	40 (36–44)	40 (36–45)	40 (36–45)	40 (36–44)	40 (36–45)
*p* = 0.95	Mean (SE)	40.3 (0.05)	40.1 (0.05)	40.0 (0.05)	40.1 (0.06)	40.1 (0.05)	40.2 (0.05)	40.1 (0.06)	40.1 (0.05)	40.2 (0.06)	40.1 (0.06)
	Median (p1–p99)	40 (37–44)	40 (36–44)	40 (36–44)	40 (36–45)	40 (36–44)	40 (36–44)	40 (36–44.5)	40 (36–44)	40 (36–44)	40 (36–45)

Summary statistics from 1000 simulations. Treatment balance under best case scenario: mean (se): 10 (0), 20 (0), 40 (0) and median (p1 – p99): 10 (10 - 10), 20 (20 – 20), 40 (40 – 40) for sample size 30, 60 and 120 respectively

Tables 6, 7 and 8 present the factor imbalance with respect to factor 1 in all scenarios. The treatment totals-only column represents the worst-case imbalance for the chosen factor 1, in which it was ignored in the minimisation scheme. The variability in factor imbalance followed patterns similar to those of treatment imbalance; increasing slightly as the probability assumed for random elements lowered and also as the number of prognostic factors and sample size increased. The mean and median factor imbalance remained tightly around zero even when factor 1 was not included in the minimisation scheme, though the variability in allocation distribution was greater. Further, it was interesting to note that the factor imbalance did not increase as the weight given to treatment totals in the allocation scheme increased (Table 8).

**Table 6:** Factor balancing properties of 1:2 sequence balance minimisation with 1 to 10 prognostic factors with 2 levels, treatment totals weighted as zero, sample size 30, 60 and 120, random element = 0.95 to 0.5. Summary statistics from 1000 simulations

Random element		Number of factors										
		Treatment totals only (worst-case scenario)	1	2	3	4	5	6	7	8	9	10
Sample size (*n* = 30), the expected number of participants allocated with the particular factor level *n* = 5												
*p* = 0.5	Mean (SE)	5.5 (0.05)	5.4 (0.05)	5.2 (0.03)	5.1 (0.03)	5.2 (0.03)	5.2 (0.03)	5.1 (0.03)	5.0 (0.03)	5.0 (0.04)	5.1 (0.04)	5.0 (0.04)
	Median (p1–p99)	5 (2–10)	5 (2–9)	5 (3–8)	5 (3–7)	5 (3–8)	5 (3–8)	5 (3–8)	5 (3–8)	5 (3–8)	5 (2.5–8)	5 (2.5–8)
*p* = 0.6	Mean (SE)	5.3 (0.05)	5.3 (0.04)	5.2 (0.03)	5.1 (0.03)	5.2 (0.03)	5.2 (0.03)	5.1 (0.03)	5.0 (0.03)	5.1 (0.03)	5.0 (0.04)	5.0 (0.04)
	Median (p1–p99)	5 (2–9)	5 (2–8)	5 (3–8)	5 (3–7)	5 (3–8)	5 (3–8)	5 (3–8)	5 (3–8)	5 (3–8)	5 (3–8)	5 (3–8)
*p* = 0.7	Mean (SE)	5.3 (0.05)	5.3 (0.03)	5.1 (0.03)	5.1 (0.03)	5.2 (0.03)	5.2 (0.03)	5.1 (0.03)	5.0 (0.03)	5.0 (0.04)	5.0 (0.04)	5.0 (0.04)
	Median (p1–p99)	5 (2–9)	5 (3–8)	5 (3–7)	5 (3–7)	5 (3–8)	5 (3–8)	5 (3–8)	5 (3–8)	5 (3–8)	5 (2–8)	5 (3–8)
*p* = 0.8	Mean (SE)	5.2 (0.04)	5.1 (0.03)	5.1 (0.03)	5.1 (0.03)	5.3 (0.03)	5.1 (0.03)	5.0 (0.03)	5.0 (0.03)	5.0 (0.04)	5.1 (0.04)	5.0 (0.03)
	Median (p1–p99)	5 (2–8)	5 (3–7)	5 (3–7)	5 (3–7)	5 (3–8)	5 (3–8)	5 (3–8)	5 (3–8)	5 (2–8)	5 (2.5–8)	5 (2–8)
*p* = 0.85	Mean (SE)	5.1 (0.04)	5.1 (0.03)	5.0 (0.03)	5.1 (0.03)	5.2 (0.03)	5.2 (0.03)	5.1 (0.03)	4.9 (0.03)	5.1 (0.04)	5.1 (0.04)	5.1 (0.04)
	Median (p1–p99)	5 (2–8)	5 (3–7)	5 (3–7)	5 (3–7)	5 (3–8)	5 (3–7.5)	5 (3–8)	5 (2–8)	5 (2–8)	5 (2–8)	5 (2–8)
*p* = 0.9	Mean (SE)	5.1 (0.04)	5.1 (0.02)	5.0 (0.03)	5.1 (0.03)	5.2 (0.03)	5.2 (0.03)	5.1 (0.03)	5.0 (0.03)	5.0 (0.04)	5.1 (0.04)	4.9 (0.04)
	Median (p1–p99)	5 (2–8)	5 (3–7)	5 (3–7)	5 (3–7)	5 (3–7)	5 (3–8)	5 (3–8)	5 (2.5–7.5)	5 (2–8)	5 (3–8)	5 (2–8)
*p* = 0.95	Mean (SE)	5.0 (0.04)	5.0 (0.02)	5.0 (0.02)	5.1 (0.03)	5.2 (0.03)	5.1 (0.03)	4.9 (0.03)	5.0 (0.03)	5.0 (0.04)	5.0 (0.04)	5.0 (0.03)
	Median (p1–p99)	5 (2–8)	5 (4–6)	5 (3–7)	5 (3–7)	5 (3–7.5)	5 (3–8)	5 (3–7)	5 (2–7.5)	5 (2–8)	5 (2–8)	5 (2–8)
Sample size (*n* = 60), the expected number of participants allocated with the particular factor level *n* = 10												
*p* = 0.5	Mean (SE)	10.9 (0.07)	10.9 (0.07)	10.6 (0.05)	10.5 (0.05)	10.2 (0.05)	10.2 (0.05)	10.1 (0.05)	10.1 (0.05)	10.1 (0.05)	10.1 (0.05)	10.1 (0.05)
	Median (p1–p99)	11 (6–17)	11 (6–16)	11 (7–14)	10 (7–14)	10 (7–14)	10 (7–14)	10 (7–14)	10 (7–14)	10 (6–14)	10 (6.5–14)	10 (6–14)
*p* = 0.6	Mean (SE)	10.6 (0.07)	10.9 (0.07)	10.6 (0.05)	10.5 (0.05)	10.2 (0.05)	10.2 (0.05)	10.1 (0.05)	10.1 (0.05)	10.1 (0.05)	10.1 (0.05)	10.1 (0.05)
	Median (p1–p99)	11 (6–16)	11 (6–16)	11 (7–14)	10 (7–14)	10 (7–14)	10 (7–14)	10 (7–14)	10 (7–14)	10 (6–14)	10 (6.5–14)	10 (6–14)
*p* = 0.7	Mean (SE)	10.4 (0.06)	10.6 (0.05)	10.4 (0.04)	10.4 (0.05)	10.2 (0.04)	10.3 (0.05)	10.2 (0.05)	10.1 (0.05)	10.2 (0.05)	10.1 (0.06)	10.2 (0.05)
	Median (p1–p99)	10 (6–15)	10 (7–15)	10 (7–14)	10 (7–14)	10 (7–14)	10 (7–14)	10 (7–14)	10 (7–14)	10 (6–14)	10 (6–14)	10 (7–14.5)
*p* = 0.8	Mean (SE)	10.4 (0.06)	10.3 (0.04)	10.3 (0.04)	10.4 (0.04)	10.0 (0.04)	10.2 (0.05)	10.1 (0.05)	10.1 (0.05)	10.1 (0.05)	10.1 (0.05)	10.1 (0.05)
	Median (p1–p99)	10 (6–14)	10 (7–14)	10 (8–14)	10 (7–14)	10 (7–13)	10 (7–14)	10 (7–14)	10 (6–14)	10 (7–14)	10 (6–14)	10 (6–14.5)
*p* = 0.85	Mean (SE)	10.2 (0.05)	10.3 (0.03)	10.2 (0.03)	10.2 (0.04)	10.2 (0.04)	10.2 (0.05)	10.1 (0.05)	10.1 (0.05)	10.1 (0.05)	10.1 (0.05)	10.2 (0.05)
	Median (p1–p99)	10 (6–14)	10 (8–13)	10 (8–13)	10 (7–13)	10 (7–13)	10 (7–14)	10 (7–14)	10 (6.5–14)	10 (7–14)	10 (7–14)	10 (6–14)
*p* = 0.9	Mean (SE)	10.1 (0.05)	10.1 (0.03)	10.2 (0.03)	10.4 (0.04)	10.1 (0.04)	10.2 (0.05)	10.1 (0.05)	10.1 (0.05)	10.1 (0.05)	10.1 (0.05)	10.1 (0.05)
	Median (p1–p99)	10 (6–14)	10 (8–12)	10 (7.5–13)	10 (8–13.5)	10 (7–13)	10 (7–14)	10 (7–13.5)	10 (6.5–14)	10 (6–14)	10 (6.5–14)	10 (6–14)
*p* = 0.95	Mean (SE)	10.1 (0.05)	10.1 (0.02)	10.1 (0.03)	10.3 (0.04)	10.1 (0.04)	10.2 (0.05)	10.1 (0.05)	10.0 (0.05)	10.2 (0.05)	10.1 (0.05)	10.3 (0.05)
	Median (p1–p99)	10 (6–13.5)	10 (9–12)	10 (8–12)	10 (7–13)	10 (7–13.5)	10 (6–14)	10 (7–14)	10 (6.5–14)	10 (7–14)	10 (7–14)	10 (6–14)
Sample size (*n* = 120), the expected number of participants allocated with the particular factor level *n* = 20												
*p* = 0.5	Mean (SE)	22.0 (0.11)	21.0 (0.10)	21.0 (0.08)	20.8 (0.07)	20.5 (0.07)	20.0 (0.08)	20.2 (0.08)	20.4 (0.08)	20.3 (0.08)	20.4 (0.08)	20.6 (0.08)
	Median (p1–p99)	22 (14–30)	21 (14–29)	21 (16–27)	21 (16–26)	21 (15–26)	20 (14–25)	20 (14–26)	20 (15–26.5)	20 (15–26)	20 (14–26)	21 (15–26)
*p* = 0.6	Mean (SE)	21.6 (0.10)	20.8 (0.08)	20.9 (0.07)	20.7 (0.07)	20.4 (0.07)	20.2 (0.08)	20.2 (0.08)	20.3 (0.08)	20.3 (0.08)	20.5 (0.08)	20.6 (0.09)
	Median (p1–p99)	22 (14–30)	21 (15–27)	21 (16–26)	21 (16–26)	20 (16–26)	20 (15–26)	20 (15–26)	20 (15–26)	20 (14–27)	21 (15–26.5)	21 (14–27)
*p* = 0.7	Mean (SE)	21.1 (0.10)	20.7 (0.07)	20.6 (0.07)	20.6 (0.07)	20.4 (0.07)	20.0 (0.08)	20.1 (0.08)	20.3 (0.08)	20.4 (0.08)	20.3 (0.08)	20.6 (0.09)
	Median (p1–p99)	21 (14–29)	21 (16–26)	21 (16–26)	21 (16–25)	20 (15–26.5)	20 (14–26)	20 (15–26)	20 (15–26)	20 (15–26.5)	20 (15–27)	21 (14–26)
*p* = 0.8	Mean (SE)	20.8 (0.09)	20.3 (0.06)	20.4 (0.06)	20.6 (0.07)	20.4 (0.07)	20.1 (0.07)	20.0 (0.08)	20.2 (0.08)	20.4 (0.08)	20.5 (0.08)	20.6 (0.08)
	Median (p1–p99)	21 (14–28)	20 (16–25)	20 (16–25)	21 (16–26)	20 (15.5–25)	20 (15–26)	20 (14–26)	20 (15–26)	20 (14–26.5)	20.5 (15–26)	21 (15–26.5)
*p* = 0.85	Mean (SE)	20.4 (0.09)	20.3 (0.05)	20.3 (0.06)	20.5 (0.06)	20.2 (0.07)	20.1 (0.08)	20.1 (0.08)	20.0 (0.08)	20.3 (0.08)	20.3 (0.08)	20.5 (0.08)
	Median (p1–p99)	20 (14–27)	20 (17–24.5)	20 (16–24)	21 (16–25)	20 (15–25)	20 (15–26)	20 (14.5–25.5)	20 (14–26)	20 (14.5–26)	20 (15–27)	20 (15–27)
*p* = 0.9	Mean (SE)	20.4 (0.09)	20.2 (0.04)	20.3 (0.05)	20.5 (0.06)	20.3 (0.07)	20.2 (0.08)	20.1 (0.08)	20.3 (0.08)	20.3 (0.08)	20.4 (0.08)	20.6 (0.08)
	Median (p1–p99)	20 (14–27)	20 (17–23)	20 (16–25)	21 (16–25)	20 (15–25)	20 (15–26)	20 (14–25.5)	20 (14.5–26.5)	20 (15–26)	20 (15–27)	21 (14–26)
*p* = 0.95	Mean (SE)	20.2 (0.09)	20.1 (0.03)	20.1 (0.05)	20.3 (0.06)	20.2 (0.07)	20.1 (0.07)	20.1 (0.08)	20.2 (0.08)	20.3 (0.08)	20.3 (0.08)	20.6 (0.08)
	Median (p1–p99)	20 (14–27)	20 (18–22)	20 (16–24)	20 (16–25)	20 (15–25)	20 (15–25.5)	20 (15–26)	20 (14.5–26)	20 (14.5–26)	20 (14–26)	21 (14.5–26)

**Table 7:** Factor balancing properties of 1:2 sequence balance minimisation with 0 (treatment totals only) to 10prognostic factors with 2 levels, treatment totals weighted as one, sample size 30, 60 and 120, *p* = 0.95 to 0.5. Summary statistics from 1000 simulations

Random element		Number of factors										
		Treatment totals only (worst-case scenario)	1	2	3	4	5	6	7	8	9	10
Sample size (*n* = 30), the expected number of participants allocated with the particular factor level *n* = 5												
*p* = 0.5	Mean (SE)	5.5 (0.05)	5.3 (0.04)	5.4 (0.03)	5.0 (0.03)	5.2 (0.03)	5.1 (0.03)	5.1 (0.03)	5.0 (0.03)	5.1 (0.04)	5.0 (0.04)	5.0 (0.04)
	Median (p1–p99)	5 (2–10)	5 (2–8)	5 (3–8)	5 (2–7)	5 (3–7.5)	5 (3–8)	5 (3–8)	5 (3–8)	5 (2–8)	5 (2.5–8)	5 (2–8)
*p* = 0.6	Mean (SE)	5.3 (0.05)	5.3 (0.04)	5.3 (0.03)	5.0 (0.03)	5.3 (0.03)	5.2 (0.03)	5.2 (0.04)	5.0 (0.03)	5.1 (0.04)	5.0 (0.04)	5.0 (0.04)
	Median (p1–p99)	5 (2–9)	5 (3–9)	5 (3–8)	5 (3–8)	5 (3–8)	5 (3–8)	5 (3–8)	5 (3–8)	5 (3–8)	5 (3–8)	5 (2.5–8)
*p* = 0.7	Mean (SE)	5.3 (0.05)	5.2 (0.04)	5.3 (0.03)	5.1 (0.03)	5.2 (0.04)	5.2 (0.03)	5.1 (0.03)	5.1 (0.03)	5.1 (0.04)	5.0 (0.04)	5.0 (0.03)
	Median (p1–p99)	5 (2–9)	5 (2.5–8)	5 (3–8)	5 (3–8)	5 (2–8)	5 (3–8)	5 (3–8)	5 (3–8)	5 (3–8)	5 (2.5–8)	5 (2.5–8)
*p* = 0.8	Mean (SE)	5.2 (0.04)	5.2 (0.03)	5.3 (0.03)	5.0 (0.03)	5.2 (0.03)	5.1 (0.03)	5.1 (0.03)	5.1 (0.03)	5.0 (0.03)	5.0 (0.04)	4.9 (0.04)
	Median (p1–p99)	5 (2–8)	5 (3–7)	5 (3–7)	5 (3–7)	5 (3–8)	5 (3–8)	5 (3–8)	5 (2–8)	5 (2.5–8)	5 (2.5–8)	5 (2–8)
*p* = 0.85	Mean (SE)	5.1 (0.04)	5.1 (0.03)	5.2 (0.03)	5.1 (0.03)	5.2 (0.03)	5.1 (0.03)	5.0 (0.04)	4.9 (0.04)	5.1 (0.04)	5.1 (0.03)	5.0 (0.04)
	Median (p1–p99)	5 (2–8)	5 (3–7)	5 (3–7.5)	5 (3–7)	5 (3–8)	5 (3–8)	5 (3–8)	5 (2–8)	5 (2.5–8)	5 (2–8)	5 (2.5–8)
*p* = 0.9	Mean (SE)	5.1 (0.04)	5.1 (0.02)	5.2 (0.03)	5.1 (0.03)	5.2 (0.03)	5.2 (0.03)	5.1 (0.03)	5.0 (0.03)	5.0 (0.04)	5.0 (0.04)	5.1 (0.04)
	Median (p1–p99)	5 (2–8)	5 (3–7)	5 (3–7)	5 (3–8)	5 (3–8)	5 (3–8)	5 (3–7)	5 (3–7.5)	5 (2.5–8)	5 (2–8)	5 (3–8)
*p* = 0.95	Mean (SE)	5.0 (0.04)	5.0 (0.02)	5.1 (0.03)	5.0 (0.03)	5.2 (0.03)	5.2 (0.03)	5.1 (0.03)	5.0 (0.03)	5.1 (0.04)	5.0 (0.04)	5.0 (0.04)
	Median (p1–p99)	5 (2–8)	5 (3–6.5)	5 (3–7)	5 (3–7)	5 (3–8)	5 (3–8)	5 (3–8)	5 (3–8)	5 (3–8)	5 (2–7.5)	5 (2–8)
Sample size (*n* = 60), the expected number of participants allocated with the particular factor level *n* = 10												
*p* = 0.5	Mean (SE)	10.9 (0.07)	10.6 (0.05)	10.4 (0.05)	10.3 (0.05)	10.1 (0.05)	10.1 (0.05)	10.0 (0.05)	9.9 (0.05)	10.0 (0.05)	10.0 (0.05)	10.1 (0.05)
	Median (p1–p99)	11 (6–17)	11 (7–14.5)	10 (7–14)	10 (7–14)	10 (7–14)	10 (7–13.5)	10 (6–13.5)	10 (7–14)	10 (6–14)	10 (6–14)	10 (7–14)
*p* = 0.6	Mean (SE)	10.6 (0.07)	10.5 (0.05)	10.4 (0.04)	10.2 (0.05)	10.1 (0.05)	10.0 (0.05)	10.0 (0.05)	9.9 (0.05)	10.0 (0.05)	10.1 (0.05)	10.2 (0.05)
	Median (p1–p99)	11 (6–16)	10 (7–14)	10 (7.5–14)	10 (7–14)	10 (7–13)	10 (7–14)	10 (6–14)	10 (6–14)	10 (6–14)	10 (6–14)	10 (6.5–14)
*p* = 0.7	Mean (SE)	10.4 (0.06)	10.4 (0.04)	10.4 (0.04)	10.2 (0.05)	10.1 (0.05)	10.0 (0.05)	10.0 (0.05)	10.0 (0.05)	10.0 (0.05)	10.1 (0.05)	10.2 (0.05)
	Median (p1–p99)	10 (6–15)	10 (7–14)	10 (8–14)	10 (7–14)	10 (7–13.5)	10 (7–13)	10 (6.5–13)	10 (7–14)	10 (6–14)	10 (6–14)	10 (6.5–14.5)
*p* = 0.8	Mean (SE)	10.4 (0.06)	10.2 (0.04)	10.3 (0.04)	10.1 (0.04)	10.0 (0.04)	10.0 (0.05)	10.1 (0.05)	9.9 (0.05)	10.0 (0.05)	10.1 (0.05)	10.1 (0.05)
	Median (p1–p99)	10 (6–14)	10 (8–13)	10 (8–13)	10 (7–13)	10 (7–13)	10 (7–14)	10 (7–14)	10 (6–14)	10 (7–14)	10 (6–14)	10 (7–14)
*p* = 0.85	Mean (SE)	10.2 (0.05)	10.2 (0.03)	10.2 (0.04)	10.1 (0.04)	10.0 (0.04)	10.0 (0.05)	10.0 (0.05)	10.0 (0.05)	10.1 (0.05)	10.1 (0.05)	10.2 (0.05)
	Median (p1–p99)	10 (6–14)	10 (8–13)	10 (7–13)	10 (7–13)	10 (7–14)	10 (7–14)	10 (6.5–14)	10 (7–14)	10 (6–14)	10 (6.5–14)	10 (7–14)
*p* = 0.9	Mean (SE)	10.1 (0.05)	10.1 (0.03)	10.2 (0.04)	10.0 (0.04)	9.9 (0.04)	10.0 (0.05)	10.0 (0.05)	10.0 (0.05)	10.1 (0.05)	10.1 (0.05)	10.2 (0.05)
	Median (p1–p99)	10 (6–14)	10 (8–12)	10 (7–13)	10 (7–13)	10 (7–13)	10 (6–14)	10 (7–14)	10 (6–14)	10 (6–14)	10 (6–14)	10 (6–14)
*p* = 0.95	Mean (SE)	10.1 (0.05)	10.0 (0.03)	10.0 (0.03)	10.0 (0.04)	9.9 (0.05)	10.0 (0.05)	10.0 (0.05)	9.9 (0.05)	10.1 (0.05)	10.1 (0.05)	10.2 (0.05)
	Median (p1–p99)	10 (6–13.5)	10 (8–12)	10 (7–12)	10 (7–13)	10 (7–13)	10 (7–13)	10 (7–13)	10 (6–14)	10 (6–14)	10 (6.5–14)	10 (6–14)
Sample size (*n* = 120), the expected number of participants allocated with the particular factor level *n* = 20												
*p* = 0.5	Mean (SE)	22.0 (0.11)	21.7 (0.06)	20.6 (0.07)	20.7 (0.07)	20.3 (0.07)	20.1 (0.08)	20.1 (0.08)	20.1 (0.08)	20.4 (0.08)	20.5 (0.08)	20.5 (0.08)
	Median (p1–p99)	22 (14–30)	22 (17–26)	20 (16–26)	21 (15–26)	20 (15–26)	20 (14–26)	20 (15–26)	20 (14–26)	20 (14–26)	21 (15–27)	20 (14–26)
*p* = 0.6	Mean (SE)	21.6 (0.10)	21.3 (0.07)	20.5 (0.07)	20.6 (0.07)	20.1 (0.07)	20.0 (0.08)	20.1 (0.08)	20.1 (0.08)	20.3 (0.08)	20.4 (0.08)	20.5 (0.08)
	Median (p1–p99)	22 (14–30)	21 (17–26)	20 (16–26)	21 (16–26)	20 (15–25)	20 (14–25.5)	20 (14–26)	20 (14–26)	20 (13.5–26)	20 (15–26)	21 (14–26.5)
*p* = 0.7	Mean (SE)	21.1 (0.10)	21.0 (0.06)	20.4 (0.06)	20.6 (0.07)	20.1 (0.07)	20.1 (0.08)	20.1 (0.08)	20.1 (0.08)	20.4 (0.08)	20.4 (0.09)	20.5 (0.08)
	Median (p1–p99)	21 (14–29)	21 (17–26)	20 (16–25)	21 (16–25)	20 (15–25)	20 (15–26)	20 (14–26)	20 (14–26)	20 (15–26)	20 (14–27)	20 (14.5–27)
*p* = 0.8	Mean (SE)	20.8 (0.09)	20.7 (0.05)	20.3 (0.06)	20.6 (0.06)	20.2 (0.07)	20.0 (0.08)	20.0 (0.08)	20.1 (0.08)	20.3 (0.08)	20.4 (0.08)	20.5 (0.09)
	Median (p1–p99)	21 (14–28)	21 (17–24)	20 (16–25)	21 (16–26)	20 (15–25)	20 (15–25.5)	20 (14–26)	20 (15–26)	20 (15–26)	20 (14–26)	20 (14–27)
*p* = 0.85	Mean (SE)	20.4 (0.09)	20.5 (0.05)	20.2 (0.06)	20.5 (0.07)	20.2 (0.07)	20.2 (0.08)	20.1 (0.08)	20.2 (0.08)	20.4 (0.08)	20.4 (0.08)	20.5 (0.08)
	Median (p1–p99)	20 (14–27)	20 (17–24)	20 (16–25)	20 (16–26)	20 (15–25)	20 (14.5–26)	20 (15–26)	20 (14–26)	20 (15–27)	20 (14–26)	21 (15–27)
*p* = 0.9	Mean (SE)	20.4 (0.09)	20.2 (0.04)	20.0 (0.06)	20.4 (0.07)	20.2 (0.07)	20.1 (0.08)	20.0 (0.08)	20.1 (0.08)	20.3 (0.08)	20.5 (0.08)	20.4 (0.08)
	Median (p1–p99)	20 (14–27)	20 (17–24)	20 (16–24)	20 (16–25)	20 (15–25)	20 (15–26)	20 (14–26)	20 (14–26)	20 (14.5–26)	21 (14–26)	20 (14–26)
*p* = 0.95	Mean (SE)	20.2 (0.09)	20.2 (0.04)	20.0 (0.06)	20.4 (0.06)	20.2 (0.07)	20.1 (0.08)	20.1 (0.08)	20.2 (0.08)	20.4 (0.08)	20.4 (0.08)	20.5 (0.08)
	Median (p1–p99)	20 (14–27)	20 (17–23)	20 (16–24)	20 (16–25)	20 (15–25)	20 (14–26)	20 (15–26)	20 (14–26)	20 (15–26.5)	20.5 (15–26)	20 (14–26)

**Table 8:** Factor balancing properties of 1:2 sequence balance minimisation with 0 (treatment totals only) to 10 prognostic factors with 2 levels, treatment totals weighted as total number of other minimisation factors, *p* = 0.95 to 0.5. Summary statistics from 1000 simulations

Random element		Number of factors										
		Treatment totals only (worst case scenario)	1	2	3	4	5	6	7	8	9	10
Sample size (*n* = 30), the expected number of participants allocated with the particular factor level *n* = 5												
*p* = 0.5	Mean (SE)	5.5 (0.05)	5.3 (0.04)	5.4 (0.03)	5.0 (0.03)	5.2 (0.03)	5.2 (0.03)	5.1 (0.03)	5.0 (0.04)	5.0 (0.04)	5.0 (0.04)	5.0 (0.04)
	Median (p1–p99)	5 (2–10)	5 (2–8)	5 (3–8)	5 (2–8)	5 (3–8)	5 (3–8)	5 (3–8)	5 (2–8)	5 (3–8)	5 (3–8)	5 (3–8)
*p* = 0.6	Mean (SE)	5.3 (0.05)	5.3 (0.04)	5.4 (0.03)	5.0 (0.03)	5.2 (0.03)	5.1 (0.03)	5.1 (0.03)	5.0 (0.03)	5.0 (0.04)	5.1 (0.04)	5.1 (0.04)
	Median (p1–p99)	5 (2–9)	5 (3–9)	5 (3–8)	5 (3–8)	5 (3–8)	5 (3–8)	5 (3–8)	5 (3–8)	5 (3–8)	5 (3–8)	5 (3–8)
*p* = 0.7	Mean (SE)	5.3 (0.05)	5.2 (0.04)	5.2 (0.03)	5.1 (0.03)	5.2 (0.03)	5.2 (0.04)	5.1 (0.04)	5.0 (0.04)	5.0 (0.04)	5.1 (0.04)	5.0 (0.04)
	Median (p1–p99)	5 (2–9)	5 (2.5–8)	5 (3–8)	5 (2.5–7)	5 (3–8)	5 (3–8)	5 (3–8)	5 (2–8)	5 (2–8)	5 (2.5–8)	5 (3–8)
*p* = 0.8	Mean (SE)	5.2 (0.04)	5.2 (0.03)	5.3 (0.03)	5.1 (0.03)	5.1 (0.03)	5.0 (0.03)	5.2 (0.03)	5.1 (0.04)	5.1 (0.04)	5.0 (0.03)	5.1 (0.04)
	Median (p1–p99)	5 (2–8)	5 (3–7)	5 (3–8)	5 (3–8)	5 (3–8)	5 (3–8)	5 (3–8)	5 (2–8)	5 (3–8)	5 (2–7)	5 (2–8)
*p* = 0.85	Mean (SE)	5.1 (0.04)	5.1 (0.03)	5.3 (0.03)	5.0 (0.03)	5.2 (0.03)	5.1 (0.03)	5.1 (0.03)	5.0 (0.03)	5.1 (0.03)	5.0 (0.04)	5.0 (0.04)
	Median (p1–p99)	5 (2–8)	5 (3–7)	5 (3–8)	5 (3–8)	5 (3–8)	5 (3–8)	5 (2–8)	5 (3–8)	5 (3–8)	5 (3–8)	5 (3–8)
*p* = 0.9	Mean (SE)	5.1 (0.04)	5.1 (0.02)	5.2 (0.03)	5.1 (0.03)	5.2 (0.04)	5.2 (0.03)	5.1 (0.03)	5.1 (0.04)	5.0 (0.04)	5.1 (0.04)	5.1 (0.04)
	Median (p1–p99)	5 (2–8)	5 (3–7)	5 (3–7)	5 (3–8)	5 (2–8)	5 (3–8)	5 (3–8)	5 (3–8)	5 (2–8)	5 (3–8)	5 (2–8)
*p* = 0.95	Mean (SE)	5.0 (0.04)	5.0 (0.02)	5.2 (0.03)	5.1 (0.03)	5.2 (0.03)	5.1 (0.03)	5.0 (0.03)	5.0 (0.04)	5.1 (0.03)	5.1 (0.04)	5.0 (0.04)
	Median (p1–p99)	5 (2–8)	5 (3–6.5)	5 (3–7.5)	5 (2.5–8)	5 (3–8)	5 (3–8)	5 (3–8)	5 (2–8)	5 (3–8)	5 (2.5–8)	5 (2–8)
Sample size (*n* = 60), the expected number of participants allocated with the particular factor level *n* = 10												
*p* = 0.5	Mean (SE)	10.9 (0.07)	10.6 (0.05)	10.4 (0.05)	10.1 (0.05)	9.9 (0.05)	9.8 (0.05)	9.9 (0.05)	9.7 (0.05)	9.8 (0.05)	9.9 (0.05)	9.9 (0.05)
	Median (p1–p99)	11 (6–17)	11 (7–14.5)	10 (7–14)	10 (7–14)	10 (7–13.5)	10 (7–13)	10 (7–13)	10 (7–13)	10 (6–13)	10 (6–13)	10 (6–13)
*p* = 0.6	Mean (SE)	10.6 (0.07)	10.5 (0.05)	10.3 (0.04)	10.0 (0.05)	9.8 (0.05)	9.9 (0.05)	9.8 (0.05)	9.8 (0.05)	9.9 (0.05)	9.9 (0.05)	9.9 (0.05)
	Median (p1–p99)	11 (6–16)	10 (7–14)	10 (7–14)	10 (7–13)	10 (6–13)	10 (6–14)	10 (6–13)	10 (7–14)	10 (7–13.5)	10 (6–14)	10 (7–13)
*p* = 0.7	Mean (SE)	10.4 (0.06)	10.4 (0.04)	10.2 (0.04)	10.1 (0.05)	9.9 (0.05)	9.8 (0.05)	9.8 (0.05)	9.7 (0.05)	9.9 (0.05)	9.8 (0.05)	9.9 (0.05)
	Median (p1–p99)	10 (6–15)	10 (7–14)	10 (7–13)	10 (7–14)	10 (7–14)	10 (6–13)	10 (6–13)	10 (6–13)	10 (6–13)	10 (6–14)	10 (6–14)
*p* = 0.8	Mean (SE)	10.4 (0.06)	10.2 (0.04)	10.2 (0.04)	9.9 (0.05)	9.9 (0.04)	9.8 (0.05)	9.8 (0.05)	9.8 (0.05)	9.8 (0.05)	9.9 (0.05)	9.9 (0.05)
	Median (p1–p99)	10 (6–14)	10 (8–13)	10 (7–13)	10 (6–13)	10 (7–13)	10 (6.5–13)	10 (6–13.5)	10 (6–14)	10 (6.5–13)	10 (7–13)	10 (6.5–14)
*p* = 0.85	Mean (SE)	10.2 (0.05)	10.2 (0.03)	10.2 (0.04)	9.9 (0.04)	9.8 (0.04)	9.8 (0.05)	9.8 (0.05)	9.8 (0.05)	9.9 (0.05)	9.9 (0.05)	10.0 (0.05)
	Median (p1–p99)	10 (6–14)	10 (8–13)	10 (7–13)	10 (7–13)	10 (7–13)	10 (7–13)	10 (6–13)	10 (6–13)	10 (7–14)	10 (6–14)	10 (6–14)
*p* = 0.9	Mean (SE)	10.1 (0.05)	10.1 (0.03)	10.1 (0.04)	9.9 (0.04)	9.7 (0.04)	9.8 (0.05)	9.8 (0.05)	9.7 (0.05)	9.8 (0.05)	9.8 (0.05)	10.0 (0.05)
	Median (p1–p99)	10 (6–14)	10 (8–12)	10 (8–13)	10 (7–13)	10 (7–13)	10 (7–13)	10 (7–13)	10 (6–13.5)	10 (6–13)	10 (6–14)	10 (6–14)
*p* = 0.95	Mean (SE)	10.1 (0.05)	10.0 (0.03)	10.1 (0.03)	9.9 (0.04)	9.8 (0.04)	9.7 (0.05)	9.8 (0.05)	9.8 (0.05)	9.8 (0.05)	9.9 (0.05)	9.9 (0.05)
	Median (p1–p99)	10 (6–13.5)	10 (8–12)	10 (8–13)	10 (7–13)	10 (7–13)	10 (6–14)	10 (6–13)	10 (7–13)	10 (6–13)	10 (6–14)	10 (6–14)
Sample size (*n* = 120), the expected number of participants allocated with the particular factor level *n* = 20												
*p* = 0.5	Mean (SE)	22.0 (0.11)	21.7 (0.06)	20.8 (0.07)	20.7 (0.07)	20.3 (0.08)	19.9 (0.08)	20.2 (0.08)	20.2 (0.08)	20.3 (0.08)	20.5 (0.08)	20.4 (0.08)
	Median (p1–p99)	22 (14–30)	22 (17–26)	21 (16–26)	21 (16–26)	20 (15–26)	20 (14–25.5)	20 (15–26)	20 (14–27)	20 (15–26)	20 (15–26.5)	20 (15–27)
*p* = 0.6	Mean (SE)	21.6 (0.10)	21.3 (0.07)	20.6 (0.07)	20.6 (0.07)	20.3 (0.07)	20.1 (0.08)	20.2 (0.08)	20.2 (0.08)	20.4 (0.08)	20.4 (0.08)	20.5 (0.09)
	Median (p1–p99)	22 (14–30)	21 (17–26)	21 (16–25.5)	20 (16–26)	20 (15–25)	20 (14–26)	20 (15–26)	20 (14–26)	20.5 (14–26)	20 (14–26)	21 (14.5–27)
*p* = 0.7	Mean (SE)	21.1 (0.10)	21.0 (0.06)	20.5 (0.07)	20.5 (0.07)	20.3 (0.08)	19.9 (0.07)	20.2 (0.08)	20.1 (0.08)	20.5 (0.08)	20.4 (0.08)	20.5 (0.09)
	Median (p1–p99)	21 (14–29)	21 (17–26)	20 (16–26)	20 (16–26)	20 (15–26)	20 (14.5–25)	20 (14.5–26)	20 (14–26)	20 (15–26.5)	20 (14.5–26)	21 (14.5–27)
*p* = 0.8	Mean (SE)	20.8 (0.09)	20.7 (0.05)	20.3 (0.06)	20.4 (0.07)	20.1 (0.08)	20.1 (0.08)	20.2 (0.08)	20.2 (0.08)	20.2 (0.08)	20.5 (0.08)	20.5 (0.09)
	Median (p1–p99)	21 (14–28)	21 (17–24)	20 (16–25)	20 (15–25)	20 (15–25)	20 (14.5–26)	20 (14–26)	20 (14–27)	20 (15–27)	20 (14.5–27)	20 (14.5–27)
*p* = 0.85	Mean (SE)	20.4 (0.09)	20.5 (0.05)	20.3 (0.06)	20.4 (0.07)	20.3 (0.07)	20.2 (0.08)	20.0 (0.08)	20.2 (0.08)	20.3 (0.08)	20.5 (0.08)	20.4 (0.08)
	Median (p1–p99)	20 (14–27)	20 (17–24)	20 (16–25)	20 (16–26)	20 (15–26)	20 (15–26)	20 (15–26)	20 (14–26)	20 (14–26)	21 (14.5–26.5)	21 (14–26)
*p* = 0.9	Mean (SE)	20.4 (0.09)	20.2 (0.04)	20.2 (0.06)	20.2 (0.07)	20.3 (0.07)	20.0 (0.08)	20.2 (0.08)	20.2 (0.08)	20.2 (0.08)	20.5 (0.08)	20.5 (0.09)
	Median (p1–p99)	20 (14–27)	20 (17–24)	20 (16–25)	20 (15–25)	20 (15–26)	20 (14–26.5)	20 (15–26)	20 (14.5–26)	20 (15–27)	20 (14.5–27)	21 (14.5–27)
*p* = 0.95	Mean (SE)	20.2 (0.09)	20.2 (0.04)	20.1 (0.06)	20.3 (0.07)	20.2 (0.08)	20.0 (0.08)	20.1 (0.08)	20.1 (0.08)	20.6 (0.08)	20.5 (0.08)	20.5 (0.09)
	Median (p1–p99)	20 (14–27)	20 (17–23)	20 (16–24)	20 (15.5–25)	20 (14.5–26)	20 (14–27)	20 (15–26)	20 (14–26)	21 (15–27)	20 (14.5–27)	21 (14.5–27)

Figure 1 shows the randomisation distributions for all scenarios considered in simulations. The mean treatment difference in randomisation distribution was zero, with original overall treatment differences laid outside the 2.5th to 97.5th percentile range. The variability in treatment differences decreased (i.e., 2.5th to 97.5th percentile range narrowing) as the sample size increased. This was particularly notable when the treatment totals were weighted zero in the allocation scheme.

Balancing properties and characteristics of the randomisation distribution for a three-arm trial with 1:2:3 allocation ratio under all scenarios considered before (results presented in Additional file 1) were also examined. The treatment- and factor-balancing properties were quite good. It is clear that as the number of prognostic factors increased, the treatment balance tended to deteriorate gradually. In randomisation distributions, similar to 1:2 allocation, the mean treatment difference was zero for all scenarios.

**Fig. 2:**
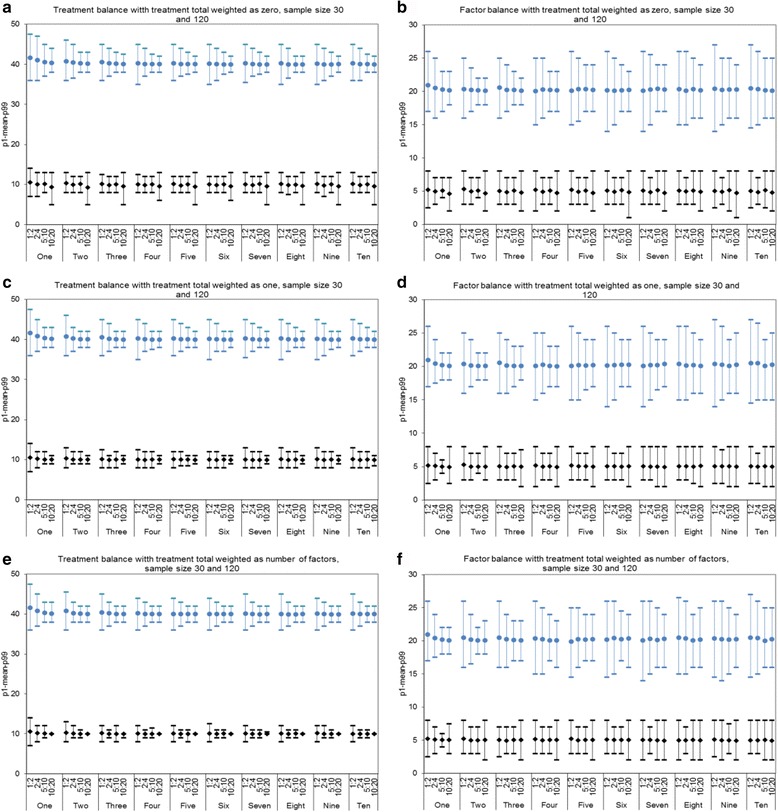
Treatment and factor balance with varying allocation block sizes: 3, 6, 15 and 30, treatment allocation ratio 1:2, sample sizes 30 and 120

The effect of increasing allocation block size from 3 with 1:2 allocation ratio to 6 with 2:4 ratio, to 15 with 5:10 ratio, and to 30 with 10:20 ratio while maintaining the treatment allocation ratio is presented in Fig. 2. Sample sizes were set at 30 and 120. It is clear that as the block size increased, the variability in treatment balance reduced notably. This improved treatment balance appeared to come with little impact on variability in factor balance. The overall mean and median treatment and factor imbalance remained close to zero for all scenarios.

## Discussion

Minimisation is a dynamic randomisation technique that has been used widely in clinical trials for achieving a balance of prognostic factors across treatment groups [6]. The validity of minimisation has been supported by simulation studies in the case of balanced treatment allocation [5]. However, there are problems with using minimisation in unequal allocation ratios, including deviation from the target allocation ratio, particularly when the preferred treatment is assigned a probability value that is relatively low [6] and the randomisation distribution does not necessarily have a mean value of 0 [10]. The latter is not necessarily an issue peculiar to minimisation [11]. It is common to all unequal allocation procedures for which the allocation ratio varies from allocation to allocation [11]. This paper proposes a new minimisation procedure for unequal treatment allocation. The results show that it can achieve the necessary unequal allocation ratio set in trial design, and it performed well in randomisation tests. Further, adding additional prognostic factors in this scheme had little impact on overall treatment and factor balance.

Generally, conventional minimisation algorithms consider only all allocations performed thus far when determining the preferred treatment and assigned with a pre-determined probability. It is customary to use a random probability to make the allocations more unpredictable. The method described here differs with regard to two main aspects of this usual case. Firstly, it includes both previous and remaining allocations within the current allocation block in determining assigning probabilities. Secondly, treatment assignment probabilities are not chosen a priori, but calculated for each allocation separately, which serves to reduce the predictability of allocations. A random element is used only when the calculated treatment assignment probability is 1.

Proschan et al. [10] uncovered serious problems with the randomisation test in unequal allocation minimisation. They showed that minimisation achieves better balance than more conventional randomisation schemes by restricting the set of likely randomisation sequences. Sequence balance minimisation seeks to remove this restriction and allow all possible combinations of treatment assignment, thus producing randomisation distributions with a mean value of zero. This paper provides research evidence to prove the concept; that when treatments are assigned deterministically where required (best-case scenario), sequence balance minimisation achieves the expected number of allocations with the mean of the randomisation distribution centred at 0. The results further show that sequence balance minimisation performs well even when a random element is introduced, as is customary in minimisation.

In covariate adaptive randomisation schemes such as minimisation, when the full sequence of covariates is known, there is a remote possibility that investigators may able to guess what the next treatment will be and introduce selection bias [5, 8]. In sequence balance minimisation, however, full knowledge of covariate sequences is not sufficient; the current treatment allocation sequence and block size would also need to be known. In double-blind trials, investigators are blinded to treatment allocations. To completely eliminate the possibility for selection or evaluation bias, Kuznetsova et al. [8] suggested masking the order of patients’ entry into the trial. In sequence balance minimisation, it is also possible to change the block size.

The accidental bias arising from any non-random order of patients entering the trial (e.g., any time trends in the prognostic mix of patients entering the trial) could bias treatment differences [5, 8, 12]. Block randomisation and block-stratified analysis have been suggested to eliminate such confounding of the results by time trends [12]. Randomising in blocks is a built-in feature of sequence balance minimisation which would reduce the possibility of accidental bias. The extent to which this would eliminate accidental bias could be addressed in future research. Furthermore, this method performed well under equal allocation ratios (results not presented) as well as unequal allocations, and it is also easy to implement, even when the allocation ratio leads to a large block size. However, the present findings indicate that further research is needed to address the question of what is the most appropriate, block size or random element combination, for a particular allocation ratio and sample size, as well as the impact of varying block size and methods for calculating the random element, which would serve to reduce the predictability.

## Conclusions

Minimisation is an allocation scheme particularly useful for trials with small samples because of its ability to ensure balance between the groups on several prognostic factors. The problems with randomisation tests have cast doubts on using minimisation for unequal allocations [10]. This paper describes a new minimisation procedure—sequence balance minimisation—for assigning treatments in unequal allocation ratios. It demonstrated good treatment- and factor-balancing properties, and also its randomisation distribution has a mean value of zero, restoring the usefulness of minimisation, particularly in small trials seeking to achieve balance across several prognostic factors.

## Additional file

Additional file 1: Table S1. Treatment-balancing properties of 1:2:3 sequence balance minimisation with one to ten factors with two levels, treatment totals weighted as zero, random element = 0.95 to 0.5. **Table S2.** Treatment-balancing properties of 1:2:3 sequence balance minimisation with zero (treatment totals only) to ten factors with two levels, treatment totals weighted as one, random element = 0.95 to 0.5. **Table S3**. Treatment-balancing properties of 1:2:3 sequence balance minimisation with zero (treatment totals only) to ten factors with two levels, treatment totals weighted as total number of minimisation factors, random element = 0.95 to 0.5. **Table S4.** Factor-balancing properties of 1:2:3 sequence balance minimisation with one to ten factors with two levels, treatment totals weighted as zero, random element = 0.95 to 0.5. **Table S5.** Factor-balancing properties of 1:2:3 sequence balance minimisation with one to ten factors with two levels, treatment totals weighted as one, random element = 0.95 to 0.5. **Table S6.** Factor-balancing properties of 1:2:3 sequence balance minimisation with one to ten factors with two levels, treatment totals weighted as total number of minimisation factors, random element = 0.95 to 0.5. **Figure S1.** Randomisation distributions with 1:2:3 allocation ratio, mean differences for two treatments with smaller-allocation ratio (*n* = 5 vs 10, 10 vs 20, and 20 vs 40). (DOC 1426 kb)

## Abbreviations

BCM: Biased coin minimisation

## References

[1] Altman DG, Bland JM (2005). Treatment allocation by minimisation. BMJ.

[2] Ivers NM, Halperin IJ, Barnsley J, Grimshaw JM, Shah BR, Tu K (2012). Allocation techniques for balance at baseline in cluster randomized trials: a methodological review. Trials..

[3] Roberts C, Torgerson D (1998). Randomisation methods in controlled trials. BMJ.

[4] Sedgwick P (2013). Treatment allocation by minimisation. BMJ..

[5] Pocock SJ, Simon R (1975). Sequential treatment assignment with balancing for prognostic factors in the controlled clinical trial. Biometrics.

[6] Han B, Enas NH, McEntegart D (2009). Randomization by minimization for unbalanced treatment allocation. Stat Med.

[7] Therneau TM (1993). How many stratification factors are “too many” to use in a randomization plan?. Control Clin Trials.

[8] Kuznetsova OM, Tymofyeyev Y (2012). Preserving the allocation ratio at every allocation with biased coin randomization and minimization in studies with unequal allocation. Stat Med.

[9] Holmes AP. Statistical issues in functional brain mapping. Dissertation, University of Glasgow; 1994

[10] Proschan M, Brittain E, Kammerman L (2011). Minimize the use of minimization with unequal allocation. Biometrics.

[11] Kuznetsova OM (2012). Considerations in the paper by Proschan, Brittain, and Kammerman are not an argument against minimization. In response to Vance W Berger ‘Minimization: not all it’s cracked up to be’, Clin Trials 2011; 8: 443. Clin Trials.

[12] Korn EL, Freidlin B (2011). Outcome-adaptive randomization: is it useful?. J Clin Oncol.

