# Supplementary material for: Sequence balance minimisation: minimising with unequal treatment allocations
Source: Trials. 2017 May 3;18:207. doi: 10.1186/s13063-017-1942-3 (PMC5415754; doi:10.1186/s13063-017-1942-3)

**Appendix A**

Table 1a: Treatment balancing properties of 1:2:3 sequence balance minimisation with 1 to 10 factors with 2 levels, treatment totals weighted as zero, the number of participants allocated to the treatment with smallest-allocation ratio, random element = 0.95 to 0.5. Summary statistics from 1000 simulations. Treatment balance under best case scenario: mean (se): 5 (0), 10 (0), 20 (0) and median (p1 – p99): 5 (5 - 5), 10 (10 – 10), 20 (20 – 20) for sample size 30, 60 and 120 respectively.

| Random element |  | No. of factors | | | | | | | | | |
| --- | --- | --- | --- | --- | --- | --- | --- | --- | --- | --- | --- |
|  |  | 1 | 2 | 3 | 4 | 5 | 6 | 7 | 8 | 9 | 10 |
| Sample size 30, the expected number of participants allocated to the treatment with smallest-allocation ratio (n = 5) | | | | | | | | | | | |
| p=0.5 | Mean (se) | 4.9 (0.03) | 4.8 (0.03) | 4.8 (0.02) | 4.7 (0.02) | 4.7 (0.03) | 4.7 (0.03) | 4.7 (0.03) | 4.6 (0.03) | 4.6 (0.03) | 4.6 (0.03) |
|  | Median  (p1 – p99) | 5 (3 - 7) | 5 (3 - 6.5) | 5 (3 - 6) | 5 (3 - 7) | 5 (3 - 6.5) | 5 (3 - 6) | 5 (3 - 7) | 5 (3 - 6.5) | 5 (3 - 6) | 5 (3 - 7) |
| p=0.6 | Mean (se) | 4.8 (0.03) | 4.9 (0.02) | 4.8 (0.02) | 4.7 (0.03) | 4.7 (0.02) | 4.7 (0.03) | 4.6 (0.03) | 4.6 (0.03) | 4.6 (0.03) | 4.6 (0.03) |
|  | Median  (p1 – p99) | 5 (3 - 7) | 5 (3 - 6) | 5 (3 - 6) | 5 (3 - 7) | 5 (3 - 6.5) | 5 (3 - 7) | 5 (3 - 7) | 5 (3 - 6) | 5 (3 - 6.5) | 5 (3 - 7) |
| p=0.7 | Mean (se) | 4.9 (0.03) | 4.8 (0.02) | 4.7 (0.02) | 4.8 (0.03) | 4.7 (0.03) | 4.7 (0.03) | 4.7 (0.03) | 4.6 (0.03) | 4.6 (0.03) | 4.6 (0.03) |
|  | Median  (p1 – p99) | 5 (3 - 7) | 5 (3 - 6) | 5 (3 - 6) | 5 (3 - 6) | 5 (3 - 7) | 5 (3 - 6) | 5 (3 - 7) | 5 (3 - 7) | 5 (3 - 6) | 5 (3 - 7) |
| p=0.8 | Mean (se) | 4.9 (0.03) | 4.8 (0.02) | 4.8 (0.02) | 4.7 (0.02) | 4.7 (0.03) | 4.7 (0.03) | 4.6 (0.03) | 4.6 (0.03) | 4.6 (0.03) | 4.6 (0.03) |
|  | Median  (p1 – p99) | 5 (3 - 7) | 5 (4 - 6) | 5 (3 - 7) | 5 (3 - 6) | 5 (3 - 6) | 5 (3 - 7) | 5 (3 - 6) | 5 (3 - 6) | 5 (3 - 6) | 5 (3 - 7) |
| p=0.85 | Mean (se) | 4.8 (0.03) | 4.8 (0.02) | 4.7 (0.02) | 4.7 (0.03) | 4.7 (0.02) | 4.7 (0.03) | 4.7 (0.03) | 4.6 (0.03) | 4.5 (0.03) | 4.5 (0.03) |
|  | Median  (p1 – p99) | 5 (3 - 7) | 5 (3 - 6) | 5 (3 - 6) | 5 (3 - 6) | 5 (3 - 6) | 5 (3 - 7) | 5 (3 - 7) | 5 (3 - 7) | 5 (3 - 6) | 5 (3 - 6.5) |
| p=0.9 | Mean (se) | 4.8 (0.02) | 4.8 (0.02) | 4.8 (0.02) | 4.7 (0.03) | 4.7 (0.03) | 4.7 (0.03) | 4.7 (0.03) | 4.7 (0.03) | 4.6 (0.03) | 4.6 (0.03) |
|  | Median  (p1 – p99) | 5 (3 - 6) | 5 (3 - 6) | 5 (3 - 6) | 5 (3 - 6) | 5 (3 - 6) | 5 (3 - 7) | 5 (3 - 7) | 5 (3 - 7) | 5 (3 - 6) | 5 (3 - 7) |
| p=0.95 | Mean (se) | 4.8 (0.02) | 4.8 (0.02) | 4.8 (0.02) | 4.7 (0.03) | 4.7 (0.03) | 4.7 (0.03) | 4.7 (0.03) | 4.6 (0.03) | 4.5 (0.03) | 4.6 (0.03) |
|  | Median  (p1 – p99) | 5 (3 - 6) | 5 (3.5 - 7) | 5 (3 - 6) | 5 (3 - 7) | 5 (3 - 6) | 5 (3 - 6.5) | 5 (3 - 7) | 5 (3 - 6) | 5 (3 - 6) | 5 (3 - 6.5) |
| Sample size 60, the expected number of participants allocated to the treatment with smallest-allocation ratio (n = 10) | | | | | | | | | | | |
| p=0.5 | Mean (se) | 10.1 (0.04) | 10.1 (0.03) | 9.9 (0.03) | 9.8 (0.03) | 9.7 (0.03) | 9.7 (0.03) | 9.6 (0.03) | 9.6 (0.03) | 9.6 (0.03) | 9.5 (0.03) |
|  | Median  (p1 – p99) | 10 (7 - 13) | 10 (9 - 12) | 10 (8 - 12) | 10 (8 - 12) | 10 (7.5 - 12) | 10 (8 - 12) | 10 (8 - 12) | 10 (7 - 12) | 10 (7 - 12) | 9 (7 - 12) |
| p=0.6 | Mean (se) | 10.1 (0.04) | 10.0 (0.03) | 10.0 (0.03) | 9.8 (0.03) | 9.8 (0.03) | 9.7 (0.03) | 9.6 (0.03) | 9.6 (0.03) | 9.6 (0.03) | 9.5 (0.03) |
|  | Median  (p1 – p99) | 10 (7 - 13) | 10 (8 - 12) | 10 (8 - 12) | 10 (8 - 12) | 10 (8 - 12) | 10 (7 - 12) | 10 (7 - 12) | 10 (7 - 12) | 10 (7 - 12) | 10 (7 - 12) |
| p=0.7 | Mean (se) | 10.1 (0.03) | 10.0 (0.02) | 9.9 (0.03) | 9.8 (0.03) | 9.8 (0.03) | 9.7 (0.03) | 9.6 (0.03) | 9.6 (0.03) | 9.6 (0.03) | 9.6 (0.03) |
|  | Median  (p1 – p99) | 10 (8 - 12.5) | 10 (8 - 12) | 10 (8 - 12) | 10 (8 - 12) | 10 (8 - 12) | 10 (8 - 12) | 10 (8 - 12) | 10 (7 - 12) | 10 (7.5 - 12) | 10 (7.5 - 12) |
| p=0.8 | Mean (se) | 10.0 (0.03) | 10.0 (0.02) | 10.0 (0.03) | 9.9 (0.03) | 9.8 (0.03) | 9.7 (0.03) | 9.6 (0.03) | 9.6 (0.03) | 9.5 (0.03) | 9.6 (0.03) |
|  | Median  (p1 – p99) | 10 (8 - 12) | 10 (9 - 12) | 10 (8 - 12) | 10 (8 - 12) | 10 (8 - 12) | 10 (8 - 12) | 10 (7 - 12) | 10 (7 - 12) | 9 (7 - 12) | 10 (7 - 12) |
| p=0.85 | Mean (se) | 10.0 (0.03) | 10.0 (0.02) | 10.0 (0.03) | 9.9 (0.03) | 9.7 (0.03) | 9.7 (0.03) | 9.7 (0.03) | 9.6 (0.03) | 9.6 (0.03) | 9.5 (0.03) |
|  | Median  (p1 – p99) | 10 (8 - 12) | 10 (8 - 12) | 10 (8 - 12) | 10 (8 - 12) | 10 (8 - 12) | 10 (7 - 12) | 10 (8 - 12) | 10 (7 - 12) | 10 (7 - 12) | 9 (7 - 12) |
| p=0.9 | Mean (se) | 10.0 (0.02) | 10.0 (0.02) | 9.9 (0.03) | 9.8 (0.03) | 9.8 (0.03) | 9.7 (0.03) | 9.6 (0.03) | 9.6 (0.03) | 9.6 (0.03) | 9.5 (0.03) |
|  | Median  (p1 – p99) | 10 (8 - 12) | 10 (8 - 11.5) | 10 (8 - 12) | 10 (8 - 12) | 10 (7 - 12) | 10 (8 - 12) | 10 (7 - 12) | 10 (7 - 12) | 10 (7 - 12) | 9 (7 - 12) |
| p=0.95 | Mean (se) | 10.0 (0.02) | 10.0 (0.02) | 9.9 (0.03) | 9.8 (0.03) | 9.8 (0.03) | 9.7 (0.03) | 9.6 (0.03) | 9.6 (0.03) | 9.6 (0.03) | 9.6 (0.03) |
|  | Median  (p1 – p99) | 10 (9 - 11) | 10 (8 - 11.5) | 10 (8 - 12) | 10 (8 - 12) | 10 (8 - 12) | 10 (7.5 - 12) | 10 (7 - 12) | 10 (7.5 - 12) | 10 (7 - 12) | 10 (7 - 12) |
| Sample size 120, the expected number of participants allocated to the treatment with smallest-allocation ratio (n = 20) | | | | | | | | | | | |
| p=0.5 | Mean (se) | 20.1 (0.06) | 20.0 (0.04) | 19.8 (0.04) | 19.7 (0.04) | 19.6 (0.05) | 19.4 (0.04) | 19.2 (0.05) | 19.2 (0.04) | 19.2 (0.04) | 19.1 (0.05) |
|  | Median  (p1 – p99) | 20 (15.5 - 24) | 20 (17 - 23) | 20 (17 - 23) | 20 (17 - 23) | 20 (17 - 23) | 19 (16 - 23) | 19 (16 - 22.5) | 19 (16 - 23) | 19 (16 - 22.5) | 19 (16 - 23) |
| p=0.6 | Mean (se) | 20.1 (0.06) | 20.1 (0.04) | 19.8 (0.04) | 19.8 (0.04) | 19.5 (0.04) | 19.3 (0.05) | 19.2 (0.05) | 19.2 (0.05) | 19.1 (0.04) | 19.1 (0.05) |
|  | Median  (p1 – p99) | 20 (16 - 24) | 20 (17 - 23) | 20 (17 - 23) | 20 (17 - 23) | 19 (16 - 23) | 19 (16 - 23) | 19 (16 - 23) | 19 (16 - 22.5) | 19 (16 - 22) | 19 (16 - 22) |
| p=0.7 | Mean (se) | 20.1 (0.05) | 20.0 (0.04) | 19.8 (0.04) | 19.7 (0.04) | 19.5 (0.04) | 19.4 (0.05) | 19.3 (0.05) | 19.3 (0.04) | 19.1 (0.05) | 19.1 (0.05) |
|  | Median  (p1 – p99) | 20 (17 - 24) | 20 (17 - 23) | 20 (17 - 23) | 20 (17 - 23) | 20 (16.5 - 23) | 19 (16 - 23) | 19 (16 - 23) | 19 (16 - 23) | 19 (16 - 23) | 19 (16 - 22.5) |
| p=0.8 | Mean (se) | 20.0 (0.04) | 20.1 (0.04) | 19.7 (0.04) | 19.7 (0.04) | 19.6 (0.04) | 19.4 (0.04) | 19.3 (0.05) | 19.3 (0.05) | 19.1 (0.04) | 19.0 (0.05) |
|  | Median  (p1 – p99) | 20 (17 - 23) | 20 (17 - 23) | 20 (16 - 23) | 20 (16 - 23) | 20 (16 - 23) | 19 (16 - 23) | 19 (16 - 23) | 19 (16 - 23) | 19 (16 - 22) | 19 (16 - 22.5) |
| p=0.85 | Mean (se) | 20.0 (0.03) | 20.0 (0.04) | 19.8 (0.04) | 19.7 (0.04) | 19.6 (0.04) | 19.5 (0.05) | 19.2 (0.05) | 19.1 (0.05) | 19.2 (0.04) | 19.1 (0.05) |
|  | Median  (p1 – p99) | 20 (17.5 - 23) | 20 (17.5 - 23) | 20 (17 - 23) | 20 (17 - 23) | 20 (16.5 - 23) | 19 (16 - 23) | 19 (16 - 22.5) | 19 (16 - 23) | 19 (16 - 22) | 19 (16 - 22) |
| p=0.9 | Mean (se) | 20.0 (0.03) | 20.0 (0.04) | 19.7 (0.04) | 19.6 (0.05) | 19.6 (0.04) | 19.4 (0.05) | 19.3 (0.05) | 19.2 (0.05) | 19.1 (0.04) | 19.1 (0.05) |
|  | Median  (p1 – p99) | 20 (18 - 22) | 20 (17 - 23) | 20 (17 - 23) | 20 (16 - 23) | 20 (16.5 - 23) | 19 (16 - 23) | 19 (16 - 23) | 19 (16 - 23) | 19 (16 - 22) | 19 (16 - 23) |
| p=0.95 | Mean (se) | 20.0 (0.02) | 20.0 (0.04) | 19.8 (0.04) | 19.8 (0.04) | 19.5 (0.04) | 19.4 (0.05) | 19.3 (0.05) | 19.1 (0.05) | 19.1 (0.04) | 19.0 (0.04) |
|  | Median  (p1 – p99) | 20 (18 - 22) | 20 (17 - 23) | 20 (17 - 23) | 20 (17 - 23) | 19 (16.5 - 23) | 19 (16 - 23) | 19 (16 - 23) | 19 (16 - 22.5) | 19 (16 - 22) | 19 (16 - 22.5) |

Table 1b: Treatment balancing properties of 1:2:3 sequence balance minimisation with 0 (treatment totals only) to 10 factors with 2 levels, treatment totals weighted as one, the number of participants allocated to the treatment with smallest allocation ratio, p = 0.95 to 0.5. Summary statistics from 1000 simulations. Treatment balance under best case scenario: mean (se): 5 (0), 10 (0), 20 (0) and median (p1 – p99): 5 (5 - 5), 10 (10 – 10), 20 (20 – 20) for sample size 30, 60 and 120 respectively.

| Random element |  | Treatment totals only | No. of factors | | | | | | | | | |
| --- | --- | --- | --- | --- | --- | --- | --- | --- | --- | --- | --- | --- |
|  |  |  | 1 | 2 | 3 | 4 | 5 | 6 | 7 | 8 | 9 | 10 |
| Sample size 30, the expected number of participants allocated to the treatment with smallest-allocation ratio (n = 5) | | | | | | | | | | | | |
| p=0.5 | Mean (se) | 5.2 (0.03) | 5.0 (0.02) | 5.0 (0.02) | 4.9 (0.02) | 4.9 (0.02) | 4.9 (0.02) | 4.9 (0.02) | 4.8 (0.02) | 4.8 (0.02) | 4.7 (0.02) | 4.7 (0.02) |
|  | Median  (p1 – p99) | 5 (3 - 8) | 5 (4 - 7) | 5 (4 - 6) | 5 (4 - 6) | 5 (3 - 6) | 5 (4 - 6) | 5 (3 - 7) | 5 (3 - 7) | 5 (3 - 7) | 5 (3 - 6) | 5 (3 - 6) |
| p=0.6 | Mean (se) | 5.1 (0.03) | 5.0 (0.02) | 5.0 (0.02) | 4.9 (0.02) | 4.9 (0.02) | 4.9 (0.02) | 4.8 (0.02) | 4.8 (0.02) | 4.7 (0.03) | 4.7 (0.02) | 4.7 (0.02) |
|  | Median  (p1 – p99) | 5 (3 - 7) | 5 (3.5 - 7) | 5 (4 - 6) | 5 (4 - 6) | 5 (4 - 6) | 5 (3 - 6) | 5 (3 - 7) | 5 (3 - 6) | 5 (3 - 6) | 5 (3 - 6) | 5 (3 - 6) |
| p=0.7 | Mean (se) | 5.1 (0.03) | 5.0 (0.02) | 5.0 (0.02) | 4.9 (0.02) | 4.9 (0.02) | 4.9 (0.02) | 4.9 (0.02) | 4.8 (0.02) | 4.8 (0.02) | 4.7 (0.03) | 4.7 (0.02) |
|  | Median  (p1 – p99) | 5 (3 - 7) | 5 (4 - 7) | 5 (4 - 6) | 5 (4 - 6) | 5 (4 - 6) | 5 (3 - 6) | 5 (3 - 6) | 5 (3 - 6) | 5 (3 - 7) | 5 (3 - 6) | 5 (3 - 6) |
| p=0.8 | Mean (se) | 5.1 (0.02) | 5.0 (0.02) | 5.0 (0.02) | 4.9 (0.02) | 4.9 (0.02) | 4.9 (0.02) | 4.9 (0.02) | 4.8 (0.02) | 4.8 (0.02) | 4.7 (0.02) | 4.7 (0.02) |
|  | Median  (p1 – p99) | 5 (4 - 7) | 5 (4 - 6) | 5 (4 - 6) | 5 (4 - 6) | 5 (4 - 6) | 5 (3.5 - 6) | 5 (3 - 7) | 5 (3 - 6.5) | 5 (3 - 6.5) | 5 (3 - 6) | 5 (3 - 6) |
| p=0.85 | Mean (se) | 5.1 (0.02) | 5.0 (0.01) | 5.0 (0.02) | 4.9 (0.02) | 4.9 (0.02) | 4.9 (0.02) | 4.9 (0.02) | 4.8 (0.02) | 4.8 (0.03) | 4.7 (0.03) | 4.7 (0.02) |
|  | Median  (p1 – p99) | 5 (4 - 7) | 5 (4 - 6) | 5 (4 - 6) | 5 (4 - 6) | 5 (3 - 6) | 5 (3 - 6) | 5 (3 - 6) | 5 (3 - 6) | 5 (3 - 6.5) | 5 (3 - 6.5) | 5 (3 - 6) |
| p=0.9 | Mean (se) | 5.1 (0.02) | 5.0 (0.01) | 4.9 (0.02) | 4.9 (0.02) | 4.9 (0.02) | 4.9 (0.02) | 4.8 (0.02) | 4.8 (0.02) | 4.8 (0.02) | 4.7 (0.03) | 4.7 (0.02) |
|  | Median  (p1 – p99) | 5 (4 - 6) | 5 (4 - 6) | 5 (4 - 6) | 5 (4 - 6) | 5 (4 - 6) | 5 (4 - 6) | 5 (3 - 6.5) | 5 (3 - 7) | 5 (3 - 6) | 5 (3 - 7) | 5 (3 - 6) |
| p=0.95 | Mean (se) | 5.0 (0.01) | 5.0 (0.01) | 4.9 (0.02) | 4.9 (0.02) | 4.9 (0.02) | 4.9 (0.02) | 4.9 (0.02) | 4.8 (0.02) | 4.7 (0.02) | 4.7 (0.02) | 4.7 (0.03) |
|  | Median  (p1 – p99) | 5 (4 - 6) | 5 (4 - 6) | 5 (4 - 6) | 5 (4 - 6) | 5 (4 - 6) | 5 (3 - 7) | 5 (3 - 7) | 5 (3 - 6.5) | 5 (3 - 6) | 5 (3 - 6) | 5 (3 - 6) |
| Sample size 60, the expected number of participants allocated to the treatment with smallest-allocation ratio (n = 10) | | | | | | | | | | | | |
| p=0.5 | Mean (se) | 10.3 (0.04) | 10.2 (0.03) | 10.0 (0.03) | 9.9 (0.03) | 9.8 (0.03) | 9.8 (0.03) | 9.7 (0.03) | 9.6 (0.03) | 9.6 (0.03) | 9.5 (0.03) | 9.6 (0.03) |
|  | Median  (p1 – p99) | 10 (7 - 14) | 10 (8 - 12) | 10 (8 - 12) | 10 (8 - 12) | 10 (8 - 12) | 10 (8 - 12) | 10 (8 - 12) | 10 (7.5 - 12) | 10 (7 - 12) | 10 (7 - 12) | 10 (8 - 12) |
| p=0.6 | Mean (se) | 10.2 (0.04) | 10.1 (0.03) | 10.0 (0.03) | 9.9 (0.03) | 9.9 (0.03) | 9.7 (0.03) | 9.7 (0.03) | 9.6 (0.03) | 9.6 (0.03) | 9.6 (0.03) | 9.5 (0.03) |
|  | Median  (p1 – p99) | 10 (7 - 13) | 10 (8 - 12) | 10 (8 - 12) | 10 (8 - 12) | 10 (8 - 12) | 10 (8 - 12) | 10 (8 - 12) | 10 (7 - 12) | 10 (7 - 12) | 10 (7 - 12) | 9 (8 - 12) |
| p=0.7 | Mean (se) | 10.2 (0.03) | 10.1 (0.02) | 10.0 (0.02) | 9.9 (0.03) | 9.8 (0.03) | 9.7 (0.03) | 9.7 (0.03) | 9.6 (0.03) | 9.6 (0.03) | 9.6 (0.03) | 9.6 (0.03) |
|  | Median  (p1 – p99) | 10 (8 - 13) | 10 (8 - 12) | 10 (8 - 12) | 10 (8 - 12) | 10 (8 - 12) | 10 (8 - 12) | 10 (8 - 12) | 10 (7 - 12) | 10 (8 - 12) | 10 (7 - 12) | 10 (7.5 - 12) |
| p=0.8 | Mean (se) | 10.1 (0.03) | 10.1 (0.02) | 10.0 (0.02) | 9.9 (0.03) | 9.8 (0.03) | 9.8 (0.03) | 9.7 (0.03) | 9.7 (0.03) | 9.5 (0.03) | 9.6 (0.03) | 9.6 (0.03) |
|  | Median  (p1 – p99) | 10 (8 - 13) | 10 (9 - 12) | 10 (8 - 12) | 10 (8 - 12) | 10 (8 - 12) | 10 (8 - 12) | 10 (8 - 12) | 10 (8 - 12) | 10 (7 - 12) | 10 (7 - 12) | 10 (7 - 12) |
| p=0.85 | Mean (se) | 10.1 (0.03) | 10.1 (0.02) | 10.0 (0.02) | 9.9 (0.03) | 9.8 (0.03) | 9.7 (0.03) | 9.7 (0.03) | 9.6 (0.03) | 9.6 (0.03) | 9.7 (0.03) | 9.6 (0.03) |
|  | Median  (p1 – p99) | 10 (8 - 12) | 10 (9 - 12) | 10 (8 - 12) | 10 (8 - 12) | 10 (8 - 12) | 10 (8 - 12) | 10 (8 - 12) | 10 (8 - 12) | 10 (7.5 - 12) | 10 (8 - 12) | 10 (8 - 12) |
| p=0.9 | Mean (se) | 10.1 (0.02) | 10.0 (0.02) | 10.0 (0.02) | 9.9 (0.03) | 9.8 (0.03) | 9.8 (0.03) | 9.7 (0.03) | 9.6 (0.03) | 9.6 (0.03) | 9.6 (0.03) | 9.6 (0.03) |
|  | Median  (p1 – p99) | 10 (8 - 12) | 10 (9 - 11) | 10 (8 - 12) | 10 (8 - 12) | 10 (8 - 12) | 10 (8 - 12) | 10 (8 - 12) | 10 (7 - 12) | 10 (8 - 12) | 10 (7 - 12) | 10 (7 - 12) |
| p=0.95 | Mean (se) | 10.0 (0.01) | 10.0 (0.02) | 10.0 (0.02) | 9.9 (0.03) | 9.8 (0.03) | 9.7 (0.03) | 9.7 (0.03) | 9.6 (0.03) | 9.6 (0.03) | 9.6 (0.03) | 9.6 (0.03) |
|  | Median  (p1 – p99) | 10 (9 - 11) | 10 (9 - 11) | 10 (8 - 11.5) | 10 (8 - 12) | 10 (8 - 12) | 10 (8 - 12) | 10 (8 - 12) | 10 (7 - 12) | 10 (7 - 12) | 10 (7 - 12) | 10 (7.5 - 12) |
| Sample size 120, the expected number of participants allocated to the treatment with smallest-allocation ratio (n = 20) | | | | | | | | | | | | |
| p=0.5 | Mean (se) | 20.7 (0.06) | 20.2 (0.03) | 20.0 (0.03) | 19.7 (0.04) | 19.6 (0.04) | 19.5 (0.04) | 19.4 (0.05) | 19.3 (0.04) | 19.2 (0.04) | 19.1 (0.04) | 19.1 (0.05) |
|  | Median  (p1 – p99) | 21 (16 - 26) | 20 (18 - 23) | 20 (17 - 22) | 20 (17 - 23) | 20 (16 - 23) | 20 (17 - 23) | 19 (16 - 23) | 19 (16 - 22) | 19 (16 - 22.5) | 19 (16 - 22.5) | 19 (16 - 23) |
| p=0.6 | Mean (se) | 20.5 (0.06) | 20.1 (0.03) | 20.0 (0.03) | 19.8 (0.04) | 19.7 (0.04) | 19.5 (0.04) | 19.3 (0.05) | 19.2 (0.04) | 19.2 (0.05) | 19.2 (0.04) | 19.0 (0.05) |
|  | Median  (p1 – p99) | 21 (16 - 25) | 20 (18 - 23) | 20 (17 - 23) | 20 (17 - 23) | 20 (17 - 23) | 19 (16 - 23) | 19 (16 - 23) | 19 (16 - 22.5) | 19 (16 - 23) | 19 (16 - 23) | 19 (16 - 23) |
| p=0.7 | Mean (se) | 20.5 (0.05) | 20.1 (0.03) | 20.0 (0.04) | 19.8 (0.04) | 19.6 (0.04) | 19.5 (0.04) | 19.4 (0.04) | 19.2 (0.04) | 19.2 (0.04) | 19.1 (0.05) | 19.2 (0.04) |
|  | Median  (p1 – p99) | 20 (17 - 24) | 20 (18 - 22) | 20 (17 - 23) | 20 (17 - 23) | 20 (16.5 - 23) | 20 (16 - 23) | 19 (16 - 23) | 19 (16 - 23) | 19 (16 - 22) | 19 (16 - 23) | 19 (16 - 23) |
| p=0.8 | Mean (se) | 20.3 (0.04) | 20.0 (0.03) | 19.9 (0.03) | 19.8 (0.04) | 19.7 (0.04) | 19.5 (0.04) | 19.4 (0.04) | 19.3 (0.04) | 19.2 (0.04) | 19.1 (0.04) | 19.1 (0.05) |
|  | Median  (p1 – p99) | 20 (18 - 24) | 20 (18 - 22) | 20 (17 - 22) | 20 (17 - 23) | 20 (17 - 23) | 19 (16 - 23) | 19 (17 - 23) | 19 (16 - 23) | 19 (16 - 22.5) | 19 (16 - 22) | 19 (16 - 22) |
| p=0.85 | Mean (se) | 20.2 (0.03) | 20.0 (0.03) | 19.9 (0.03) | 19.7 (0.04) | 19.7 (0.04) | 19.4 (0.04) | 19.4 (0.04) | 19.3 (0.04) | 19.1 (0.04) | 19.2 (0.04) | 19.1 (0.04) |
|  | Median  (p1 – p99) | 20 (18 - 23) | 20 (18 - 22) | 20 (18 - 23) | 20 (17 - 23) | 20 (17 - 23) | 19 (16 - 23) | 19 (16 - 23) | 19 (16 - 23) | 19 (16 - 22.5) | 19 (16 - 23) | 19 (16 - 22) |
| p=0.9 | Mean (se) | 20.1 (0.03) | 20.0 (0.02) | 19.9 (0.03) | 19.8 (0.04) | 19.6 (0.04) | 19.6 (0.04) | 19.5 (0.04) | 19.3 (0.05) | 19.2 (0.05) | 19.2 (0.04) | 19.1 (0.04) |
|  | Median  (p1 – p99) | 20 (18 - 22) | 20 (18 - 22) | 20 (17 - 22) | 20 (17 - 23) | 20 (17 - 23) | 20 (16 - 23) | 19 (16 - 23) | 19 (16 - 23) | 19 (16 - 23) | 19 (16 - 22.5) | 19 (16 - 22) |
| p=0.95 | Mean (se) | 20.1 (0.02) | 20.0 (0.02) | 19.9 (0.03) | 19.7 (0.04) | 19.6 (0.04) | 19.5 (0.04) | 19.4 (0.04) | 19.2 (0.04) | 19.2 (0.04) | 19.2 (0.04) | 19.0 (0.05) |
|  | Median  (p1 – p99) | 20 (19 - 22) | 20 (18 - 22) | 20 (17 - 22) | 20 (17 - 23) | 20 (17 - 23) | 19 (16 - 23) | 19 (16 - 23) | 19 (16 - 23) | 19 (16 - 22) | 19 (16 - 22) | 19 (16 - 23) |

Table 1c: Treatment balancing properties of 1:2:3 sequence balance minimisation with 0 (treatment totals only) to 10 factors with 2 levels, treatment totals weighted as total number of minimisation factors, the number of participants allocated to the treatment with smaller-allocation ratio, p = 0.95 to 0.5. Summary statistics from 1000 simulations. Treatment balance under best case scenario: mean (se): 5 (0), 10 (0), 20 (0) and median (p1 – p99): 5 (5 - 5), 10 (10 – 10), 20 (20 – 20) for sample size 30, 60 and 120 respectively.

| Random element |  | No. of factors | | | | | | | | | |
| --- | --- | --- | --- | --- | --- | --- | --- | --- | --- | --- | --- |
|  |  | 1 | 2 | 3 | 4 | 5 | 6 | 7 | 8 | 9 | 10 |
| Sample size 30, the expected number of participants allocated to the treatment with smallest-allocation ratio (n = 5) | | | | | | | | | | | |
| p=0.5 | Mean (se) | 5.0 (0.02) | 5.0 (0.01) | 4.9 (0.02) | 5.0 (0.02) | 5.0 (0.02) | 4.9 (0.02) | 4.9 (0.02) | 4.9 (0.02) | 4.9 (0.02) | 4.9 (0.02) |
|  | Median  (p1 – p99) | 5 (4 - 7) | 5 (4 - 6) | 5 (4 - 6) | 5 (4 - 6) | 5 (4 - 6) | 5 (4 - 6) | 5 (4 - 6) | 5 (4 - 6) | 5 (4 - 6) | 5 (3 - 6) |
| p=0.6 | Mean (se) | 5.0 (0.02) | 5.0 (0.02) | 4.9 (0.02) | 4.9 (0.02) | 4.9 (0.02) | 4.9 (0.02) | 4.9 (0.02) | 4.9 (0.02) | 4.9 (0.02) | 4.9 (0.02) |
|  | Median  (p1 – p99) | 5 (3.5 - 7) | 5 (4 - 6) | 5 (4 - 6) | 5 (4 - 6) | 5 (4 - 6) | 5 (4 - 6) | 5 (4 - 6) | 5 (4 - 6) | 5 (3 - 6) | 5 (4 - 6) |
| p=0.7 | Mean (se) | 5.0 (0.02) | 5.0 (0.01) | 4.9 (0.02) | 4.9 (0.02) | 4.9 (0.02) | 4.9 (0.02) | 4.9 (0.02) | 4.9 (0.02) | 4.9 (0.02) | 4.8 (0.02) |
|  | Median  (p1 – p99) | 5 (4 - 7) | 5 (4 - 6) | 5 (4 - 6) | 5 (4 - 6) | 5 (4 - 6) | 5 (4 - 6) | 5 (4 - 6) | 5 (4 - 6) | 5 (4 - 6) | 5 (4 - 6) |
| p=0.8 | Mean (se) | 5.0 (0.02) | 5.0 (0.01) | 4.9 (0.02) | 4.9 (0.02) | 5.0 (0.02) | 4.9 (0.02) | 4.9 (0.02) | 4.8 (0.02) | 4.9 (0.02) | 4.9 (0.02) |
|  | Median  (p1 – p99) | 5 (4 - 6) | 5 (4 - 6) | 5 (4 - 6) | 5 (4 - 6) | 5 (4 - 6) | 5 (4 - 6) | 5 (4 - 6) | 5 (4 - 6) | 5 (4 - 6) | 5 (3.5 - 6) |
| p=0.85 | Mean (se) | 5.0 (0.01) | 5.0 (0.01) | 5.0 (0.02) | 5.0 (0.02) | 4.9 (0.02) | 4.9 (0.02) | 4.9 (0.02) | 4.9 (0.02) | 4.9 (0.02) | 4.9 (0.02) |
|  | Median  (p1 – p99) | 5 (4 - 6) | 5 (4 - 6) | 5 (4 - 6) | 5 (4 - 6) | 5 (4 - 6) | 5 (4 - 6) | 5 (4 - 6) | 5 (4 - 6) | 5 (4 - 6) | 5 (3 - 6) |
| p=0.9 | Mean (se) | 5.0 (0.01) | 5.0 (0.01) | 4.9 (0.02) | 4.9 (0.02) | 5.0 (0.02) | 4.9 (0.02) | 4.9 (0.02) | 4.9 (0.02) | 4.9 (0.02) | 4.9 (0.02) |
|  | Median  (p1 – p99) | 5 (4 - 6) | 5 (4 - 6) | 5 (4 - 6) | 5 (4 - 6) | 5 (4 - 6) | 5 (4 - 6) | 5 (4 - 6) | 5 (3 - 6) | 5 (4 - 6) | 5 (4 - 6) |
| p=0.95 | Mean (se) | 5.0 (0.01) | 5.0 (0.01) | 4.9 (0.02) | 4.9 (0.02) | 5.0 (0.02) | 4.9 (0.02) | 4.9 (0.02) | 4.8 (0.02) | 4.9 (0.02) | 4.9 (0.02) |
|  | Median  (p1 – p99) | 5 (4 - 6) | 5 (4 - 6) | 5 (4 - 6) | 5 (4 - 6) | 5 (4 - 6) | 5 (4 - 6) | 5 (4 - 6) | 5 (3 - 6) | 5 (4 - 6) | 5 (4 - 6) |
| Sample size 60, the expected number of participants allocated to the treatment with smallest-allocation ratio (n = 10) | | | | | | | | | | | |
| p=0.5 | Mean (se) | 10.2 (0.03) | 10.0 (0.02) | 9.9 (0.03) | 9.9 (0.03) | 9.8 (0.03) | 9.8 (0.03) | 9.7 (0.03) | 9.7 (0.03) | 9.7 (0.03) | 9.7 (0.03) |
|  | Median  (p1 – p99) | 10 (8 - 12) | 10 (8 - 12) | 10 (8 - 12) | 10 (8 - 12) | 10 (8 - 12) | 10 (8 - 12) | 10 (8 - 12) | 10 (8 - 12) | 10 (8 - 12) | 10 (8 - 12) |
| p=0.6 | Mean (se) | 10.1 (0.03) | 10.0 (0.02) | 9.9 (0.03) | 9.9 (0.03) | 9.8 (0.03) | 9.7 (0.03) | 9.7 (0.03) | 9.7 (0.03) | 9.7 (0.03) | 9.6 (0.03) |
|  | Median  (p1 – p99) | 10 (8 - 12) | 10 (8 - 12) | 10 (8 - 12) | 10 (8 - 12) | 10 (8 - 12) | 10 (8 - 12) | 10 (8 - 12) | 10 (8 - 12) | 10 (8 - 12) | 10 (8 - 11) |
| p=0.7 | Mean (se) | 10.1 (0.02) | 10.0 (0.02) | 9.9 (0.02) | 9.8 (0.03) | 9.8 (0.03) | 9.8 (0.03) | 9.7 (0.03) | 9.7 (0.03) | 9.7 (0.03) | 9.6 (0.03) |
|  | Median  (p1 – p99) | 10 (8 - 12) | 10 (8 - 12) | 10 (8 - 12) | 10 (8 - 12) | 10 (8 - 12) | 10 (8 - 12) | 10 (8 - 12) | 10 (8 - 12) | 10 (8 - 12) | 10 (8 - 12) |
| p=0.8 | Mean (se) | 10.1 (0.02) | 10.0 (0.02) | 9.9 (0.03) | 9.8 (0.03) | 9.8 (0.03) | 9.7 (0.03) | 9.7 (0.03) | 9.7 (0.03) | 9.7 (0.03) | 9.7 (0.03) |
|  | Median  (p1 – p99) | 10 (9 - 12) | 10 (8 - 12) | 10 (8 - 12) | 10 (8 - 12) | 10 (8 - 12) | 10 (8 - 12) | 10 (8 - 12) | 10 (8 - 12) | 10 (8 - 12) | 10 (8 - 12) |
| p=0.85 | Mean (se) | 10.1 (0.02) | 10.0 (0.02) | 9.9 (0.03) | 9.8 (0.03) | 9.8 (0.03) | 9.7 (0.03) | 9.7 (0.03) | 9.7 (0.03) | 9.7 (0.03) | 9.6 (0.03) |
|  | Median  (p1 – p99) | 10 (9 - 12) | 10 (8.5 - 12) | 10 (8 - 12) | 10 (8 - 12) | 10 (8 - 12) | 10 (8 - 12) | 10 (8 - 12) | 10 (8 - 12) | 10 (8 - 12) | 10 (8 - 12) |
| p=0.9 | Mean (se) | 10.0 (0.02) | 10.0 (0.02) | 10.0 (0.02) | 9.9 (0.03) | 9.8 (0.03) | 9.8 (0.03) | 9.7 (0.03) | 9.7 (0.03) | 9.7 (0.03) | 9.7 (0.03) |
|  | Median  (p1 – p99) | 10 (9 - 11) | 10 (8 - 12) | 10 (8 - 12) | 10 (8 - 12) | 10 (8 - 12) | 10 (8 - 12) | 10 (8 - 12) | 10 (8 - 12) | 10 (8 - 12) | 10 (8 - 12) |
| p=0.95 | Mean (se) | 10.0 (0.02) | 10.0 (0.02) | 9.9 (0.02) | 9.8 (0.03) | 9.8 (0.03) | 9.8 (0.03) | 9.7 (0.03) | 9.7 (0.03) | 9.7 (0.03) | 9.7 (0.03) |
|  | Median  (p1 – p99) | 10 (9 - 11) | 10 (8.5 - 12) | 10 (8 - 12) | 10 (8 - 12) | 10 (8 - 12) | 10 (8 - 12) | 10 (8 - 12) | 10 (8 - 12) | 10 (8 - 12) | 10 (8 - 12) |
| Sample size 120, the expected number of participants allocated to the treatment with smallest-allocation ratio (n = 20) | | | | | | | | | | | |
| p=0.5 | Mean (se) | 20.2 (0.03) | 19.9 (0.03) | 19.8 (0.03) | 19.7 (0.04) | 19.7 (0.04) | 19.6 (0.04) | 19.4 (0.04) | 19.4 (0.04) | 19.4 (0.04) | 19.4 (0.04) |
|  | Median  (p1 – p99) | 20 (18 - 23) | 20 (17 - 23) | 20 (17 - 22) | 20 (17 - 23) | 20 (17 - 23) | 20 (16.5 - 23) | 19 (17 - 22) | 19 (16 - 22) | 19 (16 - 22) | 19 (16 - 22) |
| p=0.6 | Mean (se) | 20.1 (0.03) | 19.9 (0.03) | 19.9 (0.04) | 19.7 (0.03) | 19.6 (0.04) | 19.6 (0.04) | 19.4 (0.04) | 19.4 (0.04) | 19.3 (0.04) | 19.3 (0.04) |
|  | Median  (p1 – p99) | 20 (18 - 23) | 20 (17 - 22) | 20 (17 - 23) | 20 (17 - 23) | 20 (17 - 23) | 20 (17 - 22) | 19 (17 - 22) | 19 (16 - 22) | 19 (16 - 22) | 19 (16 - 22) |
| p=0.7 | Mean (se) | 20.1 (0.03) | 20.0 (0.03) | 19.8 (0.04) | 19.7 (0.03) | 19.7 (0.04) | 19.6 (0.04) | 19.4 (0.04) | 19.4 (0.04) | 19.3 (0.04) | 19.3 (0.04) |
|  | Median  (p1 – p99) | 20 (18 - 22) | 20 (18 - 22) | 20 (17 - 23) | 20 (17 - 22) | 20 (17 - 23) | 20 (17 - 22) | 19 (17 - 22) | 19 (16 - 22) | 19 (16 - 22) | 19 (16.5 - 22) |
| p=0.8 | Mean (se) | 20.0 (0.03) | 20.0 (0.03) | 19.8 (0.04) | 19.8 (0.04) | 19.7 (0.04) | 19.6 (0.04) | 19.5 (0.04) | 19.4 (0.04) | 19.3 (0.04) | 19.3 (0.04) |
|  | Median  (p1 – p99) | 20 (18 - 22) | 20 (18 - 22) | 20 (17 - 23) | 20 (17 - 23) | 20 (17 - 23) | 20 (16.5 - 22) | 20 (17 - 22) | 19 (17 - 22) | 19 (16 - 22) | 19 (16 - 22) |
| p=0.85 | Mean (se) | 20.0 (0.03) | 19.9 (0.03) | 19.8 (0.04) | 19.7 (0.04) | 19.6 (0.04) | 19.5 (0.04) | 19.4 (0.04) | 19.4 (0.04) | 19.5 (0.04) | 19.3 (0.04) |
|  | Median  (p1 – p99) | 20 (18 - 22) | 20 (17 - 22) | 20 (17 - 23) | 20 (17 - 22) | 20 (17 - 22) | 20 (17 - 22) | 19 (17 - 22) | 19 (17 - 22) | 19 (17 - 22) | 19 (16 - 22) |
| p=0.9 | Mean (se) | 20.0 (0.02) | 19.9 (0.03) | 19.8 (0.04) | 19.7 (0.04) | 19.6 (0.04) | 19.6 (0.04) | 19.4 (0.04) | 19.5 (0.04) | 19.3 (0.04) | 19.3 (0.04) |
|  | Median  (p1 – p99) | 20 (18 - 22) | 20 (18 - 23) | 20 (17 - 23) | 20 (17 - 22) | 20 (17 - 22) | 20 (16.5 - 23) | 19 (17 - 22) | 19.5 (16 - 22) | 19 (16 - 22) | 19 (16 - 22.5) |
| p=0.95 | Mean (se) | 20.0 (0.02) | 20.0 (0.03) | 19.8 (0.03) | 19.7 (0.04) | 19.6 (0.04) | 19.6 (0.04) | 19.5 (0.04) | 19.4 (0.04) | 19.3 (0.04) | 19.3 (0.04) |
|  | Median  (p1 – p99) | 20 (18 - 22) | 20 (18 - 22) | 20 (17 - 22) | 20 (17 - 22) | 20 (17 - 22) | 20 (17 - 22) | 20 (17 - 22.5) | 19 (17 - 22) | 19 (16 - 22) | 19 (16 - 22) |

**Appendix B**

Table 2a: Factor balancing properties of 1:2:3 sequence balance minimisation with 1 to 10 factors with 2 levels, treatment totals weighted as zero, number of subjects allocated to the treatment with smallest-allocation ratio, random element = 0.95 to 0.5. Summary statistics from 1000 simulations.

| Random element |  | Treatment totals only (worst case scenario) | No. of factors | | | | | | | | | |
| --- | --- | --- | --- | --- | --- | --- | --- | --- | --- | --- | --- | --- |
|  |  |  | 1 | 2 | 3 | 4 | 5 | 6 | 7 | 8 | 9 | 10 |
| Sample size 30, the expected number of participants allocated with the particular factor level n = 2.5 | | | | | | | | | | | | |
| p=0.5 | Mean (se) | 2.7 (0.03) | 2.4 (0.03) | 2.4 (0.02) | 2.3 (0.02) | 2.3 (0.03) | 2.4 (0.03) | 2.3 (0.03) | 2.3 (0.03) | 2.4 (0.03) | 2.3 (0.03) | 2.3 (0.03) |
|  | Median  (p1 – p99) | 3 (1 - 6) | 2 (1 - 4) | 2 (1 - 4) | 2 (1 - 4) | 2 (1 - 5) | 2 (1 - 4) | 2 (1 - 4) | 2 (1 - 4) | 2 (1 - 4) | 2 (1 - 4) | 2 (1 - 4) |
| p=0.6 | Mean (se) | 2.6 (0.03) | 2.4 (0.02) | 2.4 (0.02) | 2.3 (0.02) | 2.3 (0.03) | 2.3 (0.03) | 2.3 (0.03) | 2.3 (0.03) | 2.4 (0.03) | 2.3 (0.03) | 2.3 (0.03) |
|  | Median  (p1 – p99) | 3 (1 - 5) | 2 (1 - 4) | 2 (1 - 4) | 2 (1 - 4) | 2 (1 - 4) | 2 (1 - 4) | 2 (1 - 4) | 2 (1 - 5) | 2 (1 - 4) | 2 (1 - 5) | 2 (1 - 4.5) |
| p=0.7 | Mean (se) | 2.6 (0.03) | 2.5 (0.02) | 2.4 (0.02) | 2.3 (0.02) | 2.3 (0.03) | 2.3 (0.03) | 2.3 (0.03) | 2.3 (0.03) | 2.4 (0.03) | 2.4 (0.03) | 2.4 (0.03) |
|  | Median  (p1 – p99) | 3 (1 - 5) | 2 (1 - 4) | 2 (1 - 4) | 2 (1 - 4) | 2 (1 - 4) | 2 (1 - 4) | 2 (1 - 4) | 2 (1 - 4) | 2 (1 - 4) | 2 (1 - 4) | 2 (1 - 5) |
| p=0.8 | Mean (se) | 2.5 (0.03) | 2.4 (0.02) | 2.4 (0.02) | 2.3 (0.02) | 2.3 (0.03) | 2.4 (0.03) | 2.3 (0.03) | 2.3 (0.03) | 2.4 (0.03) | 2.3 (0.03) | 2.3 (0.03) |
|  | Median  (p1 – p99) | 2.5 (1 - 5) | 2 (1 - 4) | 2 (1 - 4) | 2 (1 - 4) | 2 (1 - 4) | 2 (1 - 4) | 2 (1 - 4) | 2 (1 - 4) | 2 (1 - 4) | 2 (1 - 5) | 2 (1 - 4) |
| p=0.85 | Mean (se) | 2.6 (0.03) | 2.4 (0.02) | 2.4 (0.02) | 2.3 (0.02) | 2.3 (0.03) | 2.4 (0.03) | 2.3 (0.03) | 2.3 (0.03) | 2.3 (0.03) | 2.3 (0.03) | 2.3 (0.03) |
|  | Median  (p1 – p99) | 3 (1 - 5) | 2 (1 - 4) | 2 (1 - 4) | 2 (1 - 4) | 2 (1 - 4) | 2 (1 - 5) | 2 (1 - 4) | 2 (1 - 5) | 2 (1 - 4) | 2 (1 - 4) | 2 (1 - 4) |
| p=0.9 | Mean (se) | 2.5 (0.03) | 2.4 (0.02) | 2.3 (0.02) | 2.3 (0.02) | 2.3 (0.03) | 2.4 (0.03) | 2.3 (0.03) | 2.3 (0.03) | 2.4 (0.03) | 2.4 (0.03) | 2.3 (0.03) |
|  | Median  (p1 – p99) | 2 (1 - 5) | 2 (1 - 4) | 2 (1 - 4) | 2 (1 - 4) | 2 (1 - 5) | 2 (1 - 4) | 2 (1 - 4) | 2 (1 - 5) | 2 (1 - 5) | 2 (1 - 4) | 2 (1 - 4) |
| p=0.95 | Mean (se) | 2.5 (0.03) | 2.4 (0.02) | 2.3 (0.02) | 2.3 (0.02) | 2.3 (0.03) | 2.4 (0.03) | 2.4 (0.03) | 2.3 (0.03) | 2.4 (0.03) | 2.3 (0.03) | 2.3 (0.03) |
|  | Median  (p1 – p99) | 2 (1 - 5) | 2 (1 - 4) | 2 (1 - 4) | 2 (1 - 4) | 2 (1 - 5) | 2 (1 - 4) | 2 (1 - 5) | 2 (1 - 5) | 2 (1 - 5) | 2 (1 - 4) | 2 (1 - 4) |
| Sample size 60, the expected number of participants allocated with the particular factor level n = 5 | | | | | | | | | | | | |
| p=0.5 | Mean (se) | 5.2 (0.05) | 5.1 (0.03) | 5.1 (0.02) | 5.0 (0.03) | 4.9 (0.03) | 4.8 (0.04) | 4.8 (0.04) | 4.8 (0.04) | 4.7 (0.03) | 4.7 (0.04) | 4.8 (0.04) |
|  | Median  (p1 – p99) | 5 (2 - 9) | 5 (3 - 7) | 5 (4 - 7) | 5 (3 - 7) | 5 (2 - 7) | 5 (2 - 8) | 5 (2 - 8) | 5 (2 - 8) | 5 (2 - 7) | 5 (2 - 7) | 5 (2 - 7) |
| p=0.6 | Mean (se) | 5.1 (0.05) | 5.1 (0.03) | 5.1 (0.03) | 5.0 (0.03) | 4.9 (0.03) | 4.8 (0.03) | 4.8 (0.04) | 4.8 (0.04) | 4.7 (0.04) | 4.7 (0.03) | 4.7 (0.04) |
|  | Median  (p1 – p99) | 5 (2 - 9) | 5 (3 - 7) | 5 (3 - 7) | 5 (3 - 7) | 5 (3 - 7) | 5 (2 - 7) | 5 (2 - 8) | 5 (2 - 7) | 5 (2 - 7) | 5 (2 - 8) | 5 (2 - 8) |
| p=0.7 | Mean (se) | 5.1 (0.05) | 5.1 (0.02) | 5.0 (0.02) | 5.0 (0.03) | 4.8 (0.03) | 4.9 (0.04) | 4.7 (0.04) | 4.8 (0.04) | 4.8 (0.04) | 4.7 (0.04) | 4.8 (0.04) |
|  | Median  (p1 – p99) | 5 (2 - 9) | 5 (3 - 7) | 5 (3.5 - 7) | 5 (3 - 7) | 5 (2 - 7) | 5 (2 - 8) | 5 (2 - 8) | 5 (2 - 8) | 5 (2 - 8) | 5 (2 - 7) | 5 (2 - 7.5) |
| p=0.8 | Mean (se) | 5.1 (0.04) | 5.0 (0.02) | 5.0 (0.02) | 5.0 (0.03) | 4.9 (0.03) | 4.8 (0.04) | 4.8 (0.04) | 4.8 (0.04) | 4.7 (0.04) | 4.7 (0.04) | 4.7 (0.04) |
|  | Median  (p1 – p99) | 5 (2 - 8.5) | 5 (4 - 6.5) | 5 (4 - 7) | 5 (3 - 7) | 5 (2 - 7) | 5 (2.5 - 8) | 5 (2 - 8) | 5 (2 - 8) | 5 (2 - 7.5) | 5 (2 - 7) | 5 (2 - 8) |
| p=0.85 | Mean (se) | 5.1 (0.04) | 5.0 (0.02) | 5.1 (0.02) | 5.0 (0.03) | 4.9 (0.03) | 4.7 (0.04) | 4.7 (0.03) | 4.9 (0.04) | 4.8 (0.03) | 4.8 (0.03) | 4.8 (0.04) |
|  | Median  (p1 – p99) | 5 (2 - 8) | 5 (4 - 6) | 5 (3.5 - 7) | 5 (3 - 7) | 5 (2 - 7) | 5 (2 - 7) | 5 (2 - 7) | 5 (2 - 8) | 5 (2 - 8) | 5 (2 - 7) | 5 (2 - 8) |
| p=0.9 | Mean (se) | 5.0 (0.04) | 5.0 (0.02) | 5.0 (0.02) | 5.0 (0.03) | 4.8 (0.03) | 4.8 (0.03) | 4.8 (0.04) | 4.8 (0.04) | 4.7 (0.04) | 4.7 (0.04) | 4.8 (0.04) |
|  | Median  (p1 – p99) | 5 (2 - 8) | 5 (4 - 6) | 5 (3 - 7) | 5 (3 - 7) | 5 (2 - 7) | 5 (2 - 7) | 5 (2 - 8) | 5 (2 - 7.5) | 5 (2 - 8) | 5 (2 - 8) | 5 (2 - 8) |
| p=0.95 | Mean (se) | 4.9 (0.04) | 5.0 (0.01) | 5.1 (0.02) | 5.0 (0.03) | 4.8 (0.03) | 4.8 (0.04) | 4.8 (0.04) | 4.8 (0.04) | 4.7 (0.03) | 4.8 (0.04) | 4.7 (0.04) |
|  | Median  (p1 – p99) | 5 (2 - 8) | 5 (4 - 6) | 5 (3 - 7) | 5 (3 - 7) | 5 (2 - 7) | 5 (2 - 8) | 5 (2 - 8) | 5 (2 - 8) | 5 (2 - 7) | 5 (2 - 7) | 5 (2 - 7) |
| Sample size 120, the expected number of participants allocated with the particular factor level n = 10 | | | | | | | | | | | | |
| p=0.5 | Mean (se) | 10.5 (0.08) | 10.0 (0.04) | 10.0 (0.04) | 10.0 (0.04) | 10.2 (0.05) | 9.9 (0.05) | 9.9 (0.06) | 9.7 (0.06) | 9.7 (0.06) | 9.7 (0.06) | 9.7 (0.06) |
|  | Median  (p1 – p99) | 10 (5 - 16.5) | 10 (7 - 13) | 10 (7 - 13) | 10 (7 - 13) | 10 (6.5 - 14) | 10 (6 - 14) | 10 (5.5 - 14) | 10 (5 - 15) | 10 (5 - 14) | 10 (6 - 14) | 10 (5 - 14) |
| p=0.6 | Mean (se) | 10.3 (0.07) | 10.0 (0.04) | 10.1 (0.04) | 10.0 (0.04) | 10.1 (0.05) | 10.0 (0.05) | 9.9 (0.06) | 9.8 (0.06) | 9.7 (0.06) | 9.6 (0.06) | 9.8 (0.06) |
|  | Median  (p1 – p99) | 10 (5 - 15.5) | 10 (7 - 13) | 10 (7 - 13) | 10 (7 - 13) | 10 (7 - 14) | 10 (6 - 14) | 10 (6 - 14) | 10 (5 - 14) | 10 (5 - 14) | 10 (5 - 14) | 10 (5 - 14) |
| p=0.7 | Mean (se) | 10.2 (0.07) | 10.0 (0.03) | 10.0 (0.03) | 10.0 (0.04) | 10.1 (0.05) | 9.9 (0.05) | 9.9 (0.06) | 9.7 (0.06) | 9.9 (0.06) | 9.6 (0.06) | 9.8 (0.06) |
|  | Median  (p1 – p99) | 10 (5 - 15) | 10 (8 - 13) | 10 (7.5 - 13) | 10 (7 - 13) | 10 (7 - 14) | 10 (6 - 14) | 10 (6 - 14) | 10 (5 - 15) | 10 (5 - 14) | 10 (5 - 15) | 10 (6 - 15) |
| p=0.8 | Mean (se) | 10.2 (0.07) | 10.0 (0.03) | 10.1 (0.03) | 10.0 (0.04) | 10.1 (0.05) | 10.0 (0.06) | 9.9 (0.06) | 9.7 (0.06) | 9.7 (0.06) | 9.7 (0.06) | 9.7 (0.06) |
|  | Median  (p1 – p99) | 10 (5 - 16) | 10 (8 - 12) | 10 (7 - 13) | 10 (7 - 13) | 10 (7 - 13.5) | 10 (6 - 14.5) | 10 (6 - 14) | 10 (6 - 14) | 10 (6 - 15) | 10 (5.5 - 15) | 10 (5 - 14) |
| p=0.85 | Mean (se) | 10.1 (0.07) | 10.0 (0.03) | 10.0 (0.03) | 10.0 (0.04) | 10.1 (0.05) | 9.9 (0.05) | 9.9 (0.06) | 9.7 (0.06) | 9.5 (0.06) | 9.7 (0.06) | 9.7 (0.06) |
|  | Median  (p1 – p99) | 10 (5 - 15) | 10 (8 - 12) | 10 (8 - 13) | 10 (7 - 13) | 10 (7 - 13.5) | 10 (6 - 14) | 10 (5 - 14) | 10 (6 - 14) | 10 (5 - 14) | 10 (5 - 14) | 10 (6 - 14) |
| p=0.9 | Mean (se) | 10.1 (0.07) | 10.0 (0.02) | 10.1 (0.03) | 10.0 (0.04) | 10.0 (0.05) | 10.0 (0.05) | 10.0 (0.06) | 9.7 (0.06) | 9.7 (0.06) | 9.7 (0.06) | 9.7 (0.06) |
|  | Median  (p1 – p99) | 10 (5 - 15) | 10 (8 - 12) | 10 (7.5 - 13) | 10 (7 - 13) | 10 (6 - 14) | 10 (6 - 14) | 10 (6 - 14.5) | 10 (6 - 14) | 10 (5 - 14) | 10 (5 - 14) | 10 (5 - 14) |
| p=0.95 | Mean (se) | 10.1 (0.07) | 10.0 (0.01) | 10.1 (0.03) | 10.0 (0.04) | 10.1 (0.05) | 10.0 (0.05) | 9.9 (0.06) | 9.7 (0.06) | 9.6 (0.06) | 9.7 (0.06) | 9.8 (0.06) |
|  | Median  (p1 – p99) | 10 (5 - 15) | 10 (9 - 11) | 10 (7 - 13) | 10 (7 - 13) | 10 (6.5 - 14) | 10 (6 - 14) | 10 (6 - 14) | 10 (6 - 14) | 10 (6 - 14) | 10 (5 - 14) | 10 (5 - 14) |

Table 2b: Factor balancing properties of 1:2:3 sequence balance minimisation with 1 to 10 factors with 2 levels, treatment totals weighted as one, the number of participants allocated to the treatment with smallest-allocation ratio, random element = 0.95 to 0.5. Summary statistics from 1000 simulations.

| Random element |  | Treatment totals only (worst case scenario) | No. of factors | | | | | | | | | |
| --- | --- | --- | --- | --- | --- | --- | --- | --- | --- | --- | --- | --- |
|  |  |  | 1 | 2 | 3 | 4 | 5 | 6 | 7 | 8 | 9 | 10 |
| Sample size 30, the expected number of participants allocated with the particular factor level n = 2.5 | | | | | | | | | | | | |
| p=0.5 | Mean (se) | 2.7 (0.03) | 2.6 (0.02) | 2.5 (0.02) | 2.4 (0.02) | 2.4 (0.03) | 2.5 (0.03) | 2.5 (0.03) | 2.5 (0.03) | 2.5 (0.03) | 2.5 (0.03) | 2.4 (0.03) |
|  | Median  (p1 – p99) | 3 (1 - 6) | 3 (1 - 4) | 2 (1 - 4) | 2 (1 - 4) | 2 (1 - 4) | 3 (1 - 5) | 2 (1 - 4.5) | 2.5 (1 - 5) | 2 (1 - 5) | 2 (1 - 5) | 2 (1 - 5) |
| p=0.6 | Mean (se) | 2.6 (0.03) | 2.5 (0.02) | 2.5 (0.02) | 2.3 (0.03) | 2.4 (0.03) | 2.5 (0.03) | 2.5 (0.03) | 2.4 (0.03) | 2.5 (0.03) | 2.5 (0.03) | 2.4 (0.03) |
|  | Median  (p1 – p99) | 3 (1 - 5) | 3 (1 - 4) | 2 (1 - 4) | 2 (1 - 4) | 2 (1 - 4) | 2 (1 - 4) | 3 (1 - 5) | 2 (1 - 5) | 2 (1 - 4) | 2 (1 - 5) | 2 (1 - 5) |
| p=0.7 | Mean (se) | 2.6 (0.03) | 2.5 (0.02) | 2.5 (0.02) | 2.4 (0.02) | 2.4 (0.03) | 2.5 (0.03) | 2.5 (0.03) | 2.5 (0.03) | 2.5 (0.03) | 2.5 (0.03) | 2.4 (0.03) |
|  | Median  (p1 – p99) | 3 (1 - 5) | 3 (1 - 4) | 2 (1 - 4) | 2 (1 - 4) | 2 (1 - 4.5) | 2 (1 - 4) | 3 (1 - 4) | 2 (1 - 5) | 2 (1 - 5) | 2 (1 - 5) | 2 (1 - 5) |
| p=0.8 | Mean (se) | 2.5 (0.03) | 2.5 (0.02) | 2.4 (0.02) | 2.4 (0.03) | 2.4 (0.03) | 2.5 (0.03) | 2.5 (0.03) | 2.5 (0.03) | 2.5 (0.03) | 2.4 (0.03) | 2.4 (0.03) |
|  | Median  (p1 – p99) | 2.5 (1 - 5) | 2 (1 - 4) | 2 (1 - 4) | 2 (1 - 4) | 2 (1 - 5) | 3 (1 - 4) | 2 (1 - 4.5) | 2.5 (1 - 5) | 2 (1 - 4) | 2 (1 - 5) | 2 (1 - 4) |
| p=0.85 | Mean (se) | 2.6 (0.03) | 2.6 (0.02) | 2.4 (0.02) | 2.3 (0.02) | 2.5 (0.03) | 2.5 (0.03) | 2.5 (0.03) | 2.5 (0.03) | 2.5 (0.03) | 2.5 (0.03) | 2.4 (0.03) |
|  | Median  (p1 – p99) | 3 (1 - 5) | 3 (1 - 4) | 2 (1 - 4) | 2 (1 - 4) | 2 (1 - 4) | 2 (1 - 4) | 3 (1 - 4) | 2 (1 - 5) | 3 (1 - 5) | 2 (1 - 4) | 2 (1 - 5) |
| p=0.9 | Mean (se) | 2.5 (0.03) | 2.5 (0.02) | 2.4 (0.02) | 2.3 (0.03) | 2.4 (0.03) | 2.5 (0.03) | 2.5 (0.03) | 2.5 (0.03) | 2.5 (0.03) | 2.5 (0.03) | 2.5 (0.03) |
|  | Median  (p1 – p99) | 2 (1 - 5) | 2 (1 - 4) | 2 (1 - 4) | 2 (1 - 4) | 2 (1 - 4) | 3 (1 - 5) | 2 (1 - 5) | 2 (1 - 5) | 2 (1 - 4) | 2 (1 - 5) | 2 (1 - 5) |
| p=0.95 | Mean (se) | 2.5 (0.03) | 2.5 (0.02) | 2.4 (0.02) | 2.4 (0.02) | 2.4 (0.03) | 2.5 (0.03) | 2.5 (0.03) | 2.5 (0.03) | 2.5 (0.03) | 2.5 (0.03) | 2.4 (0.03) |
|  | Median  (p1 – p99) | 2 (1 - 5) | 2 (1 - 4) | 2 (1 - 4) | 2 (1 - 4) | 2 (1 - 4) | 2 (1 - 4) | 3 (1 - 5) | 2 (1 - 5) | 2 (1 - 5) | 2 (1 - 5) | 2 (1 - 5) |
| Sample size 60, the expected number of participants allocated with the particular factor level n = 5 | | | | | | | | | | | | |
| p=0.5 | Mean (se) | 5.2 (0.05) | 5.1 (0.02) | 5.0 (0.03) | 5.0 (0.03) | 4.8 (0.03) | 4.7 (0.04) | 4.7 (0.03) | 4.8 (0.04) | 4.8 (0.04) | 4.7 (0.04) | 4.8 (0.04) |
|  | Median  (p1 – p99) | 5 (2 - 9) | 5 (3 - 7) | 5 (3 - 7) | 5 (3 - 7) | 5 (2 - 7) | 5 (2 - 7.5) | 5 (2 - 7.5) | 5 (2 - 8) | 5 (2 - 7) | 5 (2 - 8) | 5 (2 - 8) |
| p=0.6 | Mean (se) | 5.1 (0.05) | 5.1 (0.02) | 5.0 (0.03) | 4.9 (0.03) | 4.9 (0.04) | 4.7 (0.04) | 4.7 (0.03) | 4.8 (0.04) | 4.8 (0.03) | 4.7 (0.04) | 4.8 (0.04) |
|  | Median  (p1 – p99) | 5 (2 - 9) | 5 (4 - 7) | 5 (3 - 7) | 5 (3 - 7) | 5 (2 - 8) | 5 (2 - 8) | 5 (2 - 8) | 5 (2 - 8) | 5 (2 - 7) | 5 (2 - 7) | 5 (2 - 7) |
| p=0.7 | Mean (se) | 5.1 (0.05) | 5.0 (0.02) | 5.0 (0.03) | 5.0 (0.03) | 4.8 (0.04) | 4.7 (0.04) | 4.7 (0.04) | 4.7 (0.04) | 4.7 (0.04) | 4.7 (0.04) | 4.8 (0.04) |
|  | Median  (p1 – p99) | 5 (2 - 9) | 5 (4 - 6.5) | 5 (3 - 7) | 5 (3 - 7) | 5 (2 - 7) | 5 (2 - 8) | 5 (2 - 8) | 5 (2 - 8) | 5 (2 - 8) | 5 (2 - 8) | 5 (2 - 8) |
| p=0.8 | Mean (se) | 5.1 (0.04) | 5.0 (0.02) | 5.0 (0.02) | 5.0 (0.03) | 4.8 (0.03) | 4.8 (0.04) | 4.8 (0.04) | 4.9 (0.04) | 4.7 (0.04) | 4.8 (0.04) | 4.8 (0.04) |
|  | Median  (p1 – p99) | 5 (2 - 8.5) | 5 (4 - 6) | 5 (3 - 7) | 5 (3 - 7) | 5 (2 - 7) | 5 (2 - 8) | 5 (2 - 7) | 5 (2 - 8) | 5 (2 - 8) | 5 (2 - 8) | 5 (2 - 8) |
| p=0.85 | Mean (se) | 5.1 (0.04) | 5.0 (0.02) | 5.0 (0.03) | 5.0 (0.03) | 4.8 (0.03) | 4.8 (0.04) | 4.8 (0.04) | 4.7 (0.04) | 4.8 (0.03) | 4.8 (0.04) | 4.8 (0.04) |
|  | Median  (p1 – p99) | 5 (2 - 8) | 5 (4 - 6) | 5 (3 - 7) | 5 (3 - 7) | 5 (2 - 7) | 5 (2 - 8) | 5 (2 - 8) | 5 (2 - 7) | 5 (2 - 7) | 5 (2 - 8) | 5 (2 - 8) |
| p=0.9 | Mean (se) | 5.0 (0.04) | 5.0 (0.02) | 5.0 (0.03) | 5.0 (0.03) | 4.8 (0.04) | 4.7 (0.04) | 4.8 (0.04) | 4.8 (0.04) | 4.8 (0.03) | 4.7 (0.04) | 4.8 (0.04) |
|  | Median  (p1 – p99) | 5 (2 - 8) | 5 (4 - 6) | 5 (3 - 7) | 5 (3 - 7) | 5 (2 - 7) | 5 (2 - 8) | 5 (2 - 7) | 5 (2 - 8) | 5 (2 - 7) | 5 (2 - 8) | 5 (2 - 8) |
| p=0.95 | Mean (se) | 4.9 (0.04) | 5.0 (0.01) | 5.0 (0.03) | 5.0 (0.03) | 4.7 (0.03) | 4.7 (0.04) | 4.7 (0.04) | 4.8 (0.04) | 4.8 (0.04) | 4.7 (0.04) | 4.8 (0.04) |
|  | Median  (p1 – p99) | 5 (2 - 8) | 5 (4 - 6) | 5 (3 - 7) | 5 (3 - 7) | 5 (2 - 7) | 5 (2 - 8) | 5 (2 - 7.5) | 5 (2 - 7) | 5 (2 - 7) | 5 (2 - 7) | 5 (2 - 7) |
| Sample size 120, the expected number of participants allocated with the particular factor level n = 10 | | | | | | | | | | | | |
| p=0.5 | Mean (se) | 10.5 (0.08) | 10.1 (0.03) | 10.0 (0.04) | 10.1 (0.05) | 10.1 (0.05) | 10.1 (0.05) | 9.9 (0.06) | 9.7 (0.06) | 9.6 (0.06) | 9.6 (0.06) | 9.7 (0.06) |
|  | Median  (p1 – p99) | 10 (5 - 16.5) | 10 (8 - 12) | 10 (7 - 13) | 10 (7 - 13.5) | 10 (6 - 14) | 10 (6 - 14) | 10 (6 - 14) | 10 (6 - 14) | 10 (5 - 14) | 10 (5 - 14) | 10 (5 - 15) |
| p=0.6 | Mean (se) | 10.3 (0.07) | 10.1 (0.03) | 10.0 (0.04) | 10.0 (0.04) | 10.0 (0.05) | 10.0 (0.05) | 9.9 (0.06) | 9.8 (0.06) | 9.8 (0.07) | 9.8 (0.06) | 9.7 (0.06) |
|  | Median  (p1 – p99) | 10 (5 - 15.5) | 10 (8 - 12) | 10 (7 - 13) | 10 (7 - 13) | 10 (6 - 14) | 10 (6 - 14) | 10 (6 - 14) | 10 (5 - 14) | 10 (5 - 14) | 10 (5 - 14) | 10 (5 - 14) |
| p=0.7 | Mean (se) | 10.2 (0.07) | 10.1 (0.02) | 9.9 (0.04) | 10.1 (0.05) | 10.0 (0.05) | 9.9 (0.06) | 9.9 (0.06) | 9.6 (0.06) | 9.8 (0.06) | 9.7 (0.06) | 9.7 (0.06) |
|  | Median  (p1 – p99) | 10 (5 - 15) | 10 (8 - 12) | 10 (7 - 13) | 10 (7 - 14) | 10 (6 - 14) | 10 (6 - 14) | 10 (6 - 14) | 10 (5 - 15) | 10 (6 - 14.5) | 10 (5 - 15) | 10 (6 - 15) |
| p=0.8 | Mean (se) | 10.2 (0.07) | 10.0 (0.02) | 9.9 (0.04) | 10.1 (0.05) | 10.1 (0.05) | 9.9 (0.05) | 9.8 (0.06) | 9.7 (0.06) | 9.7 (0.06) | 9.7 (0.06) | 9.7 (0.06) |
|  | Median  (p1 – p99) | 10 (5 - 16) | 10 (8 - 12) | 10 (7 - 13) | 10 (7 - 14) | 10 (6 - 14) | 10 (6 - 14) | 10 (6 - 14) | 10 (5 - 14) | 10 (6 - 14) | 10 (5 - 14) | 10 (5 - 15) |
| p=0.85 | Mean (se) | 10.1 (0.07) | 10.0 (0.02) | 10.0 (0.04) | 10.0 (0.05) | 10.1 (0.05) | 10.1 (0.05) | 9.9 (0.06) | 9.7 (0.06) | 9.6 (0.06) | 9.7 (0.06) | 9.9 (0.06) |
|  | Median  (p1 – p99) | 10 (5 - 15) | 10 (8 - 12) | 10 (7 - 13) | 10 (6 - 13) | 10 (6 - 14) | 10 (6 - 14) | 10 (6 - 14) | 10 (5 - 14) | 10 (5 - 14) | 10 (6 - 14) | 10 (6 - 14) |
| p=0.9 | Mean (se) | 10.1 (0.07) | 10.0 (0.02) | 9.9 (0.04) | 10.0 (0.04) | 10.0 (0.05) | 9.9 (0.06) | 9.9 (0.06) | 9.7 (0.06) | 9.7 (0.06) | 9.8 (0.06) | 9.8 (0.06) |
|  | Median  (p1 – p99) | 10 (5 - 15) | 10 (8 - 12) | 10 (7 - 13) | 10 (7 - 14) | 10 (6 - 14) | 10 (6 - 14) | 10 (5.5 - 14) | 10 (5 - 15) | 10 (5.5 - 14) | 10 (5 - 14.5) | 10 (5 - 14) |
| p=0.95 | Mean (se) | 10.1 (0.07) | 10.0 (0.02) | 10.0 (0.04) | 10.0 (0.05) | 10.1 (0.05) | 10.0 (0.06) | 9.9 (0.06) | 9.7 (0.06) | 9.7 (0.06) | 9.8 (0.06) | 9.7 (0.06) |
|  | Median  (p1 – p99) | 10 (5 - 15) | 10 (8 - 12) | 10 (7 - 13) | 10 (7 - 13) | 10 (7 - 14) | 10 (6 - 14) | 10 (6 - 14) | 10 (5 - 14) | 10 (5 - 14.5) | 10 (6 - 15) | 10 (5 - 14) |

Table 2c: Factor balancing properties of 1:2:3 sequence balance minimisation with 1 to 10 factors with 2 levels, treatment totals weighted as total number of minimisation factors, the number of participants allocated to the treatment with smallest-allocation ratio, random element = 0.95 to 0.5. Summary statistics from 1000 simulations.

| Random element |  | Treatment totals only (worst case scenario) | No. of factors | | | | | | | | | |
| --- | --- | --- | --- | --- | --- | --- | --- | --- | --- | --- | --- | --- |
|  |  |  | 1 | 2 | 3 | 4 | 5 | 6 | 7 | 8 | 9 | 10 |
| Sample size 30, the expected number of participants allocated with the particular factor level n = 2.5 | | | | | | | | | | | | |
| p=0.5 | Mean (se) | 2.7 (0.03) | 2.6 (0.02) | 2.4 (0.02) | 2.4 (0.03) | 2.4 (0.03) | 2.5 (0.03) | 2.5 (0.03) | 2.5 (0.03) | 2.5 (0.03) | 2.5 (0.03) | 2.5 (0.03) |
|  | Median  (p1 – p99) | 3 (1 - 6) | 3 (1 - 4) | 2 (1 - 4) | 2 (1 - 4) | 2 (1 - 4) | 2 (1 - 4) | 2 (1 - 5) | 2 (1 - 5) | 3 (1 - 5) | 2 (1 - 5) | 2 (1 - 5) |
| p=0.6 | Mean (se) | 2.6 (0.03) | 2.5 (0.02) | 2.5 (0.02) | 2.3 (0.02) | 2.4 (0.03) | 2.5 (0.03) | 2.5 (0.03) | 2.5 (0.03) | 2.6 (0.03) | 2.5 (0.03) | 2.5 (0.03) |
|  | Median  (p1 – p99) | 3 (1 - 5) | 3 (1 - 4) | 2 (1 - 4) | 2 (1 - 4) | 2 (1 - 4) | 2 (1 - 5) | 2 (1 - 5) | 3 (1 - 5) | 3 (1 - 5) | 2 (1 - 5) | 2 (1 - 5) |
| p=0.7 | Mean (se) | 2.6 (0.03) | 2.5 (0.02) | 2.5 (0.02) | 2.3 (0.03) | 2.4 (0.03) | 2.5 (0.03) | 2.5 (0.03) | 2.5 (0.03) | 2.5 (0.03) | 2.5 (0.03) | 2.5 (0.03) |
|  | Median  (p1 – p99) | 3 (1 - 5) | 3 (1 - 4) | 2 (1 - 4) | 2 (1 - 4) | 2 (1 - 4) | 2 (1 - 5) | 2 (1 - 4.5) | 2 (1 - 5) | 3 (1 - 4) | 3 (1 - 5) | 2 (1 - 4) |
| p=0.8 | Mean (se) | 2.5 (0.03) | 2.5 (0.02) | 2.4 (0.03) | 2.4 (0.03) | 2.4 (0.03) | 2.5 (0.03) | 2.5 (0.03) | 2.5 (0.03) | 2.5 (0.03) | 2.5 (0.03) | 2.5 (0.03) |
|  | Median  (p1 – p99) | 2.5 (1 - 5) | 2 (1 - 4) | 2 (1 - 4) | 2 (1 - 4) | 2 (1 - 4) | 2 (1 - 4.5) | 2 (1 - 5) | 3 (1 - 5) | 3 (1 - 5) | 2 (1 - 5) | 2 (1 - 4) |
| p=0.85 | Mean (se) | 2.6 (0.03) | 2.6 (0.02) | 2.4 (0.02) | 2.4 (0.02) | 2.4 (0.03) | 2.5 (0.03) | 2.5 (0.03) | 2.6 (0.03) | 2.5 (0.03) | 2.5 (0.03) | 2.5 (0.03) |
|  | Median  (p1 – p99) | 3 (1 - 5) | 3 (1 - 4) | 2 (1 - 4) | 2 (1 - 4) | 2 (1 - 4) | 2 (1 - 4) | 2 (1 - 5) | 3 (1 - 5) | 3 (1 - 5) | 2 (1 - 5) | 2 (1 - 5) |
| p=0.9 | Mean (se) | 2.5 (0.03) | 2.5 (0.02) | 2.4 (0.02) | 2.3 (0.02) | 2.4 (0.03) | 2.5 (0.03) | 2.5 (0.03) | 2.5 (0.03) | 2.6 (0.03) | 2.5 (0.03) | 2.5 (0.03) |
|  | Median  (p1 – p99) | 2 (1 - 5) | 2 (1 - 4) | 2 (1 - 4) | 2 (1 - 4) | 2 (1 - 4) | 3 (1 - 4) | 3 (1 - 4) | 2 (1 - 5) | 3 (1 - 5) | 3 (1 - 5) | 3 (1 - 4) |
| p=0.95 | Mean (se) | 2.5 (0.03) | 2.5 (0.02) | 2.4 (0.03) | 2.4 (0.03) | 2.4 (0.03) | 2.5 (0.03) | 2.6 (0.03) | 2.5 (0.03) | 2.6 (0.03) | 2.5 (0.03) | 2.5 (0.03) |
|  | Median  (p1 – p99) | 2 (1 - 5) | 2 (1 - 4) | 2 (1 - 4) | 2 (1 - 4) | 2 (1 - 5) | 2 (1 - 4.5) | 3 (1 - 5) | 2 (1 - 5) | 3 (1 - 5) | 2 (1 - 5) | 3 (1 - 5) |
| Sample size 60, the expected number of participants allocated with the particular factor level n = 5 | | | | | | | | | | | | |
| p=0.5 | Mean (se) | 5.2 (0.05) | 5.1 (0.02) | 5.0 (0.03) | 5.0 (0.03) | 4.8 (0.04) | 4.8 (0.04) | 4.8 (0.04) | 4.8 (0.04) | 4.8 (0.04) | 4.8 (0.04) | 4.7 (0.04) |
|  | Median  (p1 – p99) | 5 (2 - 9) | 5 (3 - 7) | 5 (3 - 7) | 5 (3 - 7) | 5 (2 - 8) | 5 (2 - 8) | 5 (2 - 8) | 5 (2 - 7) | 5 (2 - 8) | 5 (2 - 7) | 5 (2 - 7.5) |
| p=0.6 | Mean (se) | 5.1 (0.05) | 5.1 (0.02) | 5.0 (0.03) | 5.0 (0.03) | 4.9 (0.03) | 4.7 (0.04) | 4.8 (0.03) | 4.8 (0.04) | 4.7 (0.03) | 4.8 (0.04) | 4.7 (0.04) |
|  | Median  (p1 – p99) | 5 (2 - 9) | 5 (4 - 7) | 5 (3 - 7) | 5 (2.5 - 7) | 5 (2 - 8) | 5 (2 - 8) | 5 (2 - 7) | 5 (2 - 8) | 5 (2 - 7.5) | 5 (2 - 8) | 5 (2 - 8) |
| p=0.7 | Mean (se) | 5.1 (0.05) | 5.0 (0.02) | 5.0 (0.03) | 5.0 (0.03) | 4.8 (0.03) | 4.9 (0.04) | 4.7 (0.04) | 4.8 (0.04) | 4.7 (0.04) | 4.8 (0.04) | 4.8 (0.04) |
|  | Median  (p1 – p99) | 5 (2 - 9) | 5 (4 - 6.5) | 5 (3 - 7) | 5 (3 - 7) | 5 (2 - 7) | 5 (2 - 8) | 5 (2 - 8) | 5 (2 - 8) | 5 (2 - 7) | 5 (2 - 7) | 5 (2 - 8) |
| p=0.8 | Mean (se) | 5.1 (0.04) | 5.0 (0.02) | 5.0 (0.03) | 5.0 (0.03) | 4.8 (0.03) | 4.8 (0.04) | 4.8 (0.04) | 4.8 (0.04) | 4.7 (0.04) | 4.7 (0.04) | 4.8 (0.04) |
|  | Median  (p1 – p99) | 5 (2 - 8.5) | 5 (4 - 6) | 5 (3 - 7) | 5 (3 - 7) | 5 (2 - 7) | 5 (2 - 8) | 5 (2 - 8) | 5 (2 - 7) | 5 (2 - 8) | 5 (2 - 7) | 5 (2 - 8) |
| p=0.85 | Mean (se) | 5.1 (0.04) | 5.0 (0.02) | 5.1 (0.03) | 5.1 (0.03) | 4.8 (0.03) | 4.8 (0.04) | 4.7 (0.04) | 4.8 (0.04) | 4.8 (0.04) | 4.8 (0.04) | 4.9 (0.04) |
|  | Median  (p1 – p99) | 5 (2 - 8) | 5 (4 - 6) | 5 (3 - 7) | 5 (3 - 7) | 5 (2 - 7) | 5 (2 - 8) | 5 (2 - 8) | 5 (2 - 7) | 5 (2 - 8) | 5 (2 - 7) | 5 (2 - 8) |
| p=0.9 | Mean (se) | 5.0 (0.04) | 5.0 (0.02) | 5.0 (0.03) | 5.0 (0.03) | 4.9 (0.03) | 4.8 (0.04) | 4.8 (0.04) | 4.8 (0.04) | 4.7 (0.04) | 4.7 (0.04) | 4.8 (0.04) |
|  | Median  (p1 – p99) | 5 (2 - 8) | 5 (4 - 6) | 5 (3 - 7) | 5 (3 - 7) | 5 (2 - 7) | 5 (2 - 8) | 5 (2 - 8) | 5 (2 - 7) | 5 (2 - 8) | 5 (2 - 7) | 5 (2 - 8) |
| p=0.95 | Mean (se) | 4.9 (0.04) | 5.0 (0.01) | 5.0 (0.03) | 5.1 (0.03) | 4.8 (0.04) | 4.8 (0.04) | 4.8 (0.04) | 4.8 (0.04) | 4.8 (0.04) | 4.8 (0.04) | 4.8 (0.04) |
|  | Median  (p1 – p99) | 5 (2 - 8) | 5 (4 - 6) | 5 (3 - 7) | 5 (3 - 7) | 5 (2 - 7) | 5 (2 - 8) | 5 (2 - 8) | 5 (2 - 8) | 5 (2 - 8) | 5 (2 - 8) | 5 (2 - 8) |
| Sample size 120, the expected number of participants allocated with the particular factor level n = 10 | | | | | | | | | | | | |
| p=0.5 | Mean (se) | 10.5 (0.08) | 10.1 (0.03) | 10.0 (0.04) | 10.0 (0.05) | 10.1 (0.05) | 10.1 (0.06) | 10.2 (0.06) | 9.9 (0.06) | 9.9 (0.06) | 10.1 (0.06) | 10.1 (0.06) |
|  | Median  (p1 – p99) | 10 (5 - 16.5) | 10 (8 - 12) | 10 (7 - 13) | 10 (7 - 13) | 10 (6 - 14) | 10 (6 - 14) | 10 (6 - 14.5) | 10 (5 - 14) | 10 (5 - 15) | 10 (6 - 15) | 10 (6 - 15) |
| p=0.6 | Mean (se) | 10.3 (0.07) | 10.1 (0.03) | 10.0 (0.04) | 10.2 (0.05) | 10.1 (0.05) | 10.1 (0.06) | 10.2 (0.06) | 9.9 (0.06) | 9.9 (0.06) | 9.9 (0.06) | 10.1 (0.07) |
|  | Median  (p1 – p99) | 10 (5 - 15.5) | 10 (8 - 12) | 10 (7 - 13) | 10 (6 - 14) | 10 (7 - 14) | 10 (6 - 14) | 10 (6 - 14) | 10 (5 - 14) | 10 (5 - 15) | 10 (5.5 - 14) | 10 (5 - 15) |
| p=0.7 | Mean (se) | 10.2 (0.07) | 10.1 (0.02) | 10.1 (0.04) | 10.1 (0.05) | 10.0 (0.05) | 10.1 (0.06) | 10.1 (0.06) | 9.9 (0.06) | 9.9 (0.06) | 10.0 (0.06) | 10.0 (0.06) |
|  | Median  (p1 – p99) | 10 (5 - 15) | 10 (8 - 12) | 10 (7 - 13) | 10 (6 - 14) | 10 (6 - 14) | 10 (6 - 14) | 10 (6 - 14.5) | 10 (5 - 14) | 10 (5 - 15) | 10 (6 - 14) | 10 (5 - 15) |
| p=0.8 | Mean (se) | 10.2 (0.07) | 10.0 (0.02) | 10.0 (0.04) | 10.2 (0.05) | 10.1 (0.06) | 10.1 (0.06) | 10.2 (0.06) | 10.0 (0.06) | 9.8 (0.06) | 9.9 (0.06) | 10.1 (0.07) |
|  | Median  (p1 – p99) | 10 (5 - 16) | 10 (8 - 12) | 10 (7 - 13) | 10 (7 - 14) | 10 (6 - 14) | 10 (6 - 14) | 10 (6 - 14) | 10 (6 - 15) | 10 (5 - 15) | 10 (5.5 - 14) | 10 (5 - 15) |
| p=0.85 | Mean (se) | 10.1 (0.07) | 10.0 (0.02) | 10.0 (0.04) | 10.1 (0.05) | 10.2 (0.05) | 10.2 (0.05) | 10.1 (0.06) | 9.9 (0.06) | 9.9 (0.06) | 9.9 (0.06) | 10.1 (0.07) |
|  | Median  (p1 – p99) | 10 (5 - 15) | 10 (8 - 12) | 10 (7 - 13) | 10 (7 - 14) | 10 (6 - 14) | 10 (6 - 14) | 10 (6 - 14) | 10 (5 - 14) | 10 (5 - 15) | 10 (6 - 15) | 10 (5 - 15) |
| p=0.9 | Mean (se) | 10.1 (0.07) | 10.0 (0.02) | 10.1 (0.04) | 10.1 (0.05) | 10.2 (0.05) | 10.2 (0.06) | 10.2 (0.06) | 10.0 (0.06) | 10.0 (0.06) | 9.9 (0.06) | 10.0 (0.06) |
|  | Median  (p1 – p99) | 10 (5 - 15) | 10 (8 - 12) | 10 (7 - 13) | 10 (7 - 14) | 10 (6 - 14) | 10 (6 - 15) | 10 (6 - 14.5) | 10 (6 - 14) | 10 (5.5 - 14) | 10 (5 - 15) | 10 (6 - 15) |
| p=0.95 | Mean (se) | 10.1 (0.07) | 10.0 (0.02) | 10.0 (0.04) | 10.1 (0.05) | 10.1 (0.06) | 10.2 (0.06) | 10.2 (0.06) | 10.0 (0.06) | 9.8 (0.06) | 9.9 (0.06) | 10.1 (0.07) |
|  | Median  (p1 – p99) | 10 (5 - 15) | 10 (8 - 12) | 10 (7 - 13) | 10 (6 - 14) | 10 (6 - 14) | 10 (6 - 14) | 10 (6 - 14) | 10 (5 - 14) | 10 (5 - 14) | 10 (5.5 - 14) | 10 (5 - 15) |

**Appendix C**

Figure 1: Randomisation distributions with 1:2:3 allocation ratio, mean differences for two treatments with smaller-allocation ratio (n = 5 vs 10, 10 vs 20 and 20 vs 40)


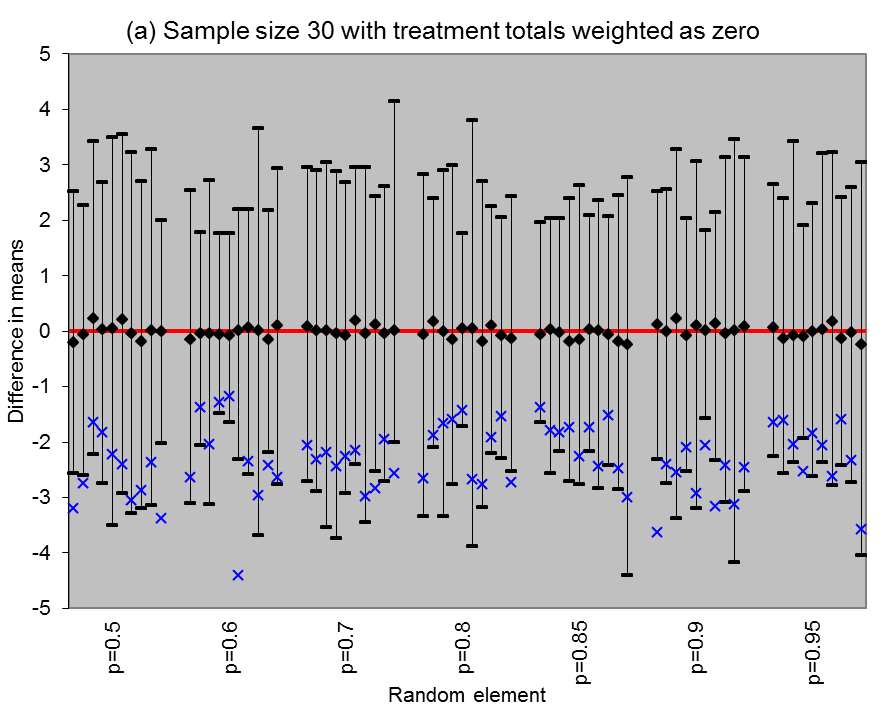

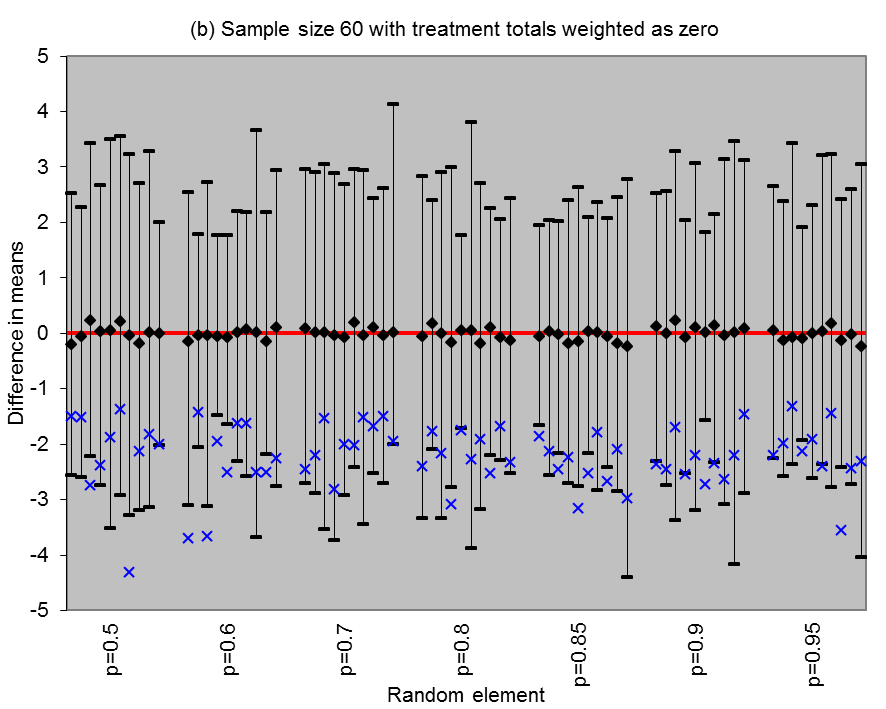

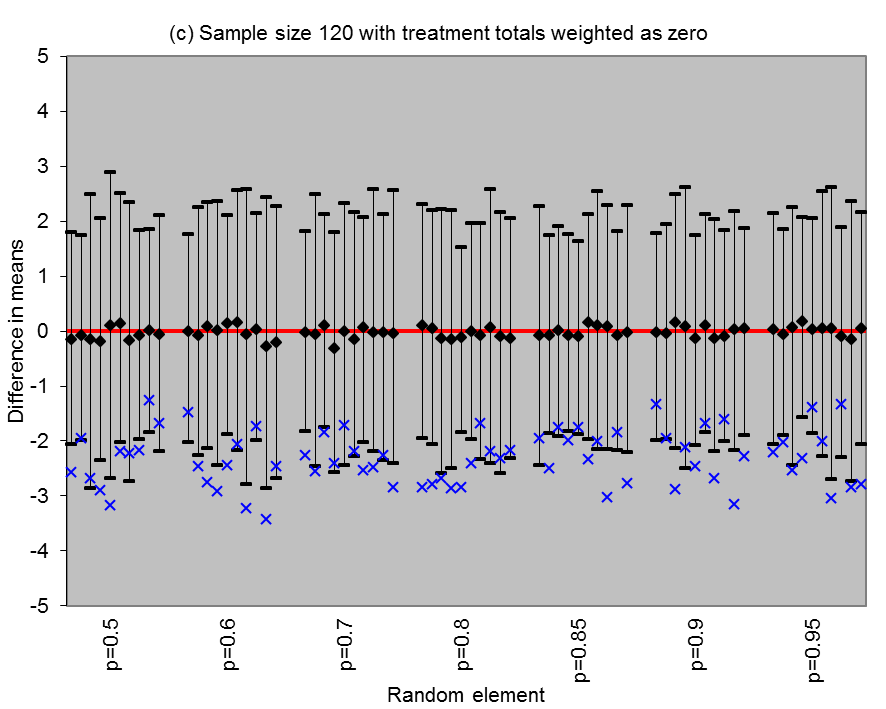

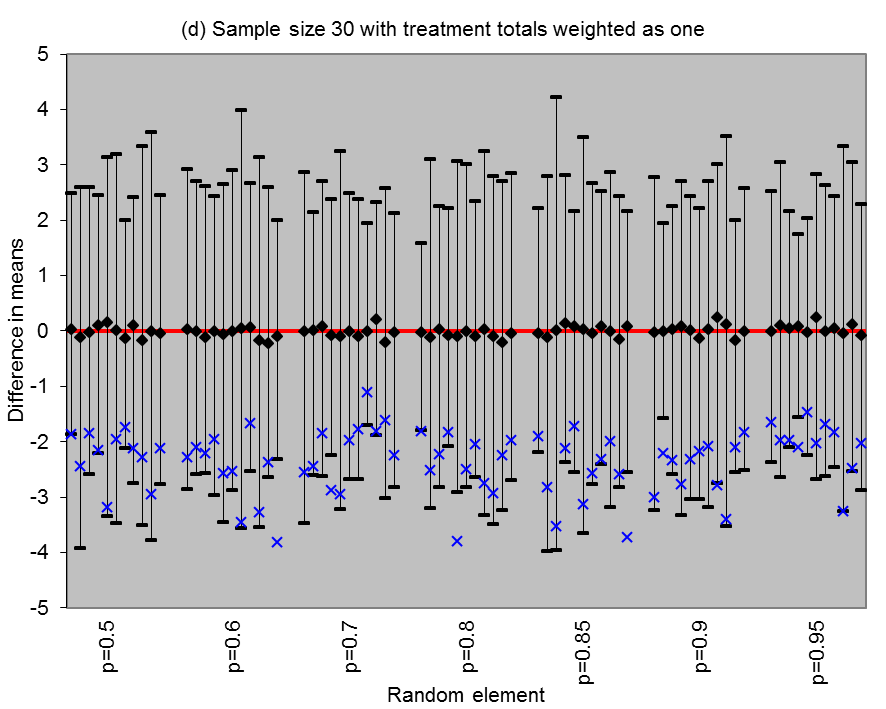

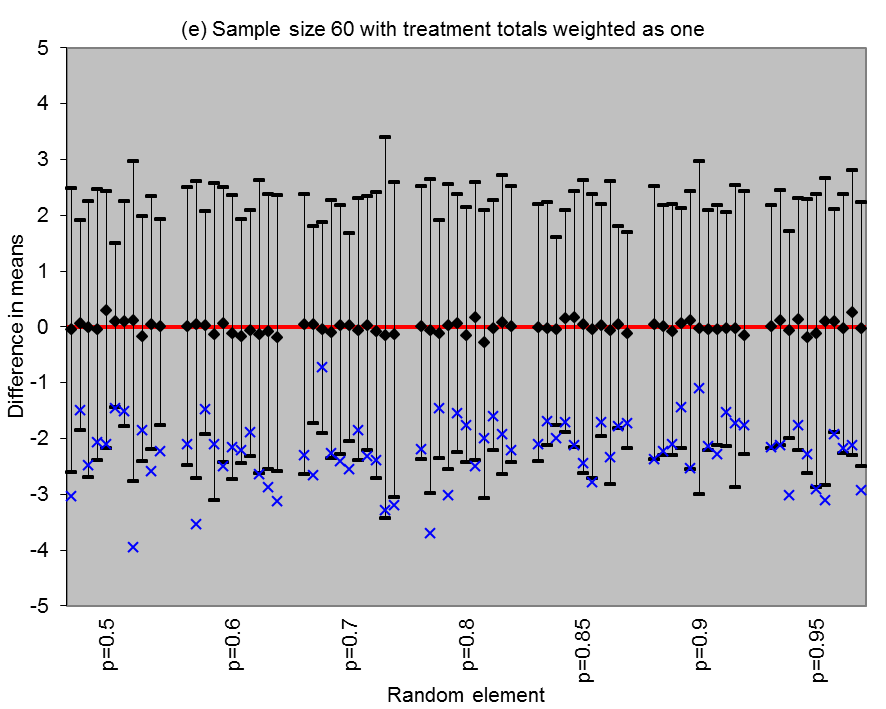

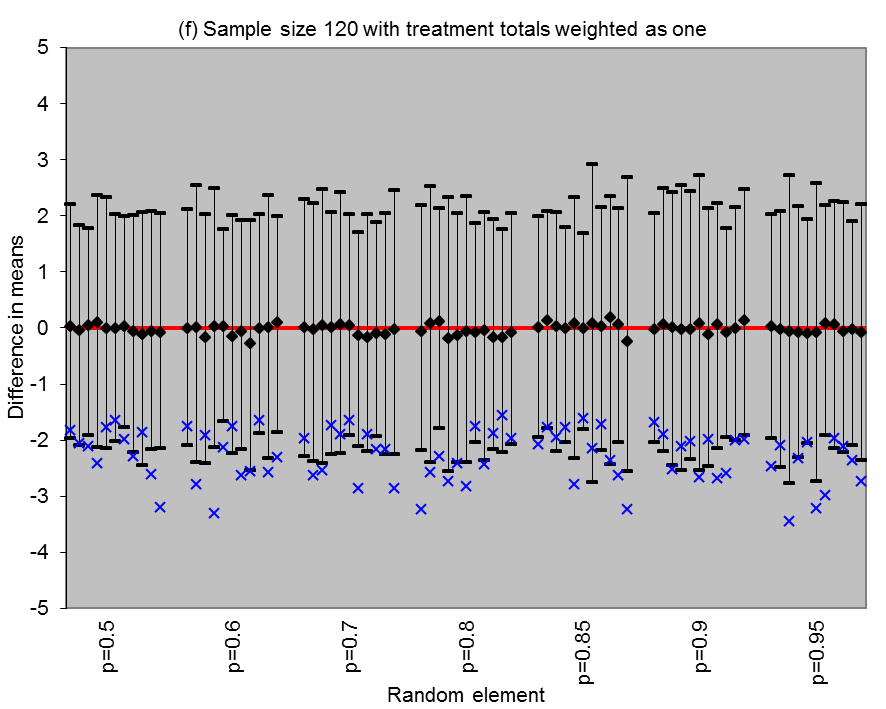

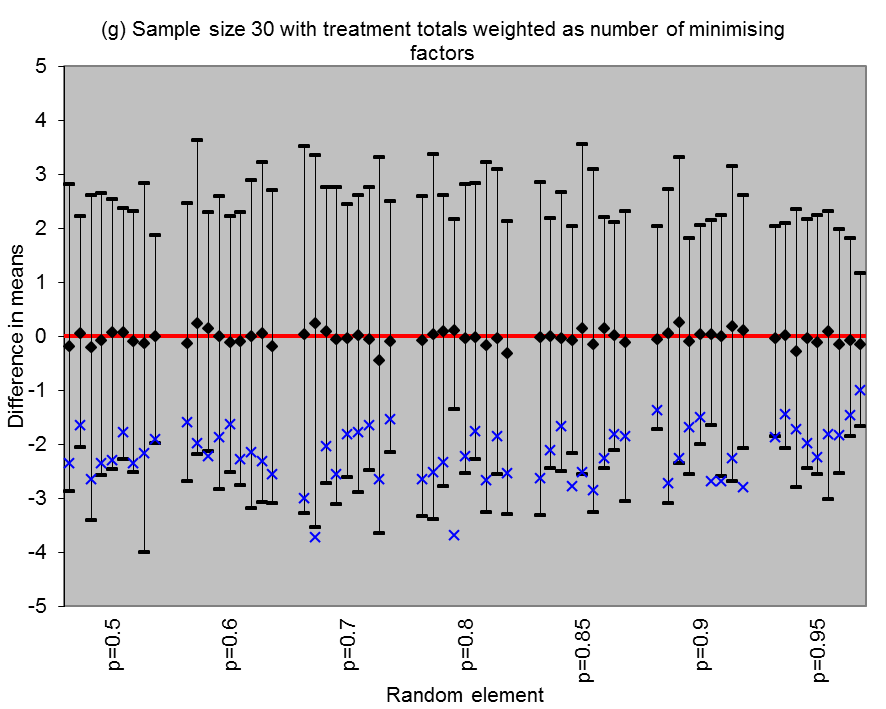

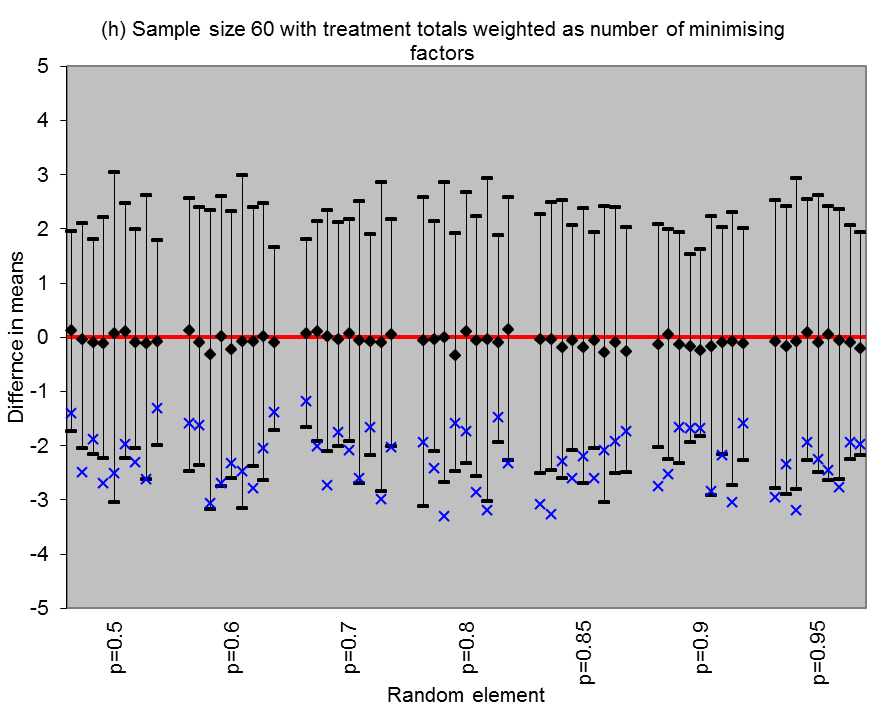

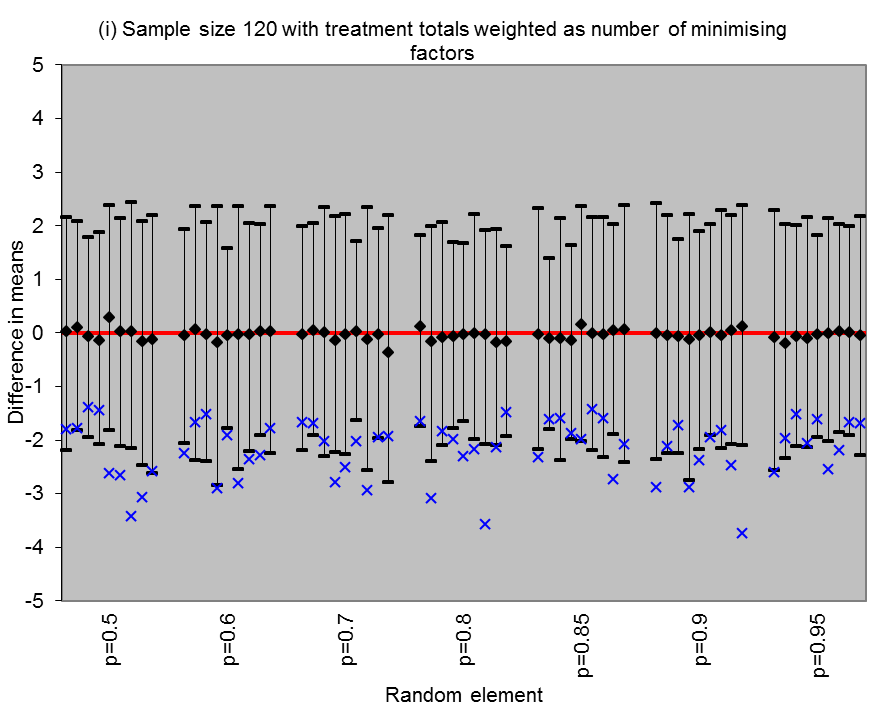

Supplement: Additional file 1: Table S1. — Treatment-balancing properties of 1:2:3 sequence balance minimisation with one to ten factors with two levels, treatment totals weighted as zero, random element = 0.95 to 0.5. Table S2. Treatment-balancing properties of 1:2:3 sequence balance minimisation with zero (treatment totals only) to ten factors with two levels, treatment totals weighted as one, random element = 0.95 to 0.5. Table S3. Treatment-balancing properties of 1:2:3 sequence balance minimisation with zero (treatment totals only) to ten factors with two levels, treatment totals weighted as total number of minimisation factors, random element = 0.95 to 0.5. Table S4. Factor-balancing properties of 1:2:3 sequence balance minimisation with one to ten factors with two levels, treatment totals weighted as zero, random element = 0.95 to 0.5. Table S5. Factor-balancing properties of 1:2:3 sequence balance minimisation with one to ten factors with two levels, treatment totals weighted as one, random element = 0.95 to 0.5. Table S6. Factor-balancing properties of 1:2:3 sequence balance minimisation with one to ten factors with two levels, treatment totals weighted as total number of minimisation factors, random element = 0.95 to 0.5. Figure S1. Randomisation distributions with 1:2:3 allocation ratio, mean differences for two treatments with smaller-allocation ratio (n = 5 vs 10, 10 vs 20, and 20 vs 40). (DOC 1426 kb) [file 13063_2017_1942_MOESM1_ESM.doc]
